# Dialumene as a Dimeric or Monomeric Al Synthon for C–F Activation in Monofluorobenzene

**DOI:** 10.1021/jacs.4c08171

**Published:** 2024-08-06

**Authors:** Xufang Liu, Shicheng Dong, Jun Zhu, Shigeyoshi Inoue

**Affiliations:** † TUM School of Natural Sciences, Department of Chemistry, Institute of Silicon Chemistry and Catalysis Research Center, Technische Universität München, Lichtenbergstraße 4, Garching bei München 85748, Germany; ‡ State Key Laboratory of Physical Chemistry of Solid Surfaces, Collaborative Innovation Center of Chemistry for Energy Materials (iChem), Fujian Provincial Key Laboratory of Theoretical and Computational Chemistry, College of Chemistry and Chemical Engineering, Xiamen University, Xiamen 361005, China; § School of Science and Engineering, The Chinese University of Hong Kong, Shenzhen 518172, China

## Abstract

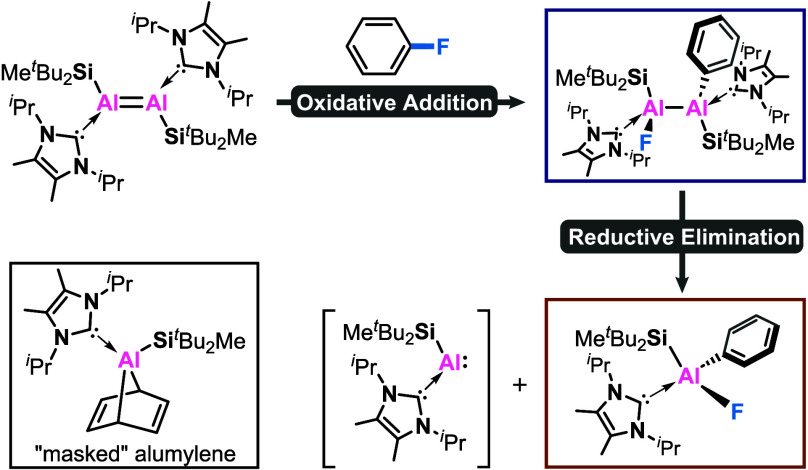

The activation of C–F bonds has long been regarded as the subject of research in organometallic chemistry, given their synthetic relevance and the fact that fluorine is the most abundant halogen in the Earth’s crust. However, C–F bond activation remains a largely unsolved challenge due to the high bond dissociation energies, which was historically dominated by transition metal complexes. Main group elements that can cleave unactivated monofluorobenzene are still quite rare and restricted to s-block complexes with a biphilic nature. Herein, we demonstrate an Al-mediated activation of monofluorobenzene using a neutral dialumene, allowing for the synthesis of the formal oxidative addition products at either double or single aluminum centers. This neutral dialumene system introduces a novel methodology for C–F bond activation based on formal oxidative addition and reductive elimination processes around the two aluminum centers, as demonstrated by combined experimental and computational studies. A “masked” alumylene was unprecedentedly synthesized to prove the proposed reductive elimination pathway. Furthermore, the synthetic utility is highlighted by the functionalization of the resulting aryl-aluminum compounds.

## Introduction

The activation and functionalization of robust carbon–fluorine bonds have recently drawn growing attention as a strategy for building molecular complexity and accessing value-added materials. One reason is that many fluorinated compounds are commercially available and thus serve as starting points for synthetic diversification. The number of registered halogenated compounds is as follows:^1^ Ar–I (954,235), Ar–Br (6,588,400), Ar–Cl (14,690,519), and Ar–F (15,536,939), which revealed that fluoroarenes are the largest group of commercially available halogenated arenes.^2^ In addition, introduction of fluorine substituents often imparts a great enhancement in the chemical stability, lipophilicity, and bioavailability, which in turn leads to extensive applications in agrochemical, pharmaceutical, and organic materials science.^3^

**Scheme 1:**
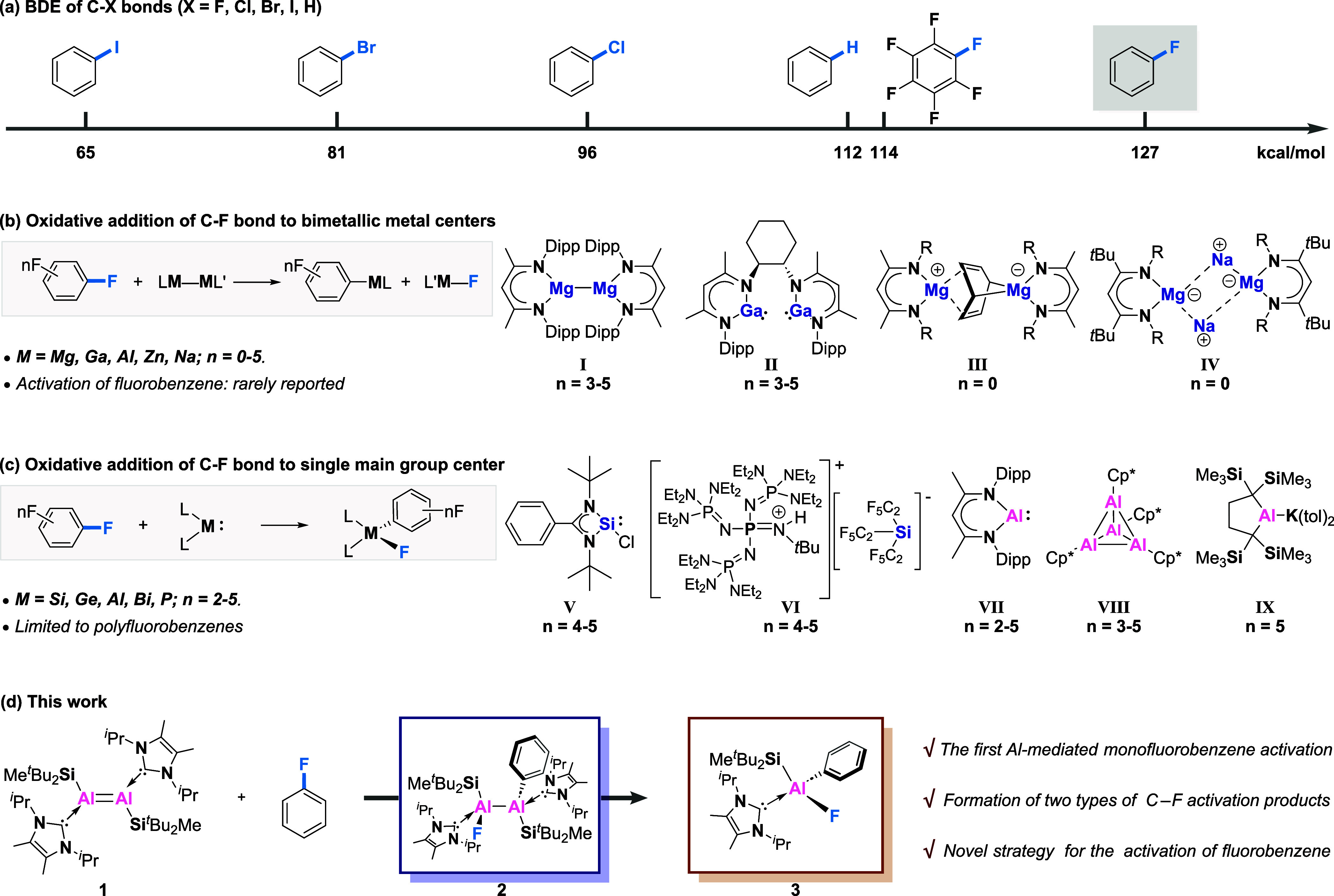
State-of-the-Art for Main Group Element Mediated Oxidative Cleavage of Aryl C–F Bonds

Despite all these benefits, the activation of C–F bonds in fluoroarenes (Ar–F) remains one of the major challenges in modern synthetic chemistry, mainly due to the abnormal strength of the bond energies compared to their relatively activated halogenated congeners (Ar–X, X = Cl, Br, I). C–F bonds are typically described as the strongest bonds that carbon can form (Scheme 1a).^4^ For fluoroaromatics, the C–F bond energies strengthen systematically with decreasing fluorination. For instance, the C–F bond energy in C_6_F_6_ is 114 kcal/mol^5^ compared to 127 kcal/mol in C_6_H_5_F^6^ (Scheme 1a). This fact results in a more challenging C–F activation of partially fluorinated aromatics compared to perfluorinated substrates, which in many cases can be accompanied by the kinetically more favored aryl C–H activation (C–H bond energy in benzene is 112 kcal/mol).^7,8^

The vast majority of C–F bond activation relies on the use of transition-metal complexes enabled by the redox versatilities of these systems,^9^ but the past decade has witnessed the large potential of main-group elements to split strong σ-bonds,^10^ especially C–F bonds.

In main group chemistry, C–F activation typically occurs through two modes.^11^ One mode is hydrodefluorination of C–F bonds using metal hydride complexes in their higher oxidation states via nucleophilic aromatic substitution.^12^ The C–F bond activation in monofluorobenzene was implemented by a series of well-defined or less-defined s-block metal hydrides, potentially driven by the electrophilicity of the metal center, the hydricity of the hydride ligand, and the elimination of an extremely stable C_6_H_6_ fragment.

The other mode is the oxidative cleavage of C–F bonds using low-valent bimetallic or single-site complexes. In this case, the aryl-metal species generated can be seen as a C_6_H_5_^–^ source capable of transferring the aryl group to electrophiles, thereby providing a platform for converting readily available fluorinated compounds into a rich array of valuable organic compounds.

As shown in Scheme 1b, cooperative oxidative addition of C–F bonds across bimetallic metal centers generates aryl-metal species and fluoride-metal species simultaneously. Bimetallic systems such as β-diketiminate Mg^I^ complex **I** and dinuclear Ga^I^ complex **II**, have shown capabilities for C–F bond activation of various per- and poly fluorinated arenes by the groups of Crimmin^13^ and Kretschmer.^14^ However, examples of the cleavage of unactivated monofluorobenzene are scarcely reported. Harder and coworkers isolated **III**, a complex formally consisting of a (^DIPeP^BDI)Mg^+^ cation and a (^DIPeP^BDI)Mg-norbornadiene anion, and discovered that it reductively cleaved monofluorobenzene, the most challenging substrate for aryl C–F bond activation, albeit under forcing conditions of 100 °C for 5 days.^15^ Later on, Harder et al. reported a magnesyl sodium complex **IV** featuring two anionic (DipepL)Mg^–^ fragments bridged by two Na^+^ cations, which can activate monofluorobenzene to form Mg^II^-aryl species with the concomitant elimination of NaF under very mild conditions (20 °C, 10 min).^16^ The exceptional reactivities of **III** and **IV** could be ascribed to their biphilic nature, stemming from the electrophilicity of the Mg/Na cation and the nucleophilicity of the Mg anion.

As shown in Scheme 1c, oxidative addition of C–F bonds to a single main group center forms M–C and M–F bonds at the same central atom, which commonly employs low-valent single-site complexes such as silylene (**V**),^17^ silanide anion (**VI**),^18^ monomeric Al complex (**VII**),^19^ tetrameric (Cp*Al)^4^ (**VIII**)^20^ and aluminyl potassium complex (**IX**).^21^ However, the scope is now limited to more activated poly fluorinated substrates with at least three fluorine substituents.^22^

As alluded to above, the activation of monofluorobenzene is particularly challenging and is currently achieved either by metal hydrides or by ionic bimetallic systems enabled by their biphilic nature, which mostly involve s-block elements.^15,16^ To the best of our knowledge, no p-block elements have been involved in the cleavage of unactivated monofluorobenzene without the assistance of d-block metals.^23^ It is also worth noting that the oxidative cleavage of mono and difluorobenzenes at a single main group center has yet to be accomplished.

In 2017, our group reported the first isolation of a neutral dialumene, which can behave as transition metal mimics to activate various unsaturated organic molecules.^24^ The highly reactive character of dialumene makes it a potent candidate for the activation of inert C–F bonds. Herein, we present an Al-mediated monofluorobenzene activation by using a neutral dialumene, which initially forms the dialane Al^II^ product **2** via C–F bond addition to the Al=Al double bond followed by Al–Al bond cleavage to generate the monomeric Al^III^ product **3** (Scheme 1d). As such, this neutral dialumene can serve as a dimeric or monomeric aluminyl synthon, enabling the generation of the formal oxidative addition products at either double or single aluminum centers.

## Results and Discussion

**Scheme 2:**
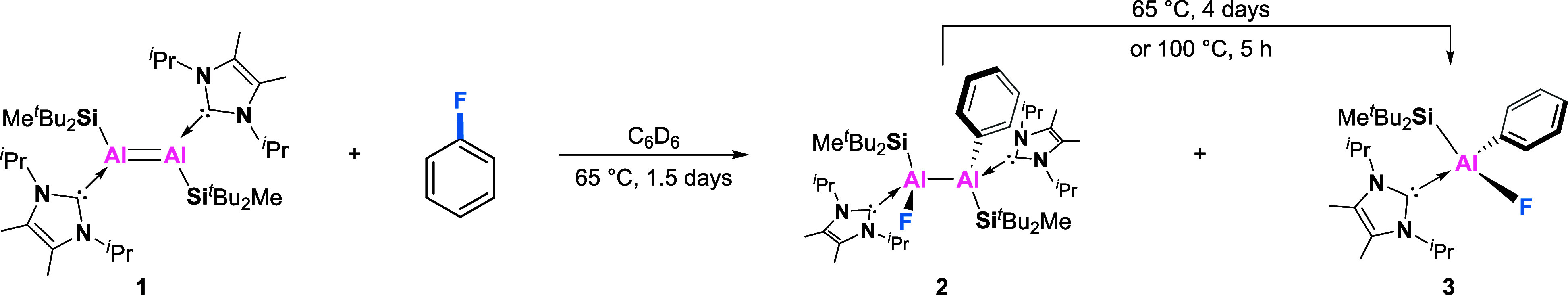
Dialumene-Mediated Activation of Monofluorobenzene

The reaction of dialumene **1** with excess monofluorobenzene in C_6_D_6_ occurred at 65 °C, a color change was observed from dark purple to light yellow after 1.5 days. ^1^H and ^19^F NMR spectra revealed two distinct sets of resonances in a ratio of 3:1, implying the generation of two new aluminum fluoride species (Scheme 2). After workup and recrystallization from a pentane solution, compound **2**, identified as the major species in the reaction mixture, was separated as colorless crystals in 54% yield and fully characterized by ^1^H, ^13^C, and ^19^F NMR spectroscopy.

**Figure 1:**
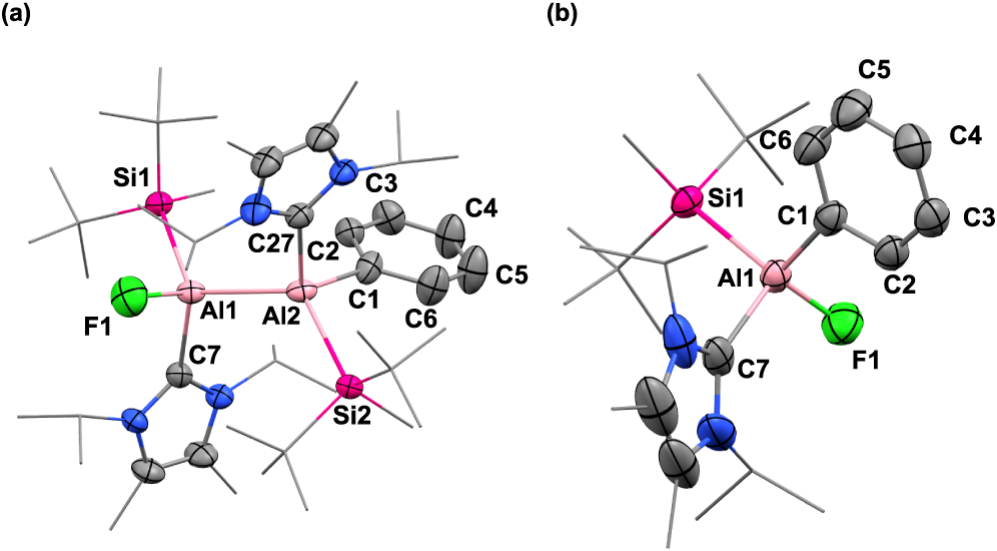
(a) Molecular structure of **2**. (b) Molecular structure of **3**. Ellipsoids set at 50% probability. Hydrogen atoms are omitted for clarity. Selected bond lengths [Å] and angles [deg]: **2** Al1–F1 1.712(4), Al1–Al2 2.698(2), Al2–C1 2.059(5), Al1–C7 2.110(4), Al2–C27 2.126(4), Al1–Si1 2.566(2), Al2–Si2 2.565(4); F1–Al1–Al2 110.41(1), Al1–Al2–C1 106.16(2), Si1–Al1–C7 104.64(1), Si2–Al2–C27 108.15(2), F1–Al1–Al2–C1 10.97(2); **3**: Al1–F1 1.689(2), Al1–C1 1.994(3), Al1–C7 2.073(3), Al1–Si1 2.487(2); F1–Al1–C1 106.70(1), Si1–Al1–C7 111.26(1).

The ^19^F NMR spectra feature a broad singlet at −167 ppm due to *J* coupling to ^27^Al (*I* = 5/2), consistent with those found in NacNacAlF(C_6_F_5_).^19^ The molecular structure of **2** was further determined by single-crystal X-ray diffraction (SC-XRD) analysis (Figure 1a). The loss of double bond character was confirmed by the considerable elongation of the Al–Al bond length (2.694(2) Å) compared to dialumene **1** (2.3943(16) Å). Also noted to occur is the markedly longer Al1–Al2 bond (2.694(2) Å) than the equivalent bond in previously reported dialumina-cyclobutane compound (2.6503(10) Å),^24a^ likely arising from the effect of steric around the aluminum centers. The Al2–C1 and Al1–F1 bond lengths are 2.057(5) Å and 1.713 (4) Å, respectively, also longer than the corresponding distances in NacNacAlF(C_6_F_5_) (1.9993(18) and 1.6582(11)).^19^

More interestingly, the slow transformation of **2** into **3** was detected by ^1^H NMR monitoring after heating the resultant 3:1 reaction mixture at 65 °C for 4 days (or 100 °C, 5 h). **3** was isolated in 43% yield by recrystallization in pentane and characterized by multinuclear NMR spectroscopy and X-ray diffraction (Figure 1b). The ^19^F resonance at −170 ppm is assigned to the fluoride attached to the aluminum center. The Al1–C1 and Al1–F1 bond lengths are 1.994(3) Å and 1.689(2) Å, respectively, comparable to the values obtained for other aluminum fluoride species as reported before.^19^ Monomeric Al^III^**3** can be viewed as the formal oxidative addition product at a single aluminum center, suggesting that dialumene **1** can be used as a surrogate for the monomeric aluminyl complex. The isolation of **3** marks a pioneering example in main group chemistry, with no precedents for the oxidative cleavage of monofluorobenzene at a single main group center.

**Figure 2:**
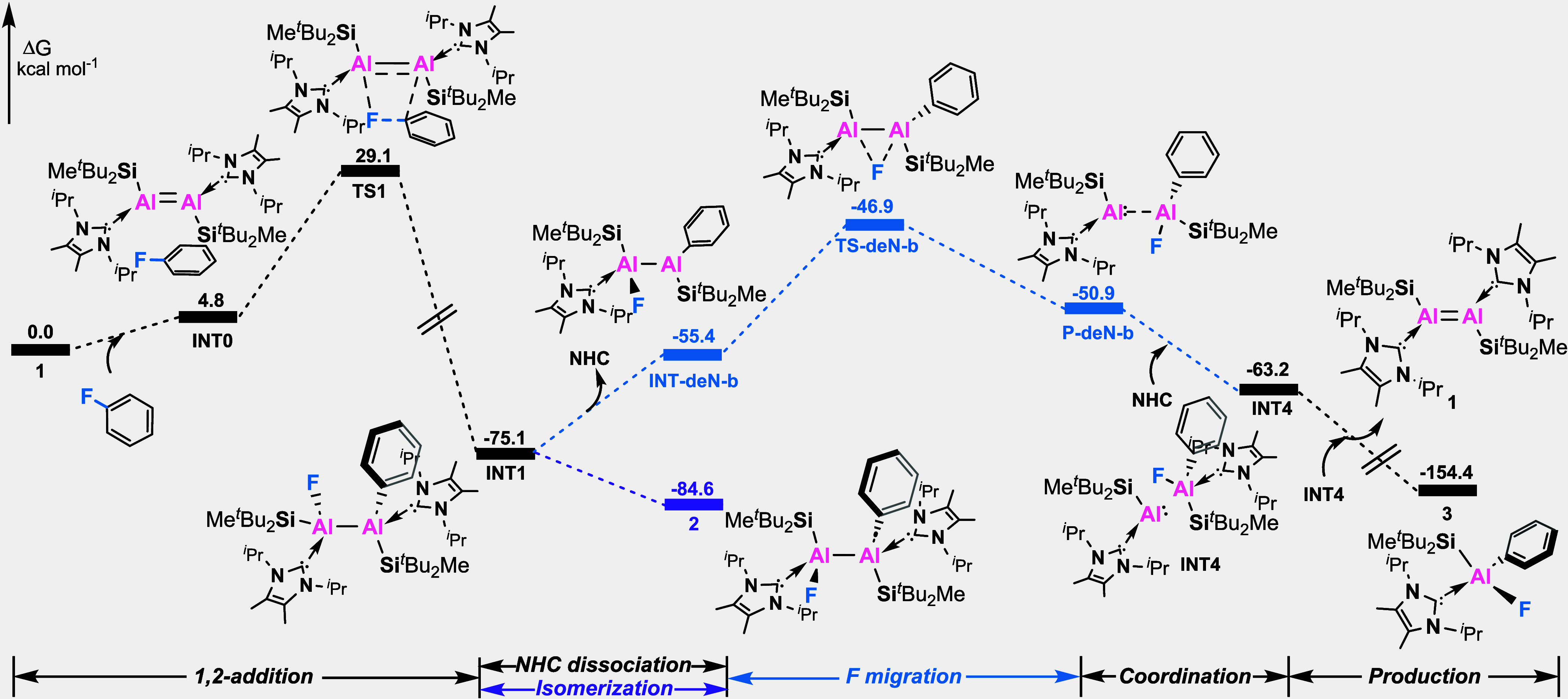
Proposed mechanism and potential energy surface of reaction process at the B3LYP(D3)/def2-TZVP//B3LYP(D3)/def2-SVP level. The Gibbs energies are given in kcal/mol.

The remarkable ability of dialumene to split monofluorobenzene can be attributed to its significantly low HOMO–LUMO gap (2.29 eV).^24a^ The conversion of dialumene **1** to dialane **2** was thought to undergo a formal oxidative addition pathway, which is a typical reactivity of dialumene compounds. DFT calculations revealed that **2** can be formed through a *syn*-addition of the C–F bond across the Al=Al double bond and a subsequent fast isomerization, with an overall energy barrier of 29.1 kcal/mol (Figure 2), which does match well with the temperature required for the reaction experimentally.

Next, the reaction mechanism underlying the transformation from dialane **2** to monomeric compound **3** was investigated. The effect of fluorobenzene was first explored by control experiments. We heated a C_6_D_6_ solution of **2** at 65 °C in the absence of C_6_H_5_F, surprisingly, **3** was almost quantitatively formed. Besides, the addition of C_6_H_5_F has no impact on both the reaction rate and the yield. In combination, these results suggested that fluorobenzene should not be involved in the formation of the final product **3**. Furthermore, DFT calculations indicated that the direct oxidative addition of the C–F bond to the Al–Al unit via a concerted four-membered transition state to generate **3** is kinetically unfavorable with a very high reaction barrier of 75.6 kcal/mol, due to severe steric repulsions imposed by the bulky silyl groups (Figure S23). This calculated result gave further evidence to rule out the role of fluorobenzene in the transformation of **2** (Al^II^) to **3** (Al^III^), which strongly indicated the elimination of a low-valent species during this process.

**Scheme 3:**
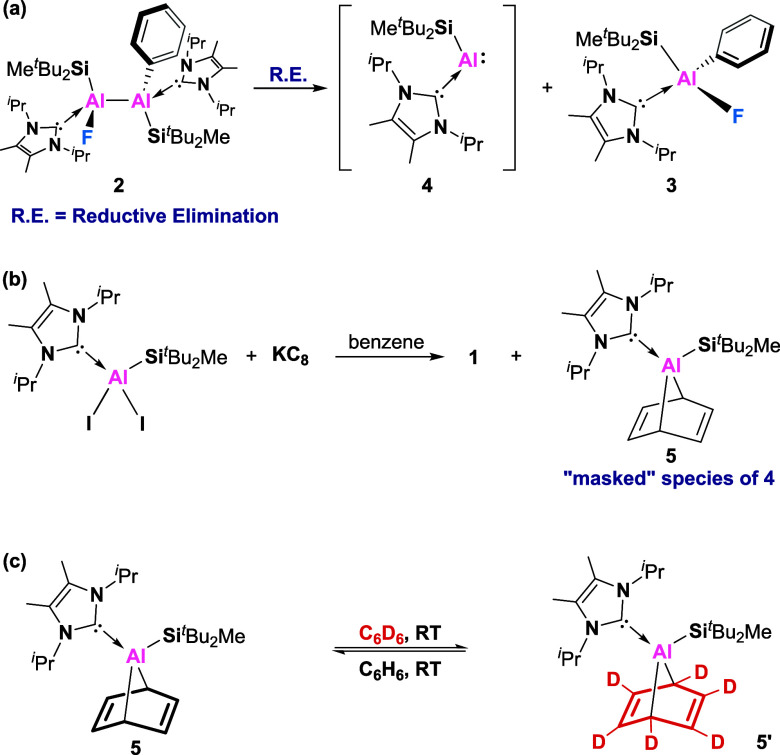
Proposed Reductive Elimination Pathway for the Transformation of **2** to **3**

**Figure 3:**
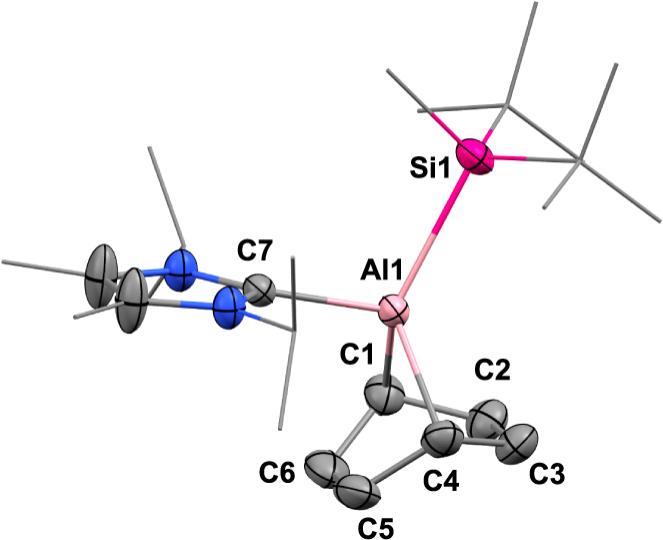
Molecular structure of **5**. Ellipsoids set at 50% probability. Hydrogen atoms are omitted for clarity. Selected bond lengths [Å] and angles [deg]: Al1–C1 2.062, Al1–C4 2.062, Al1–C7 2.064, Al1–Si1 2.487, C1–C2 1.500(3), C2–C3 1.331(3), C3–C4 1.500(3), C4–C5 1.500(3), C5–C6 1.326(3), C6–C1 1.500(3); C1–Al1–C4 76.81, Si1–Al1–C7 106.06.

Therefore, we proposed that compound **2** (Al^II^) disproportionates to form compound **3** (Al^III^) and monomeric aluminyl (Al^I^) species **4** via Al–Al bond cleavage (Scheme 3a), which is considered as a formal reductive elimination process around the Al–Al core, akin to that seen in the reversible reductive elimination of aluminum(II) dihydrides.^10f,25^ Oxidative addition to Al^I^ compounds is now well-established, whereas reverse reductive elimination is much less developed because it is much more challenging for main-group elements to access their lower oxidation states as compared to transition metals.^26^ The above reaction hence represents a unique case for reductive elimination at dialumina-centers. The longer distance of the Al–Al bond in **2** as mentioned above, makes the Al–Al moiety exhibit a labile behavior, thus inducing the facile cleavage of the Al–Al bond to produce **3**. Unfortunately, attempts to trap the dissociated aluminum species **4** using NHC, sulfur, selenium, or a bulky alkyne were all unsuccessful, due to its inherent thermal instability. To probe the possibility of generating **4**, compound **5** was independently synthesized as a “masked” species of **4**, which represents the first example of a “masked” alumylene (Scheme 3b).^27^ The structure of **5** was confirmed by X-ray crystallography, which is shown to result from a [4 + 1] cycloaddition of benzene to the Al^I^ center (Figure 3). The properties of compound **5** as a “masked” species of **4** were well demonstrated through intermolecular exchange reaction with C_6_D_6_ solvent (Scheme 3c).^28^ Moreover, it was found that **5** is highly unstable even at room temperature and underwent complete decomposition at 40 °C within 1 day, leading to free NHC and other ill-defined products.

DFT calculations were conducted to provide additional support for the proposed reductive elimination process (Figure 2). A NHC dissociation and recoordination pathway was calculated to be the optimal reaction process. Specifically, **INT1** first undergoes NHC dissociation, resulting in the formation of **INT-deN-b**. Subsequently, **INT-deN-b** undergoes a fluoride-migration to form intermediate **P-deN-b** through transition state **TS-deN-b** with a reaction barrier of 28.2 kcal/mol. After that, the dissociated NHC will rapidly coordinate to generate a thermodynamically more stable intermediate **INT4** with an exergonicity of 63.2 kcal/mol. Then, self-dimerization of monomeric Al^I^ species is assumed to form dialumene **1**, along with the generation of the thermodynamically most stable product **3** with an exergonicity of 154.4 kcal/mol. The regenerated dialumene **1** was not detected during the reaction process from **2** to **3**, due to the thermal instability of dialumene (dialumene underwent complete decomposition at 65 °C within a span of 5 days, forming free NHC and other ill-defined products). In addition, according to the kinetic profiles of the reactions from **2** to **3**, we determined the activation energy for the rate-determining step to be 30.1 kcal/mol, which is close to the calculated value (Figure S19).

Notably, no hints of the competing C–H activation products were obtained either from in situ spectroscopic measurements or in the isolated products, demonstrating the exclusive selectivity of this dialumene system toward C–F activation. The calculation results indicated that C–H activation is less favorable in both the thermodynamics (Δ*G*_C–H_ = −21.2 kcal/mol, Δ*G*_C–F_ = −75.1 kcal/mol) and kinetics (Δ*G*_C–H_‡ = 44.5 kcal/mol, Δ*G*_C–F_‡ = 29.1 kcal/mol) compared to C–F activation (Figure S25). The reason can be attributed to the formation of a much stronger Al–F bond (BDE: 160.4 kcal/mol) compared to the Al–H bond (BDE: 88.1 kcal/mol) (Figure S20), in line with our previous studies.^29^

As discussed above, by virtue of the high reactivity of dialumene and the labile nature of the Al–Al bond in the dialane intermediate, this neutral dialumene system undoubtedly offers a novel strategy for fluorobenzene activation based on formal oxidative addition and reductive elimination at the two aluminum centers. This largely distinguishes it from established systems that use metal hydrides or ionic bimetallic complexes with a biphilic nature. Particularly noteworthy, oxidative cleavage of monofluorobenze, which cannot be realized by main group single-site complexes, was indirectly achieved by dialumene through a “hidden” reductive elimination process.

**Scheme 4:**
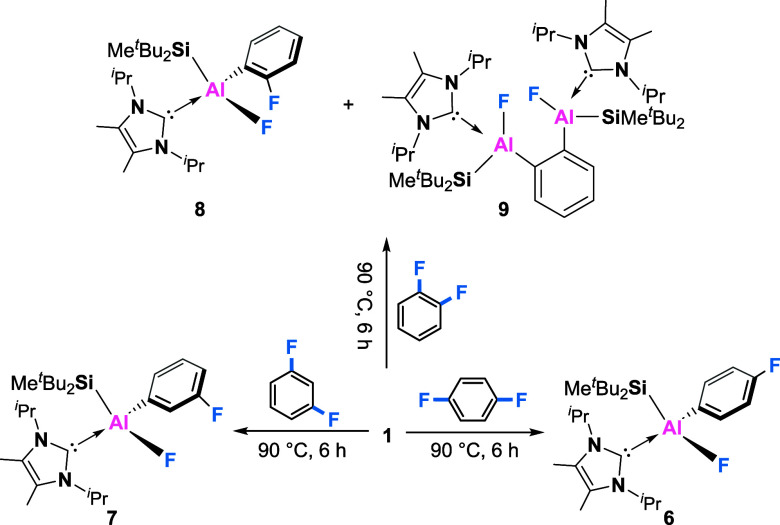
Dialumene-Mediated Activation of Difluorobenzenes

**Figure 4:**
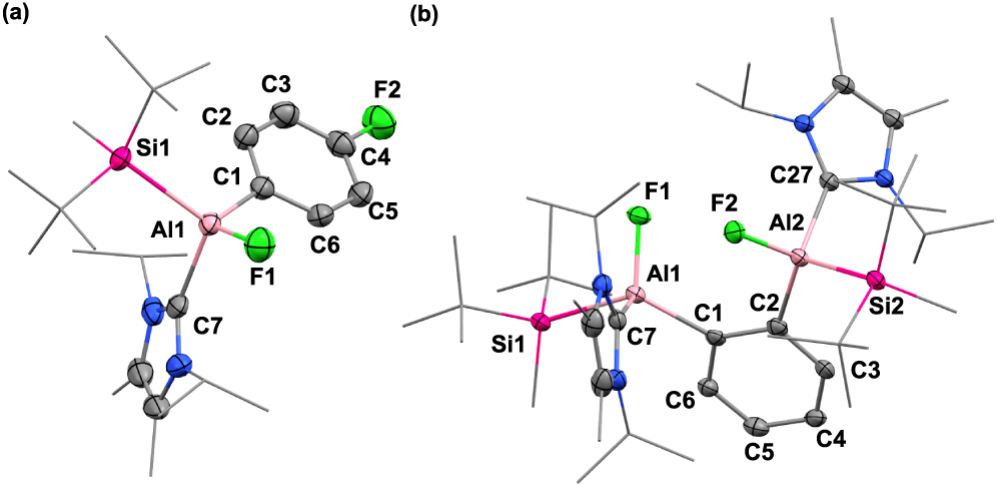
(a) Molecular structure of **6**. (b) Molecular structure of **9**. Ellipsoids set at 50% probability. Hydrogen atoms are omitted for clarity. Selected bond lengths [Å] and angles [deg]: **6**: Al1–F1 1.686(1), Al1–C1 1.995(2), C4–F2 1.362(2), Al1–C7 2.071(2), Al1–Si1 2.489(8); F1–Al1–C1 106.49(7), Si1–Al1–C7 110.63(6); **9**: Al1–F1 1.693(2), Al1–C1 2.017(2), Al2–F2 1.693(2), Al2–C2 1.986(2), Al1–C7 2.079(3), Al2–C27 2.077(3), Al1–Si1 2.537(1), Al2–Si2 2.507(1); F1–Al1–C1 114.64(9), F1–Al1–C1 109.82(9), Al–C1–C2 127.51(2), C1–C2–Al2 123.73(2), Si1–Al1–C7 108.49(7), Si2–Al2–C27 110.46(7).

The scope of C–F bond activation was then expanded to more activated difluorobenzene substrates (Scheme 4). Under otherwise identical conditions, the reaction with difluorobenzenes proceeded more rapidly. The reaction with 1,4-difluorobenzene gave the aryl fluoride derivative **6** ultimately after 6 h at 90 °C with the intermediacy of a dialane species like **2**, as evidenced by ^1^H and ^19^F NMR spectroscopy. Compound **6** was isolated in 41% yield by the recrystallization from saturated pentane solution. SC-XRD analysis revealed the molecular structure of **6**, analogous to **3** (Figure 4a). In the ^19^F NMR spectra, the aryl fluorine was observed at −115 ppm and the fluorine connected with aluminum was observed at −170 ppm. Similarly, the reaction with 1,3-difluorobenzene gave exclusively the product of C–F activation in 43% yield (**7**).

Unlike the reactions with 1,4- and 1,3-difluorobenzene, treating a benzene suspension of dialumene **1** with 1,2-difluorobenzene led to a mixture of **8** and **9** in a 2:1 ratio (Scheme 4). In the ^19^F NMR spectra, three signals were observed at −92, −168 ppm (**8**) and −159 ppm (**9**). However, our attempts to purify **8** and **9** failed due to their similar solubility. Fortunately, the molecular structure of **9** was unambiguously determined by SC-XRD analysis, which showed the second insertion of aluminum moiety into the C–F bond (Figure 4b). The Al1–Al2 distance of 3.772 Å was obviously longer than the typical Al–Al single bond (2.55–2.70 Å), indicating no interaction between the two aluminum centers. This difference in reactivity could be attributed to the activating effect of the fluorine atom on the neighboring C–F bond, which weakens the C–F bonds to allow for double alumination.^30^

**Scheme 5:**
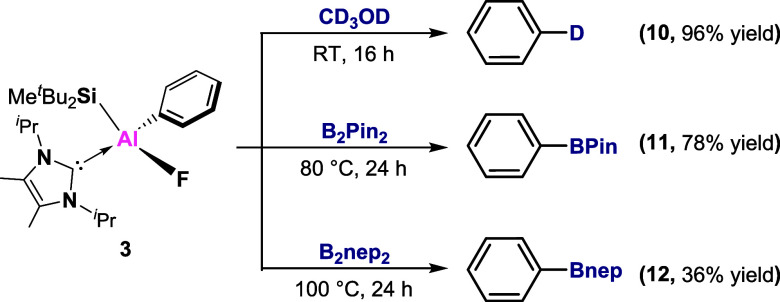
Functionalization of Aryl-Aluminum Compound **3**

Next, we turned to explore the utility of the resulting aryl-aluminum compounds (Scheme 5). Although the generation of aryl-containing main group complexes has been shown in previous studies, the functionalization of these compounds is still underdeveloped.^13,19,20^ The newly formed **3** acts as a nucleophilic reagent, which can transfer the aryl group from aluminum to the electrophiles. Treating **3** with CD_3_OD cleanly formed the monodeuterated benzene **10**. Reactions with B_2_Pin_2_ and B_2_nep_2_ resulted in the formation of borylated compounds **11** and **12** that are synthetically useful building blocks owing to the multiple opportunities for further derivatization.

## Conclusions

In summary, we report a dialumene-mediated activation of monofluorobenzene with exclusive selectivity toward C–F bond activation, which represents an unprecedented example of transition-metal-free fluorobenzene activation and functionalization. The neutral dialumene demonstrates the capability to function as a dimeric or monomeric aluminyl synthon, allowing for the synthesis of the formal oxidative addition products at either double or single aluminum centers. Mechanistically, this reaction starts with the oxidative addition of C–F bond to the Al=Al unit to furnish the dialane Al^II^ product, which then undergoes a very rare reductive elimination process to ultimately deliver the thermodynamically most stable monomeric Al^III^ product. The observed unique reactivities of dialumene can be rationalized by its highly reactive nature and the labile ability of the Al–Al bond in the dialane Al^II^ intermediate. A “masked” alumylene was synthesized for the first time, providing indirect evidence for the proposed reductive elimination process. Additionally, reactivity studies with electrophiles demonstrated that the aryl-aluminum species could serve as a C_6_H_5_^–^ source, giving access to various useful aryl-containing organic compounds.

## Supporting Information Available

Experimental details including synthetic, spectroscopic, crystallographic, and computational data; Cartesian coordinates; crystallographic data (Table S1); key distances (Å) of experimental and DFT-optimized structures of compound **2** (Table S2); NMR spectra of **2**, **3**, **5**, **6**, **7**, **8**, and **9** (Figures S1–S18); kinetic experiments of the reaction from **2** to **3** (Figure S19); dissociation energies of complexes AlF and AlH (kcal/mol) (Figure S20); Gibbs energy profiles (Figures S21–S26) (PDF)
