# Supplementary material for: Dialumene as a Dimeric or Monomeric Al Synthon for C–F Activation in Monofluorobenzene
Source: J Am Chem Soc. 2024 Aug 6;146(33):23591–7. doi: 10.1021/jacs.4c08171 (PMC11345846; doi:10.1021/jacs.4c08171)
Supplement: Supplementary file 1 — ja4c08171_si_001.pdf [file ja4c08171_si_001.pdf]

Supporting Information

Dialumene as a Dimeric or Monomeric Al Synthon for C–F Activation in Monofluorobenzene

Xufang Liu,‡ Shicheng Dong,‡ Jun Zhu* and Shigeyoshi Inoue*

Abstract: The activation of C–F bonds has long been regarded as the subject of research in organometallic chemistry, given their synthetic relevance and the fact that fluorine is the most abundant halogen in the Earth's crust. However, C–F bond activation remains a largely unsolved challenge due to the high bond dissociation energies, which was historically dominated by transition metal complexes. Main group elements that can cleave unactivated monofluorobenzene are still quite rare and restricted to s-block complexes with a biphilic nature. Herein, we demonstrate an Al-mediated activation of monofluorobenzene using a neutral dialumene, allowing for the synthesis of the formal oxidative addition products at either double or single aluminum centers. This neutral dialumene system introduces a novel methodology for C–F bond activation based on formal oxidative addition and reductive elimination processes around the two aluminum centers, as demonstrated by combined experimental and computational studies. A “masked” alumylene was unprecedentedly synthesized to prove the proposed reductive elimination pathway. Furthermore, the synthetic utility is highlighted by the functionalization of the resulting aryl-aluminum compounds.

Contents

1. Experimental Procedures 3

1.1 General Methods and Instrumentation 3

1.2 Synthesis and Characterization 4

1.2.1 Synthesis of 2 4

1.2.2 Synthesis of 3 6

1.2.3 Synthesis of 5 9

1.2.4 Synthesis of 6 12

1.2.5 Synthesis of 7 14

1.2.6 Synthesis of 8 and 9 17

1.2.7 Functionalization of compound 3 20

1.3 Kinetic Experiments 21

2. Single Crystal X-Ray Structure Determination 22

3. Computational Details 25

3.1 Computational Methods 25

3.2 Dissociation energies of complexes AlF and AlH 26

3.3 Gibbs energy profiles for the overall process 27

3.4 Oxidation addition of 2 with fluorobenzene to form 3 29

3.5 Formation of 3 via sequential migration reaction and oxidation addition 30

3.6 Gibbs energy profiles for C-F vs. C-H bond activation 30

3.7 Gibbs energy profiles for the reaction from 5 to 3 31

4. Appendix 32

5. References 99

1. Experimental Procedures

1.1 General Methods and Instrumentation

All experiments and manipulations were carried out under argon atmosphere using standard Schlenk or glovebox techniques. The glassware was heat-dried under vacuum prior to use. All glass junctions were coated with PTFE-based grease Merkel Triboflon III. For stirring, PTFE-coated magnetic stirrer bars were used or glass-coated ones if stated. Liquid phases were transferred using standard PE/PP syringes equipped with stainless steel cannula or directly canted from vessel to vessel if not stated otherwise. Solvents were dried by standard methods (withdrawal from MBraun Solvent Purification System and storage over molecular sieves (3 Å), or distilled from sodium/ benzophenone or CaH_2 under argon atmosphere and degassed via freeze-pump-thaw cycling). All chemicals were purchased from commercial suppliers and used as received if not stated otherwise. Deuterated solvents C_6D_6 and Tol-D_8 were obtained from Deutero Deutschland GmbH and were dried over 3 Å molecular sieves. All NMR samples were prepared under argon in J. Young PTFE tubes. NMR spectra were recorded on a Bruker AV400US, DRX400, AVHD300 and AV500cr at ambient temperature (300 K) if not stated otherwise. ^{1}H and ^{13}C NMR spectra were calibrated against the residual proton and natural abundance carbon resonances of the respective deuterated solvent as internal standard. Liquid Injection Field Desorption Ionization Mass Spectrometry (LIFDI-MS) was measured directly from an inert atmosphere glovebox with a Thermo Fisher Scientific Exactive Plus Orbitrap equipped with an ion source from Linden CMS.

1.2 Synthesis and Characterization

1.2.1 Synthesis of 2

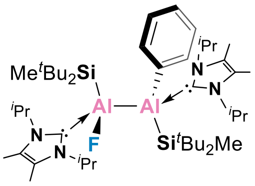

A mixture of dialumene **1** (30 mg, 0.041 mmol) and excess fluorobenzene was stirred in benzene (1 mL) at 65 °C. The dark purple color of the solution vanished gradually and was replaced by a light yellow color after 1.5 days. All volatiles were evaporated at room temperature, the residue was extracted with pentane (3 × 0.5 mL). The pentane solution underwent slow evaporation at room temperature to yield dialane **2** (18.3 mg, 54%) as colorless crystals.

^{1}H NMR (400 MHz, C_6D_6): δ [ppm] 8.58 (d, *J* = 5.4 Hz, 2H, *o*-C*H*- Ph), 7.43 (t, *J* = 5.4 Hz, 2H, *m*-C*H*-Ph), 7.25 (t, *J* = 5.4 Hz, 1H, *p*-C*H*-Ph), 6.39 (sept, *J* = 5.4 Hz, 1H, C*H*(CH_3)$_2$), 5.38 – 5.53 (m, 1H, C*H*(CH_3)$_2$), 5.23 – 5.38 (m, 1H, C*H*(CH_3)$_2$), 4.15 – 4.34 (m, 1H, C*H*(CH_3)$_2$), 1.89 (d, *J* = 4.8 Hz, 3H, CH(CH_3)$_2$), 1.72 (s, 3H, *CH$_3$* NHC), 1.62 (s, 3H, *CH$_3$* NHC), 1.55 (s, 3H, *CH$_3$* NHC), 1.48 – 1.53 (m, 9H, *CH$_3$* NHC, overlapping with CH(CH_3)$_2$), 1.40 (s, 9H, C(CH_3)$_3$), 1.32 (s, 9H, C(CH_3)$_3$), 1.25 – 1.30 (m, 15H, C(CH_3)$_3$, overlapping with CH(CH_3)$_2$), 1.21 (s, 9H, C(CH_3)$_3$), 1.01 (d, *J* = 5.4 Hz, 3H, CH(CH_3)$_2$), 0.71 (d, *J* = 4.8 Hz, 3H, CH(CH_3)$_2$), 0.60 (s, 3H, SiCH_3), 0.56 (s, 3H, SiCH_3), 0.54 (d, *J* = 5.1 Hz, 3H, CH(CH_3)$_2$).

^{13}C{^{1}H} NMR (101 MHz, C_6D_6): δ [ppm] 142.07 (Ar*C*), 126.82 (Ar*C*), 126.60 (Ar*C*), 126.50 (*C*=*C* NHC), 125.82 (Ar*C*), 125.40 (*C*=*C* NHC), 52.48 (*C*H(CH_3)$_2$), 50.75 (*C*H(CH_3)$_2$), 49.32 (*C*H(CH_3)$_2$), 49.09 (*C*H(CH_3)$_2$), 31.96 (C(*C*H$_3$)$_3$), 31.94 (C(*C*H$_3$)$_3$), 31.76 (C(*C*H$_3$)$_3$), 31.43 (C(*C*H$_3$)$_3$), 23.12, 22.89, 22.62, 22.35, 22.29, 22.21, 22.18, 21.54 (peaks between 21.0 and 23.5 are assigned as Si*C*(CH_3)$_3$ and CH(*C*H$_3$)$_2$), 10.45 (*C*H$_3$ NHC), 10.15 (*C*H$_3$ NHC), -2.10 (Si*C*H$_3$), -3.52 (d, *J* = 5.5 Hz, Si*C*H$_3$).

^{19}F{^{1}H} NMR (377 MHz, C_6D_6): δ [ppm] -167.37 (Al-*F*).

LIFDI-MS: $C_{46}H_{87}Al_2FN_4Si_2$, Calcd: 824.6084; Found: 824.6079.

No signal was found for aluminum-bonded silicon and carbene carbon atom in the ^{29}Si{^{1}H}, ^{13}C{^{1}H} NMR spectrum respectively, due to the quadrupolar momentum of the ^{27}Al nucleus.

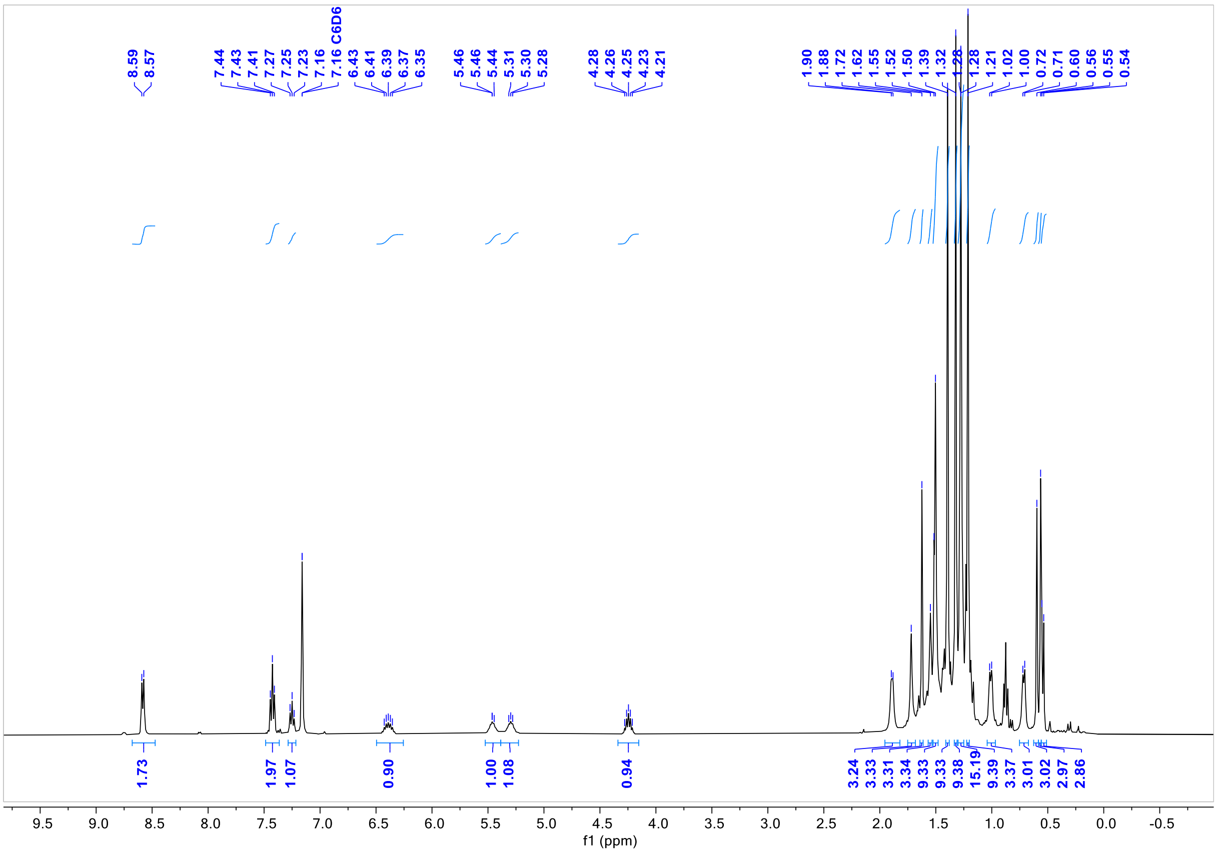

Figure S1. ^{1}H NMR spectrum of **2** in C_6D_6 at 300K.

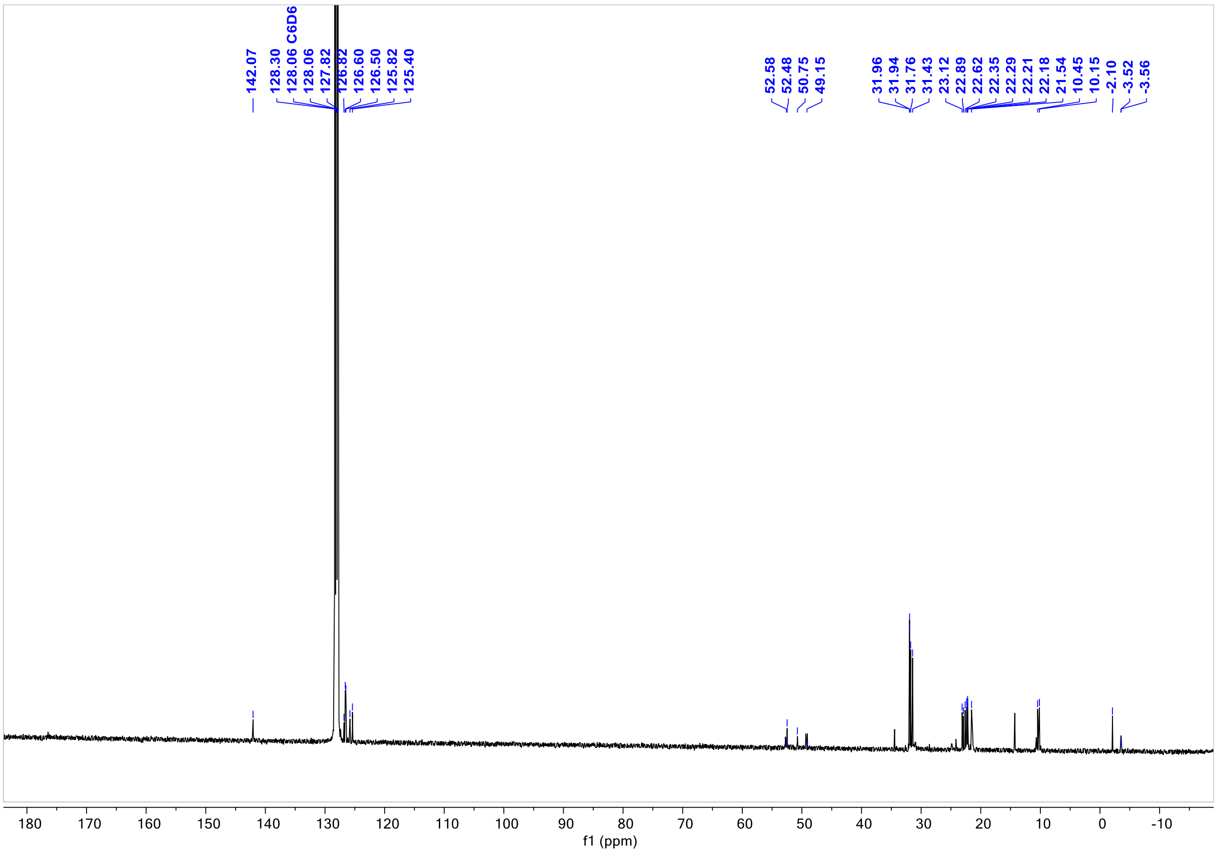

Figure S2. $^{13}C\{^{1}H\}$ NMR spectrum of **2** in C_6D_6 at 300K.

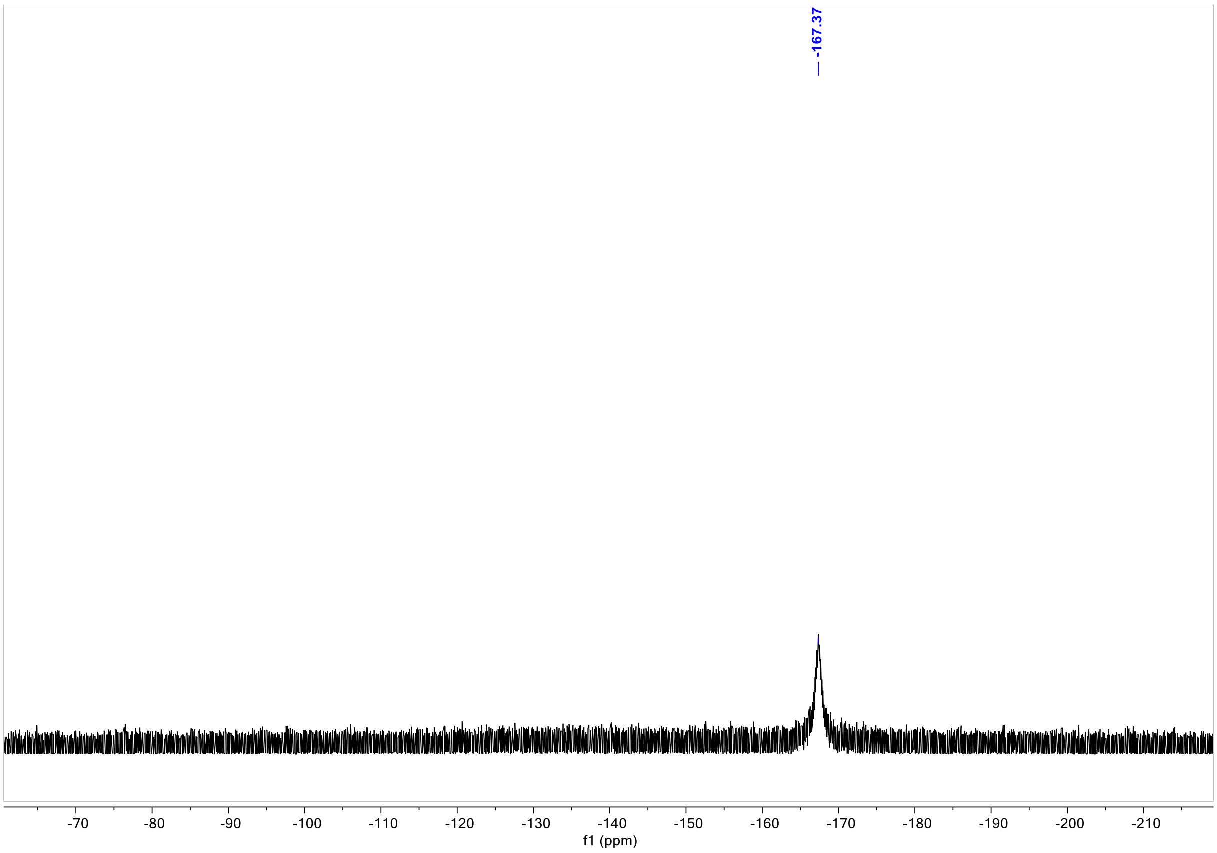

Figure S3. $^{19}F\{^{1}H\}$ NMR spectrum of **2** in C_6D_6 at 300K.

1.2.2 Synthesis of 3

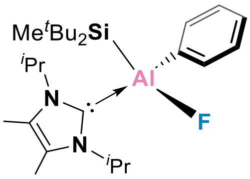

Method A: A mixture of dialumene **1** (30 mg, 0.041 mmol) and excess fluorobenzene was stirred in benzene (1 mL) at 65 °C for 6 days. The color of the solution changed from dark purple to light yellow. All volatiles were evaporated at room temperature, the residue was extracted with pentane (3 × 0.2 mL). The pentane solution underwent slow evaporation at –30 °C to yield **3** (16.2 mg, 43%) as colorless crystals.

Method B: A benzene (0.5 mL) solution of compound **2** (18 mg, 0.022 mmol) was stirred at 65 °C. After 4 days, **2** was completely consumed by ^{1}H NMR monitoring. All volatiles were evaporated at room temperature, the residue was extracted with pentane (3 × 0.1 mL). The pentane solution underwent slow evaporation at –30 °C to yield **3** (9.7 mg, 96%) as colorless crystals.

^{1}H NMR (400 MHz, C_6D_6): δ [ppm] 8.06 (d, *J* = 5.7 Hz, 2H, *o*-C*H*-Ph), 7.36 (t, *J* = 5.4 Hz, 2H, *m*-C*H*-Ph), 7.25 (t, *J* = 5.4 Hz, 1H, *p*-C*H*-Ph), 5.28 – 5.46 (m, 2H, C*H*(CH_3)$_2$), 1.41 (s, 9H, C(CH_3)$_3$), 1.40 (s, 6H, CH_3 NHC), 1.28 (s, 9H, C(CH_3)$_3$), 1.19 (d, *J* = 5.1 Hz, 6H, CH(CH_3)$_2$), 0.83 (d, *J* = 5.4 Hz, 6H, CH(CH_3)$_2$), 0.48 (s, 3H, SiCH_3).

$^{13}C\{^{1}H\}$ NMR (101 MHz, C_6D_6): δ [ppm] 137.71 (Ar*C*), 126.97 (Ar*C*), 126.62 (Ar*C*), 125.75 (*C*=*C* NHC), 51.52 (*C*H(CH_3)$_2$), 51.44 (*C*H(CH_3)$_2$), 30.66 (C(*C*H_3)$_3$), 30.53 (C(*C*H_3)$_3$), 21.82 (CH(*C*H_3)$_2$), 21.08 (Si*C*(CH_3)$_3$), 20.97 (CH(*C*H_3)$_2$), 20.75 (Si*C*(CH_3)$_3$), 9.65 (*C*H_3 NHC), -5.22 (d, *J* = 4.0 Hz, Si*C*H_3).

$^{19}F\{^{1}H\}$ NMR (377 MHz, C_6D_6): δ [ppm] -170.09 (Al-*F*).

LIFDI-MS: $C_{26}H_{46}AlFN_2Si$, Calcd: 460.3230; Found: 460.3224.

No signal was found for aluminum-bonded silicon and carbene carbon atom in the $^{29}Si\{^{1}H\}$, $^{13}C\{^{1}H\}$ NMR spectrum respectively, due to the quadrupolar momentum of the ^{27}Al nucleus.

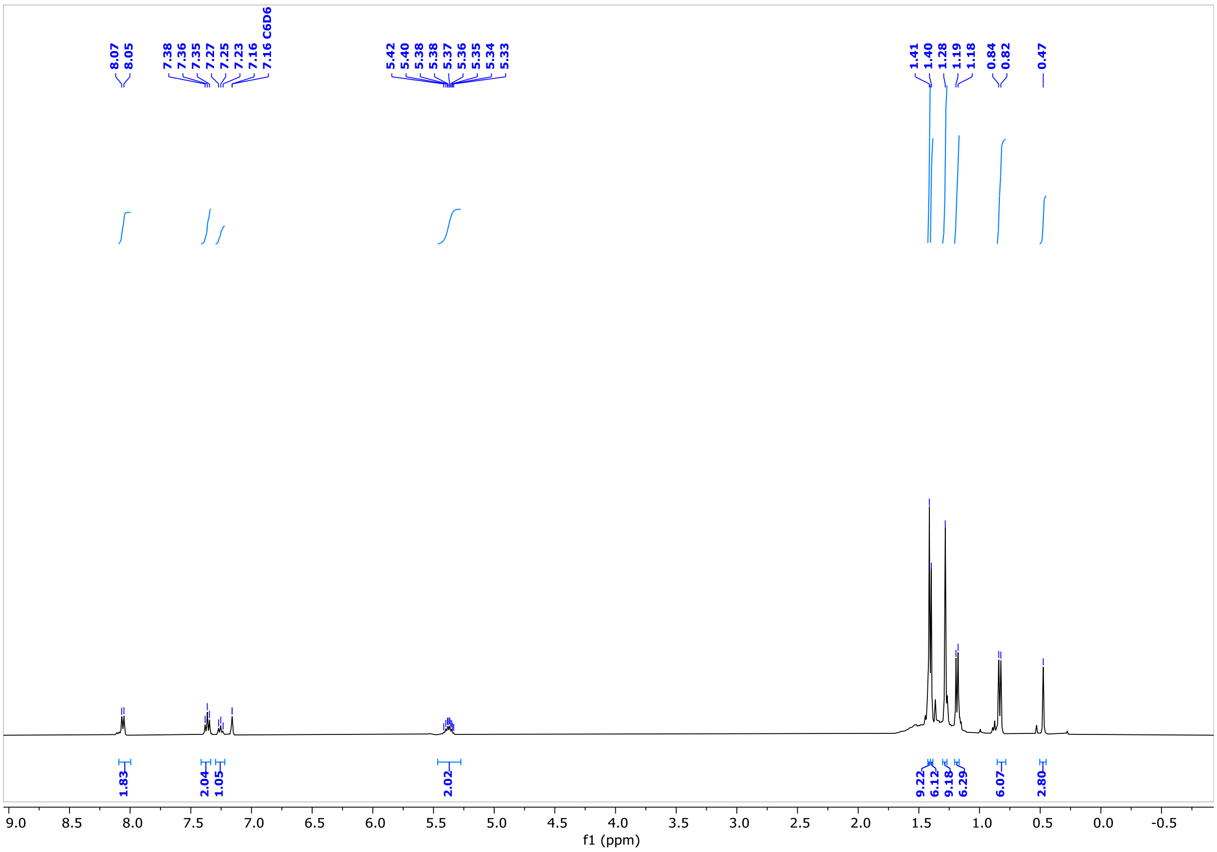

Figure S4. ^{1}H NMR spectrum of **3** in C_6D_6 at 300K.

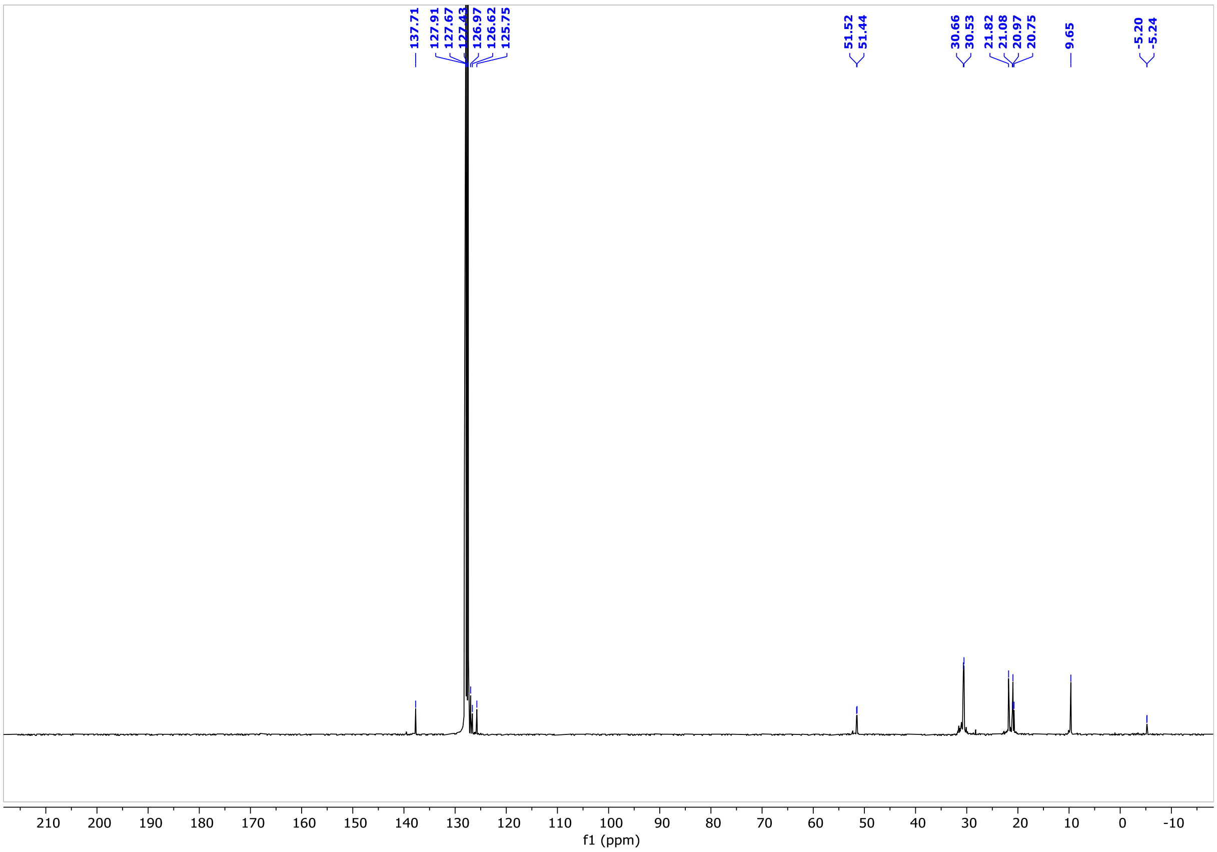

Figure S5. $^{13}C\{^1H\}$ NMR spectrum of **3** in C_6D_6 at 300K.

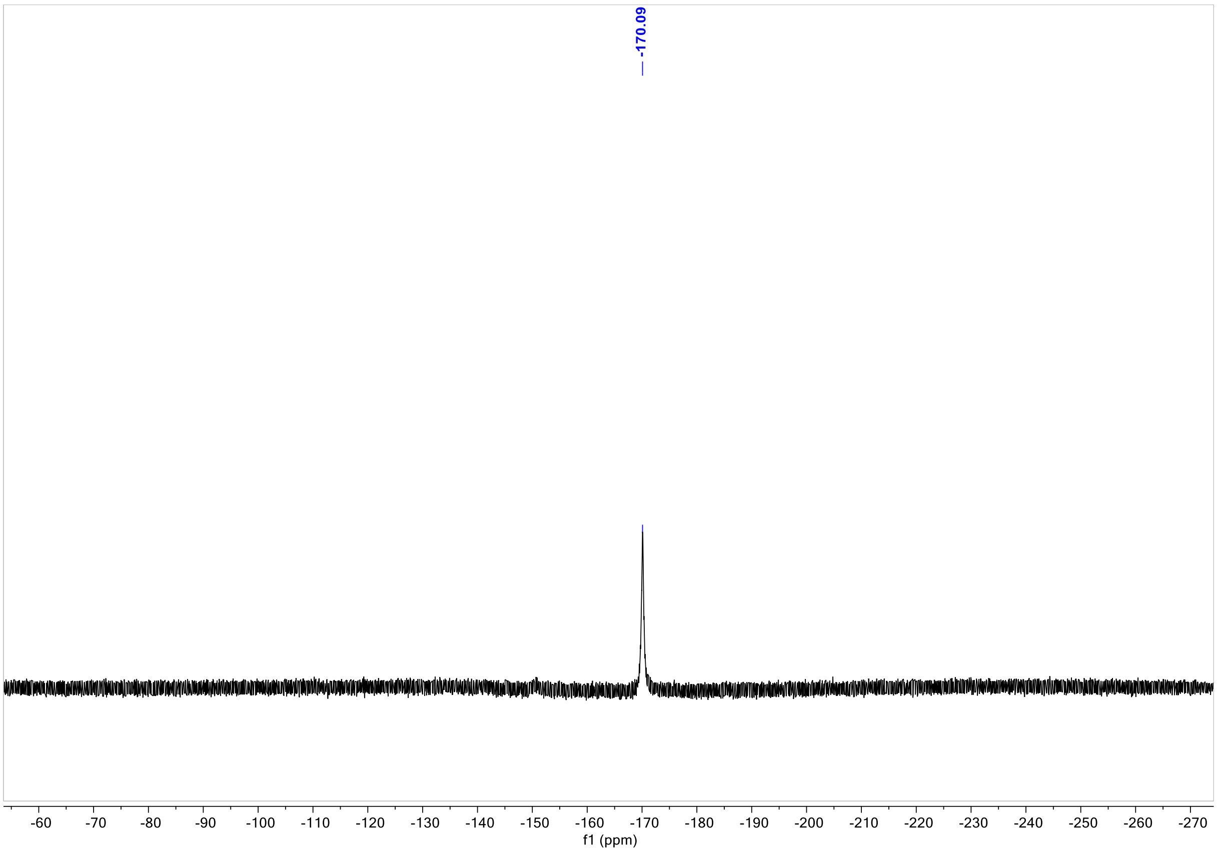

Figure S6. $^{19}F\{^1H\}$ NMR spectrum of **3** in C_6D_6 at 300K.

1.2.3 Synthesis of 5

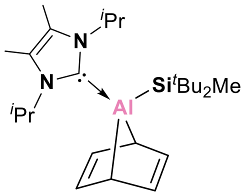

Compound **5** was independently separated from the preparation of dialumene **1**. 7 mL dry benzene were added to a flask containing $^tBu_2MeSi(I^iPr)AlI_2$ (150 mg, 0.24 mmol) and KC_8 (98 mg, 0.72 mmol) at room temperature with vigorous stirring. Gradually the solution turned dark purple and the stirring was continued for 24h at RT. The deep purple solution was filtered and the solid residue was extracted with benzene (3 × 10 mL), until the supernatant become colorless. Afterwards, the volatiles were dried under vacuum and the resulting residue was extracted with pentane (3 × 2 mL). The pentane solution was concentrated and stored at –30 °C to yield **5** (3.2 mg, 3%) as pale yellow crystals.

^{1}H NMR (400 MHz, C_6D_6): δ [ppm] 6.93 – 6.81 (m, 2H, *H*C=C*H*), 6.45 – 6.30 (m, 2H, *H*C=C*H*), 4.90 (hept, *J* = 7.1 Hz, 2H, C*H*(CH_3)$_2$), 2.90 – 2.82 (m, 2H, Al-C*H*), 1.42 (s, 6H, CH_3 NHC), 1.35 (s, 18H, C(CH_3)$_3$), 1.17 (d, *J* = 6.8 Hz, 6H, CH(CH_3)$_2$), 1.09 (d, *J* = 7.1 Hz, 6H, CH(CH_3)$_2$), -0.02 (s, 3H, SiCH_3).

^{13}C{^{1}H} NMR (101 MHz, C_6D_6): δ [ppm] 128.22, 126.85 (*C*=*C* C_6H_6), 124.58 (*C*=*C* NHC), 51.36 (*C*H(CH_3)$_2$), 30.37 (C(*C*H_3)$_3$), 21.63 (CH(*C*H_3)$_2$), 20.99 (CH(*C*H_3)$_2$), 20.97 (Si*C*(CH_3)$_3$), 9.47 (*C*H_3 NHC), -4.77 (Si*C*H_3).

LIFDI-MS: $C_{26}H_{47}AlN_2Si$, Calcd: 442.3324; Found: 442.3318.

No signal was found for aluminum-bonded silicon and carbene carbon atom in the ^{29}Si{^{1}H}, ^{13}C{^{1}H} NMR spectrum respectively, due to the quadrupolar momentum of the ^{27}Al nucleus.

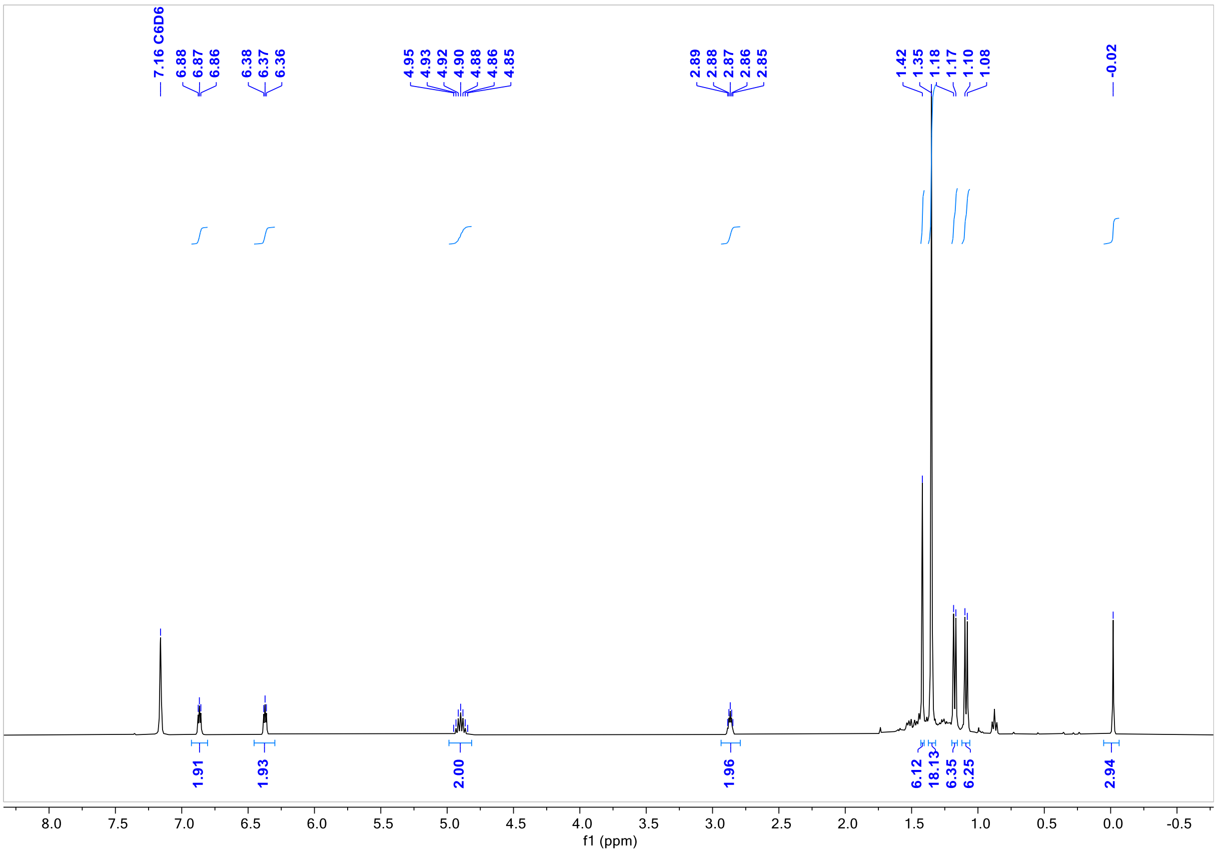

Figure S7. ^{1}H NMR spectrum of **5** in C_6D_6 at 300K.

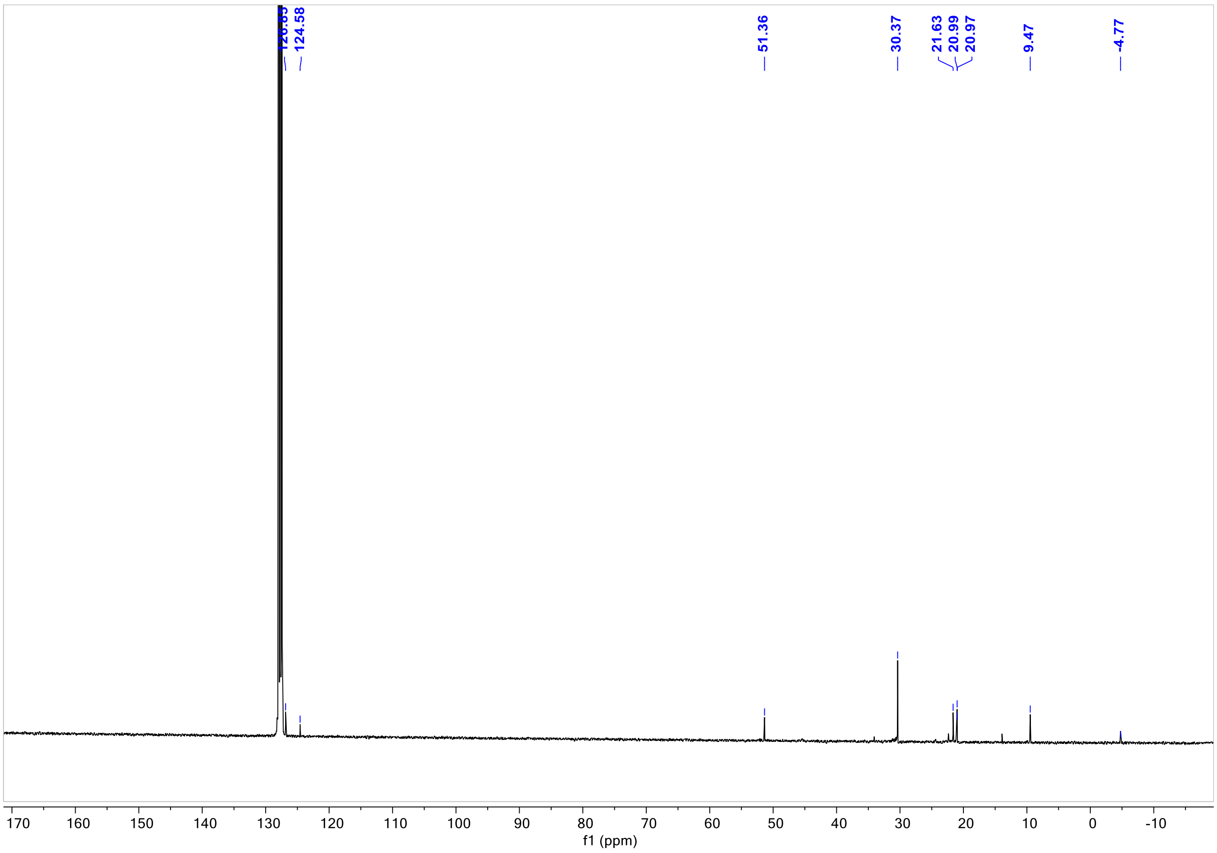

Figure S8. $^{13}C\{^1H\}$ NMR spectrum of **5** in C_6D_6 at 300K.

Demonstrating the properties of **5** as a 'masked' species of **4** would provide indirect evidence for the intermediacy of the highly unstable species **4**. As shown in Figure **S9**, in C_6D_6 solution, the signal corresponding to the C_6H_6 moiety of **5** gradually disappeared, while the signal intensity of free C_6H_6 increased. This indicated the intermolecular exchange of the C_6H_6 moiety of **5** with the C_6D_6 solvent (Figure **S9**).

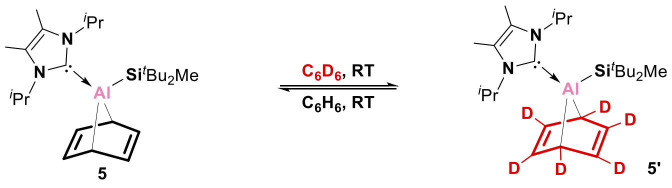

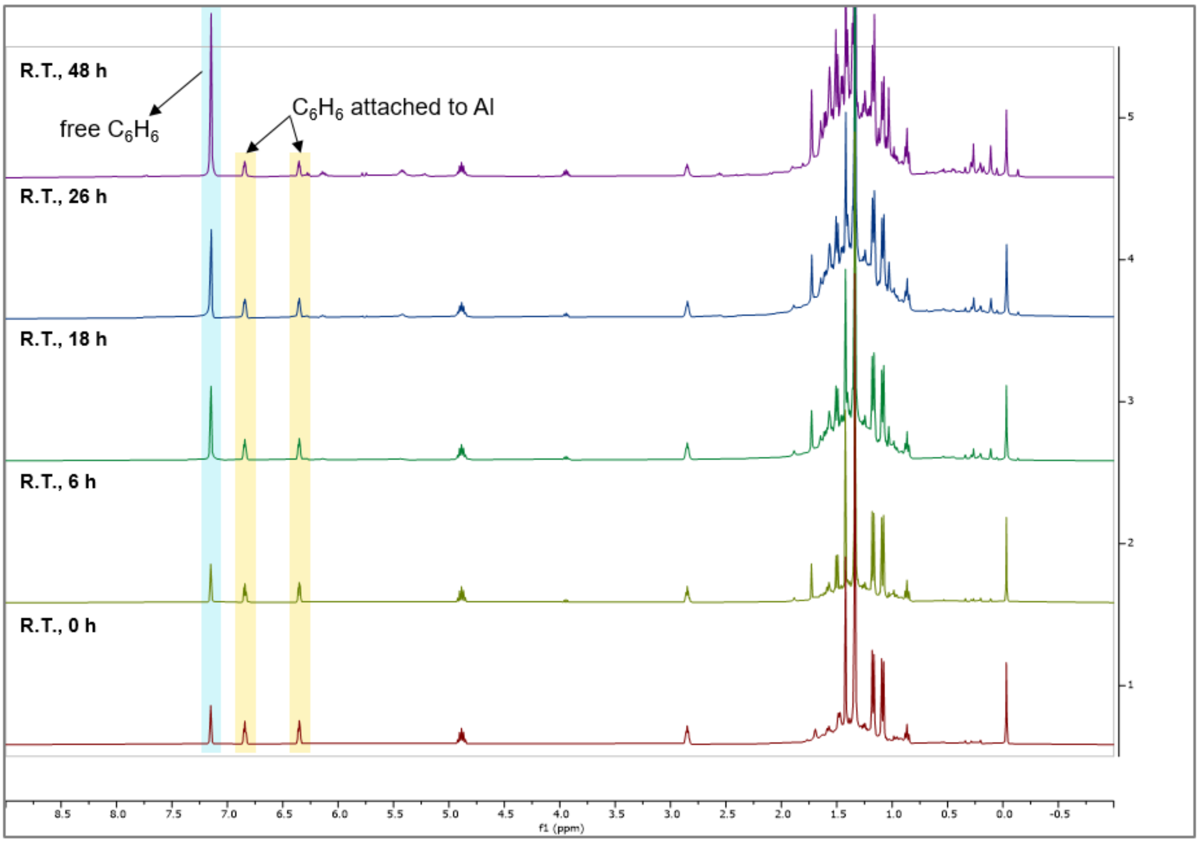

Figure S9. Intermolecular exchange reaction of **5** with C_6D_6.

1.2.4 Synthesis of 6

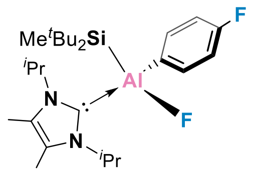

A mixture of dialumene **1** (30 mg, 0.041 mmol) and excess 1,4-difluorobenzene was stirred in benzene (1 mL) at 65 °C for 4 days (or 90 °C, 6 h). The color of the solution changed from dark purple to light yellow. All volatiles were evaporated at room temperature, the residue was extracted with pentane (3 × 0.2 mL). The pentane solution underwent slow evaporation at –30 °C to yield **6** (16.0 mg, 41%) as colorless crystals.

^{1}H NMR (400 MHz, C_6D_6): δ [ppm] 7.92 (t, *J* = 5.4 Hz, 2H, *o*-C*H*-Ar), 7.04 – 7.13 (m, 2H, *m*-C*H*-Ar), 5.22 – 5.38 (m, 2H, C*H*(CH_3)$_2$), 1.40 (s, 9H, C(C*H*$_3$)$_3$), 1.37 (s, 6H, *CH$_3$* NHC), 1.27 (s, 9H, C(C*H*$_3$)$_3$), 1.15 (d, *J* = 5.4 Hz, 6H, CH(C*H*$_3$)$_2$), 0.79 (d, *J* = 5.4 Hz, 6H, CH(C*H*$_3$)$_2$), 0.44 (s, 3H, SiC*H*$_3$).

^{13}C{^{1}H} NMR (101 MHz, C_6D_6): δ [ppm] 163.01 (d, *J* = 245.4 Hz, Ar*C*), 139.13 (d, *J* = 6.1Hz, Ar*C*), 125.71 (*C*=*C* NHC), 113.87 (d, *J* = 17.2 Hz, Ar*C*), 51.49 (*C*H(CH_3)$_2$), 51.41 (*C*H(CH_3)$_2$), 30.58 (C(*C*H$_3$)$_3$), 30.48 (C(*C*H$_3$)$_3$), 21.73 (CH(*C*H$_3$)$_2$), 21.07 (Si*C*(CH_3)$_3$), 20.94 (CH(*C*H$_3$)$_2$), 20.72 (Si*C*(CH_3)$_3$), 9.56 (*C*H$_3$ NHC), -5.30 (d, *J* = 4.0 Hz, Si*C*H$_3$).

^{19}F{^{1}H} NMR (377 MHz, C_6D_6): δ [ppm] -115.35 (tt, *J* = 10.1, 7.0 Hz, Ar-*F*), -169.89 (Al-*F*).

LIFDI-MS: $C_{26}H_{45}AlF_2N_2Si$, Calcd: 478.3135; Found: 478.3130.

No signal was found for aluminum-bonded silicon and carbene carbon atom in the ^{29}Si{^{1}H}, ^{13}C{^{1}H} NMR spectrum respectively, due to the quadrupolar momentum of the ^{27}Al nucleus.

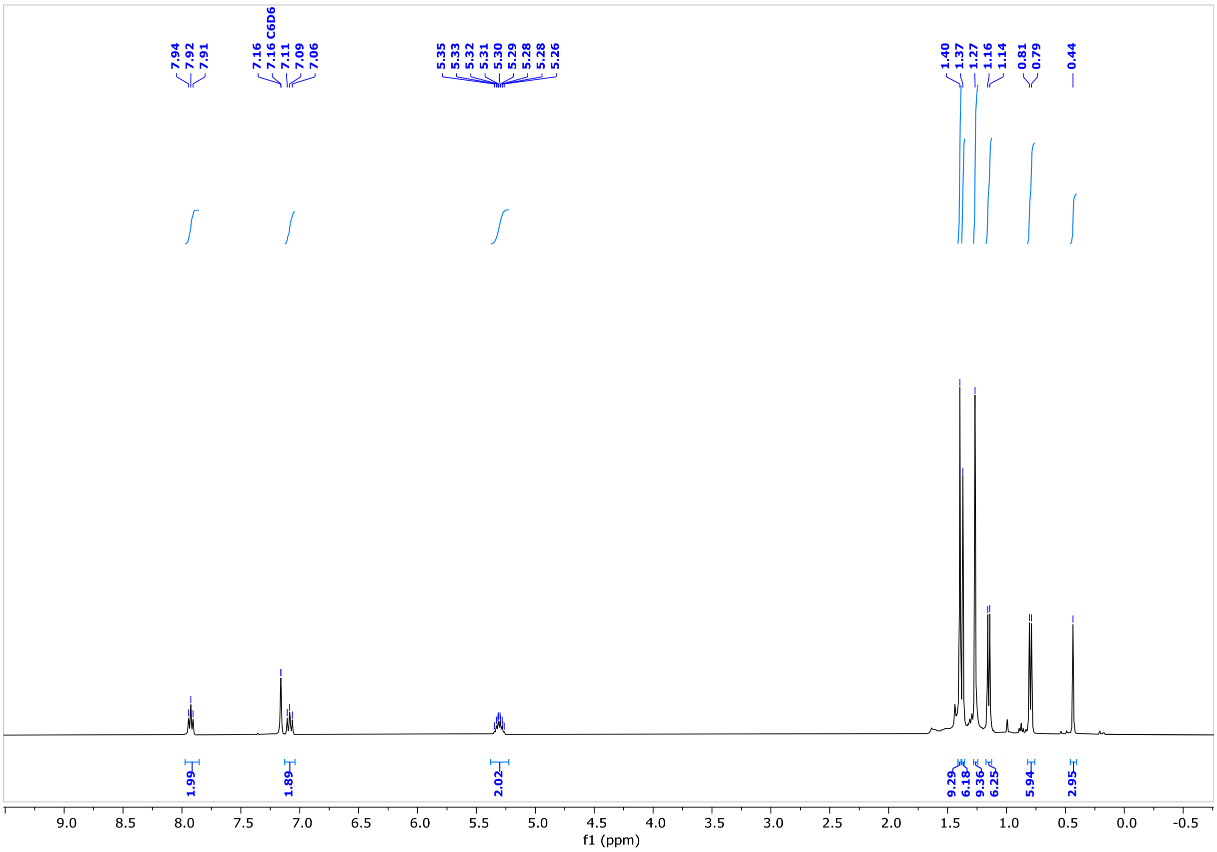

Figure S10. ^{1}H NMR spectrum of **6** in C_6D_6 at 300K.

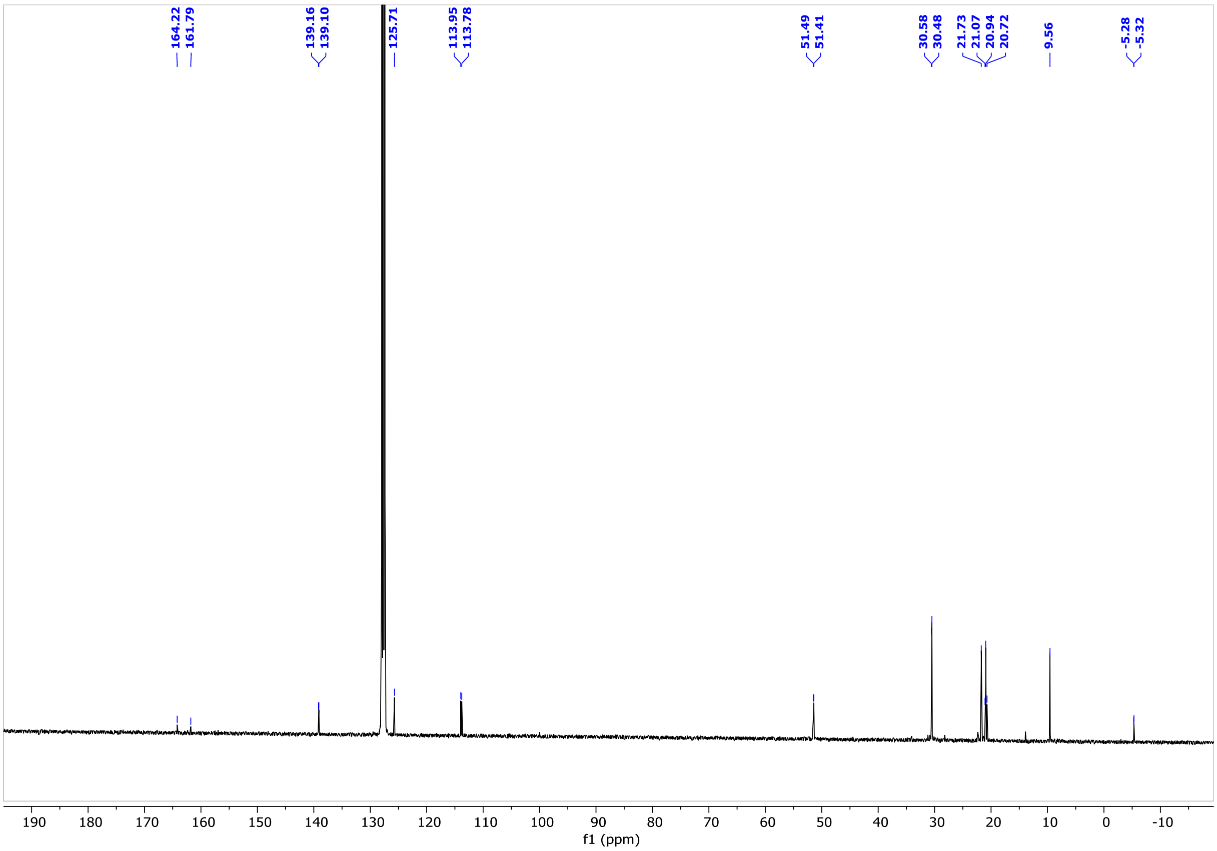

Figure S11. $^{13}C\{^{1}H\}$ NMR spectrum of **6** in C_6D_6 at 300K.

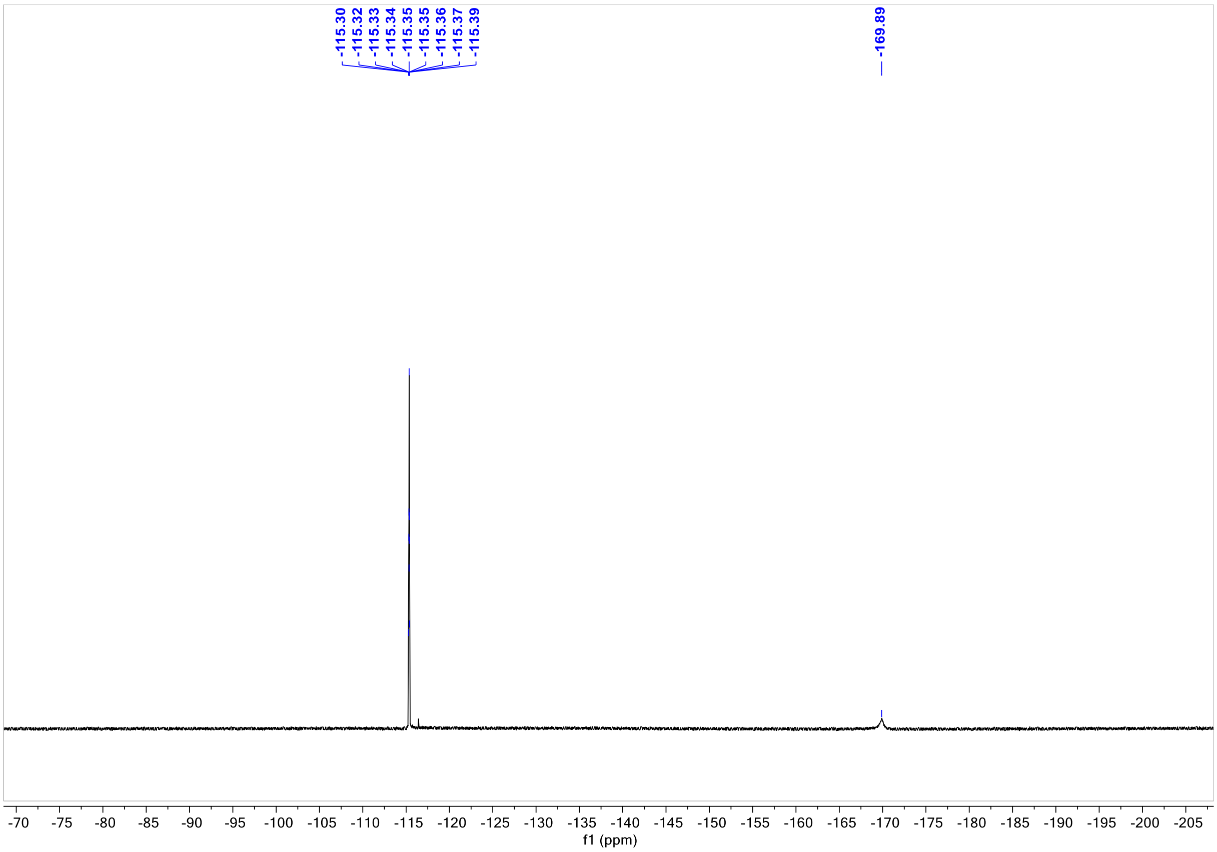

Figure S12. $^{19}F\{^{1}H\}$ NMR spectrum of **6** in C_6D_6 at 300K.

1.2.5 Synthesis of 7

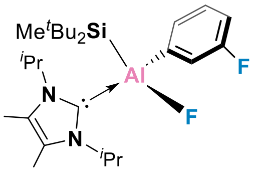

A mixture of dialumene **1** (30 mg, 0.041 mmol) and excess 1,3-difluorobenzene was stirred in benzene (1 mL) at 65 °C for 4 days (or 90 °C, 6 h). The color of the solution changed from dark purple to light yellow. All volatiles were evaporated at room temperature, the residue was extracted with pentane (3 × 0.2 mL). The pentane solution underwent slow evaporation at –30 °C to yield **7** (16.8 mg, 43%) as colorless crystals.

^{1}H NMR (400 MHz, C_6D_6): δ [ppm] 7.85 (dd, *J* = 8.9, 2.8 Hz, 1H, C*H*-Ar), 7.77 (d, *J* = 7.0 Hz, 1H, C*H*-Ar), 7.23 – 7.17 (m, 1H, C*H*-Ar), 6.98 – 6.88 (m, 1H, C*H*-Ar), 5.38 – 5.22 (m, 2H, C*H*(CH_3)$_2$), 1.38 (s, 9H, C(CH_3)$_3$), 1.35 (s, 6H, *CH$_3$* NHC), 1.25 (s, 9H, C(CH_3)$_3$), 1.13 (d, *J* = 7.0 Hz, 6H, CH(CH_3)$_2$), 0.81 (d, *J* = 7.0 Hz, 6H, CH(CH_3)$_2$), 0.43 (s, 3H, SiCH_3).

$^{13}C\{^{1}H\}$ NMR (101 MHz, C_6D_6): δ [ppm] 162.87 (d, *J* = 247.5 Hz, Ar*C*), 133.01 (Ar*C*), 128.58 (d, *J* = 6.1 Hz, Ar*C*), 125.79 (*C*=*C* NHC), 123.32 (d, *J* = 1.9 Hz, Ar*C*), 123.17 (d, *J* = 1.4 Hz, Ar*C*), 113.32 (d, *J* = 20.2 Hz, Ar*C*), 51.53 (*C*H(CH_3)$_2$), 51.45 (*C*H(CH_3)$_2$), 30.54 (C(*C*H_3)$_3$), 30.46 (C(*C*H_3)$_3$), 21.70 (CH(*C*H_3)$_2$), 21.05 (Si*C*(CH_3)$_3$), 20.93 (CH(*C*H_3)$_2$), 20.70 (Si*C*(CH_3)$_3$), 9.56 (*C*H_3 NHC), -5.33 (d, *J* = 4.0 Hz, Si*C*H_3).

$^{19}F\{^1H\}$ NMR (377 MHz, C_6D_6): δ [ppm] -115.29 (dd, *J* = 7.5 Hz, Ar-*F*), -170.03 (Al-*F*).

LIFDI-MS: $C_{26}H_{45}AlF_2N_2Si$, Calcd: 478.3135; Found: 478.3130.

No signal was found for aluminum-bonded silicon and carbene carbon atom in the $^{29}Si\{^1H\}$, $^{13}C\{^1H\}$ NMR spectrum respectively, due to the quadrupolar momentum of the ^{27}Al nucleus.

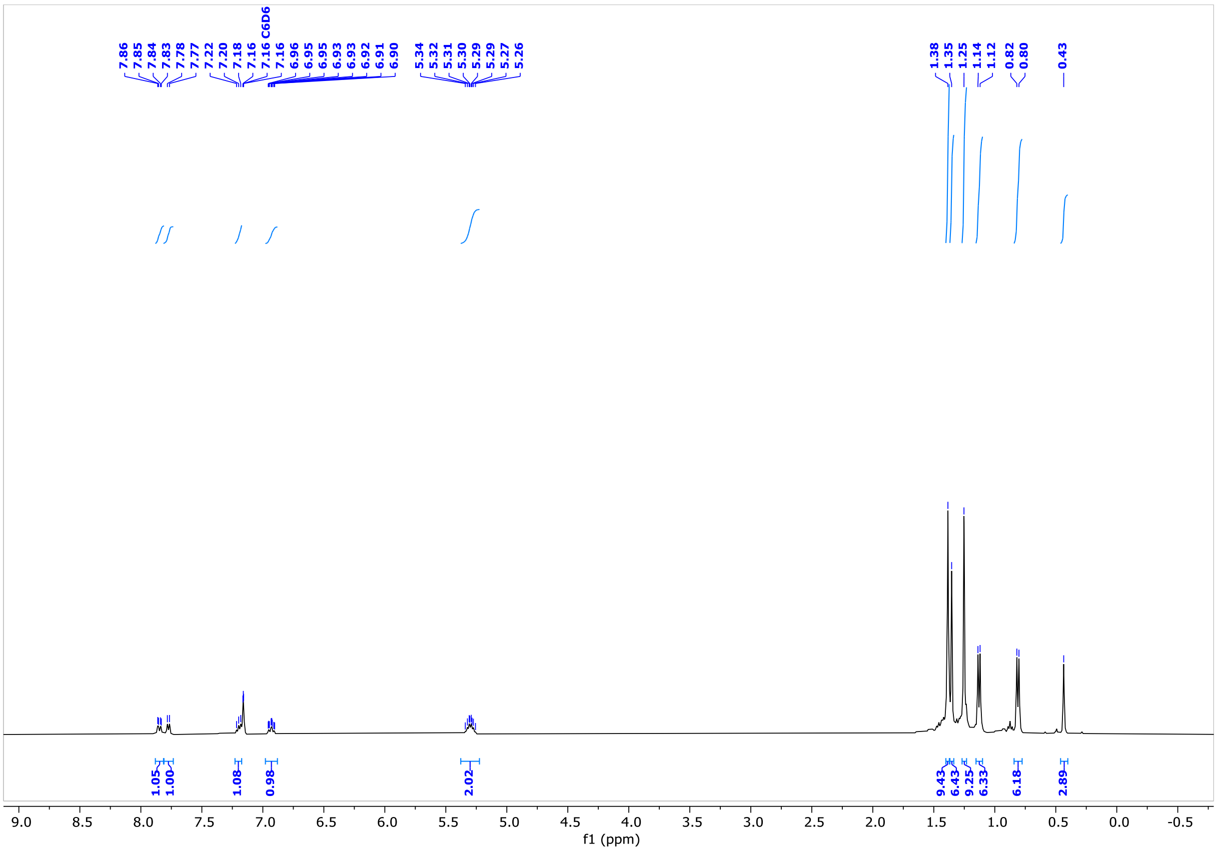

Figure S13. ^1H NMR spectrum of **7** in C_6D_6 at 300K.

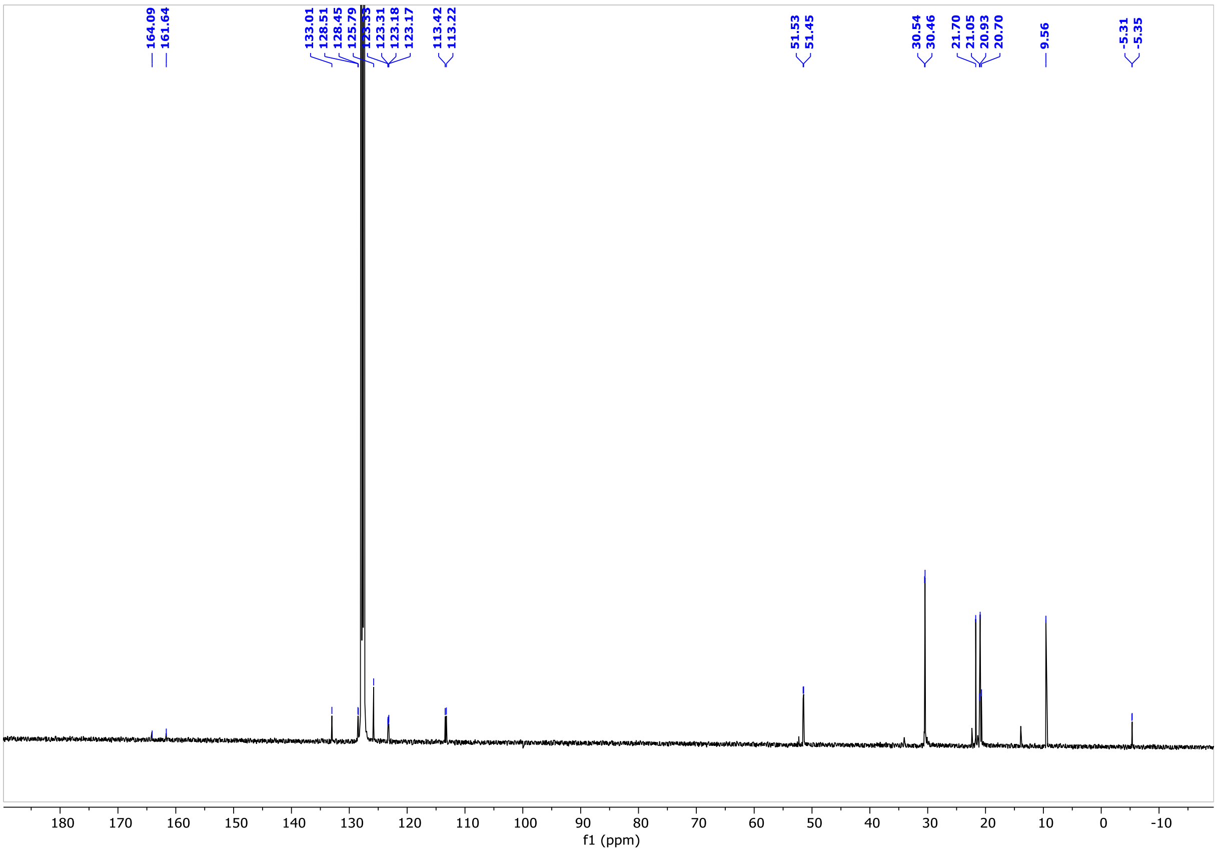

Figure S14. ^{13}C NMR spectrum of **7** in C_6D_6 at 300K.

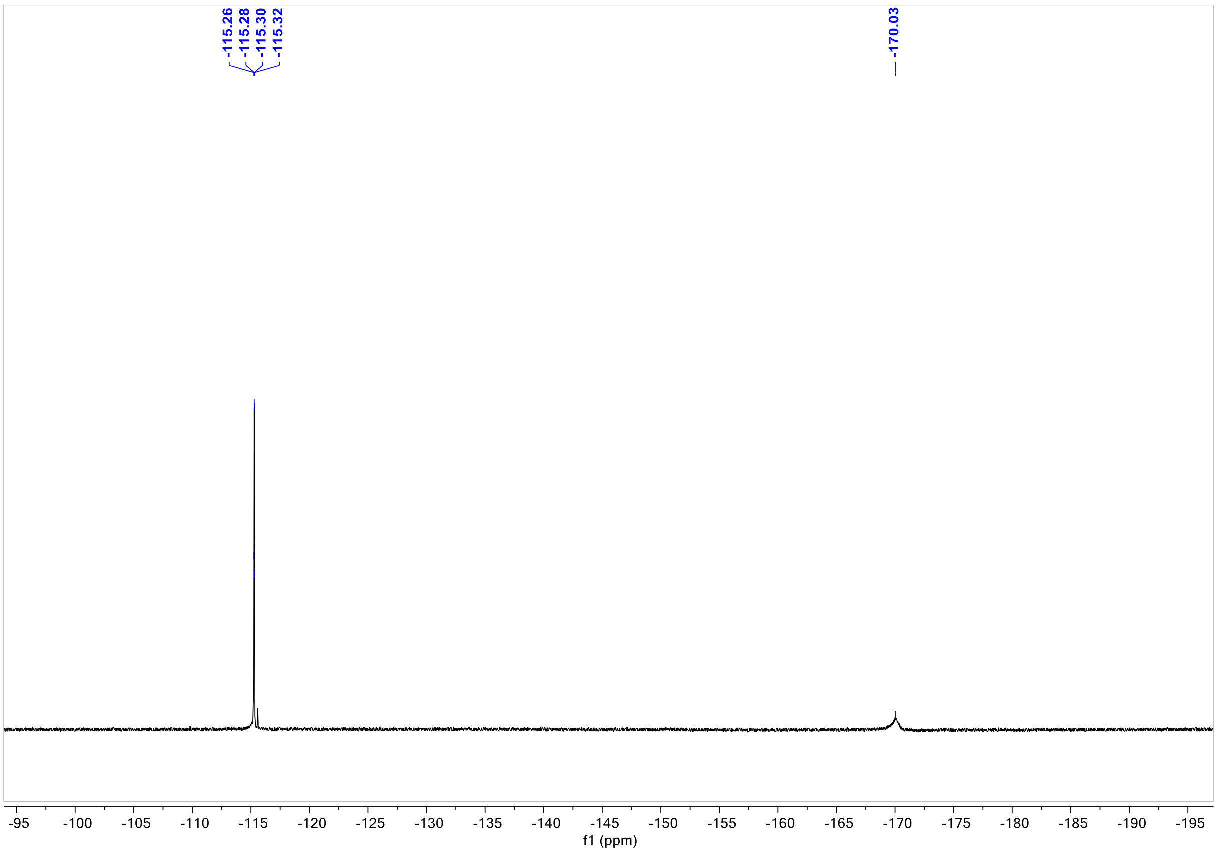

Figure S15. $^{19}F\{^1H\}$ NMR spectrum of **7** in C_6D_6 at 300K.

1.2.6 Synthesis of 8 and 9

A mixture of dialumene **1** (30 mg, 0.041 mmol) and excess 1,2-difluorobenzene was stirred in benzene (1 mL) at 65 °C for 4 days (or 90 °C, 6 h). The color of the solution changed from dark purple to light yellow. All volatiles were evaporated at room temperature, the residue was extracted with pentane (3 × 0.2 mL). The pentane solution underwent slow evaporation at –30 °C to yield a 1 : 1 mixture of **8** and **9** (14.0 mg, 38%) as colorless powder. Attempts to separate **8** and **9** failed due to their similar solubility.

Compound 8:

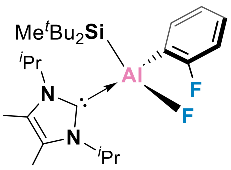

^{1}H NMR (400 MHz, C_6D_6): δ [ppm] 8.07 (t, *J* = 5.7 Hz, 1H, C*H*-Ar), 7.14 – 7.02 (m, 2H, C*H*-Ar), 6.91 (t, *J* = 7.4 Hz, 1H, C*H*-Ar), 5.49 – 5.37 (m, 2H, C*H*(CH$_3$)$_2$), 1.42 (s, 9H, C(C*H*$_3$)$_3$), 1.39 (s, 6H, *CH$_3$* NHC), 1.30 (s, 9H, C(C*H*$_3$)$_3$), 1.19 (d, *J* = 7.2 Hz, 6H, CH(C*H*$_3$)$_2$), 0.93 (d, *J* = 7.0 Hz, 6H, CH(C*H*$_3$)$_2$), 0.49 (s, 3H, SiC*H$_3$*).

^{13}C{^{1}H} NMR (101 MHz, C_6D_6): δ [ppm] 139.30 (Ar*C*), 139.10 (Ar*C*), 129.04 (d, *J* = 8.1 Hz, Ar*C*), 125.77 (*C*=*C* NHC), 123.59 (Ar*C*), 113.40 (d, *J* = 30.3 Hz, Ar*C*), 51.48 (*C*H(CH$_3$)$_2$), 51.40 (*C*H(CH$_3$)$_2$), 30.56 (C(*C*H$_3$)$_3$), 30.45 (C(*C*H$_3$)$_3$), 21.62 (CH(*C*H$_3$)$_2$), 21.23 (Si*C*(CH$_3$)$_3$), 21.03 (CH(*C*H$_3$)$_2$), 20.83 (Si*C*(CH$_3$)$_3$), 9.60 (*C*H$_3$ NHC), -5.78 (Si*C*H$_3$).

^{19}F{^{1}H} NMR (377 MHz, C_6D_6): δ [ppm] -92.59 (Ar-*F*), -165.45 (Al-*F*).

LIFDI-MS: $C_{26}H_{45}AlF_2N_2Si$, Calcd: 478.3135; Found: 459.3156 (**8**-F).

No signal was found for aluminum-bonded silicon and carbene carbon atom in the ^{29}Si{^{1}H}, ^{13}C{^{1}H} NMR spectrum respectively, due to the quadrupolar momentum of the ^{27}Al nucleus.

Compound 9:

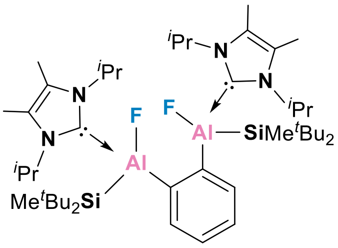

^{1}H NMR (400 MHz, C_6D_6): δ [ppm] 8.29 – 8.21 (m, 2H, C*H*-Ar), 7.46 – 7.40 (m, 2H, C*H*-Ar), 5.61 – 5.50 (m, 4H, C*H*(CH_3)$_2$), 1.55 (s, 12H, *CH$_3$* NHC), 1.48 (d, *J* = 6.9 Hz, 12H, CH(C*H*$_3$)$_2$), 1.26 (s, 18H, C(C*H*$_3$)$_3$), 1.24 (s, 18H, C(C*H*$_3$)$_3$), 1.02 (d, *J* = 6.8 Hz, 12H, CH(C*H*$_3$)$_2$), 0.55 (s, 6H, SiC*H$_3$*).

^{13}C{^{1}H} NMR (101 MHz, C_6D_6): δ [ppm] 137.91 (Ar*C*), 125.37 (*C*=*C* NHC), 124.90 (Ar*C*), 51.40 (*C*H(CH_3)$_2$), 51.33 (*C*H(CH_3)$_2$), 31.05 (C(*C*H$_3$)$_3$), 30.77 (C(*C*H$_3$)$_3$), 21.89, 21.82, 21.78, 21.51, 21.00 (peaks between 21.0 and 22.0 are assigned as Si*C*(CH_3)$_3$ and CH(*C*H$_3$)$_2$), 9.88 (*C*H$_3$ NHC), -4.08 (Si*C*H$_3$).

^{19}F{^{1}H} NMR (377 MHz, C_6D_6): δ [ppm] -159.09 (Al-*F*).

LIFDI-MS: $C_{46}H_{86}Al_2F_2N_4Si_2$, Calcd: 842.5990; Found: 842.5984.

No signal was found for aluminum-bonded silicon and carbene carbon atom in the ^{29}Si{^{1}H}, ^{13}C{^{1}H} NMR spectrum respectively, due to the quadrupolar momentum of the ^{27}Al nucleus.

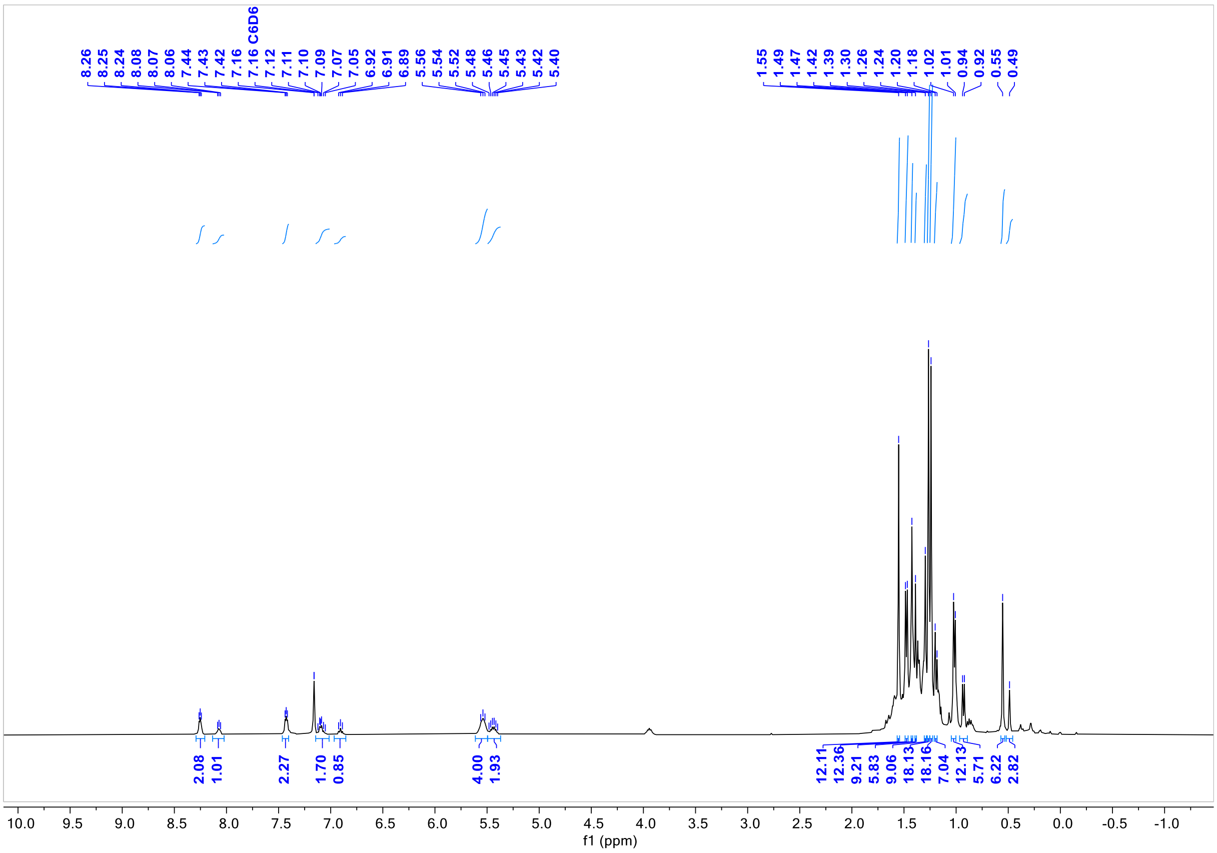

Figure S16. ^{1}H NMR spectrum of a mixture of **8** and **9** in C_6D_6 at 300K.

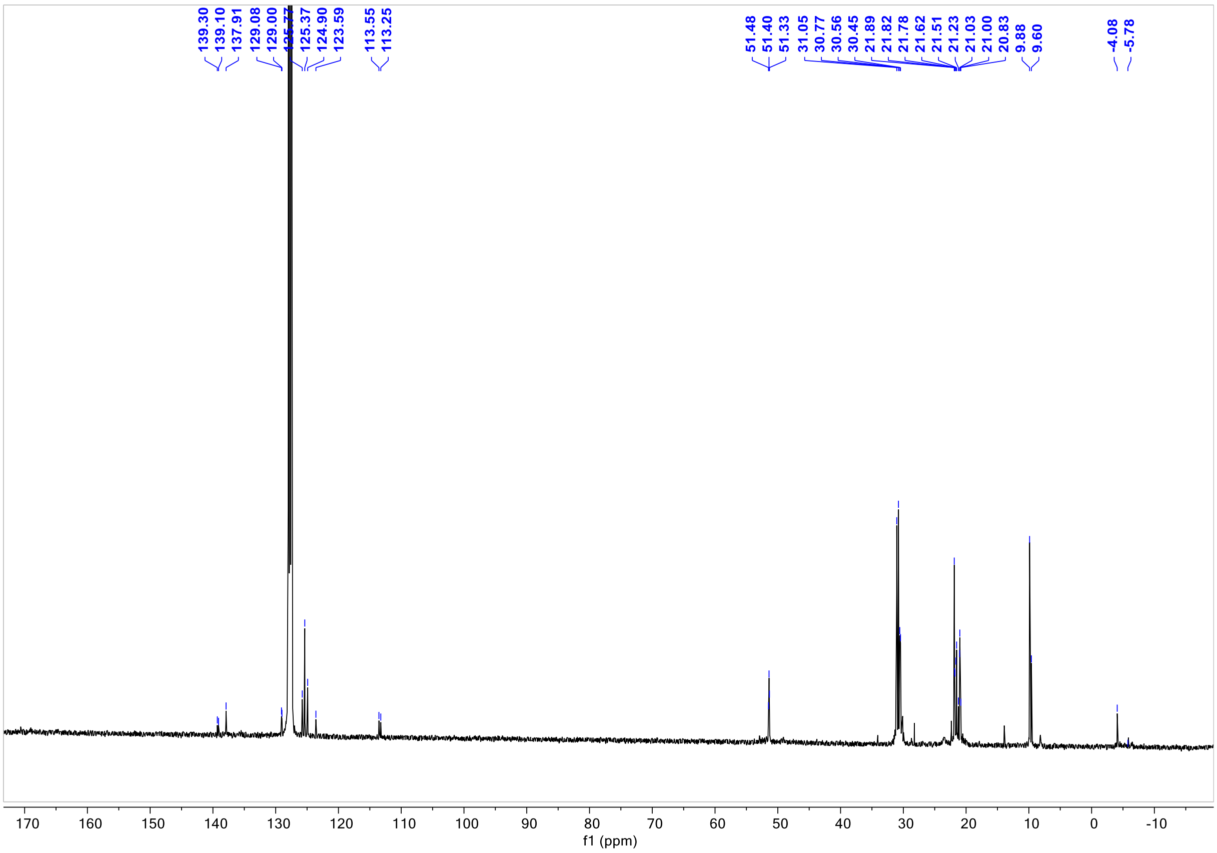

Figure S17. ^{13}C NMR spectrum of a mixture of **8** and **9** in C_6D_6 at 300K.

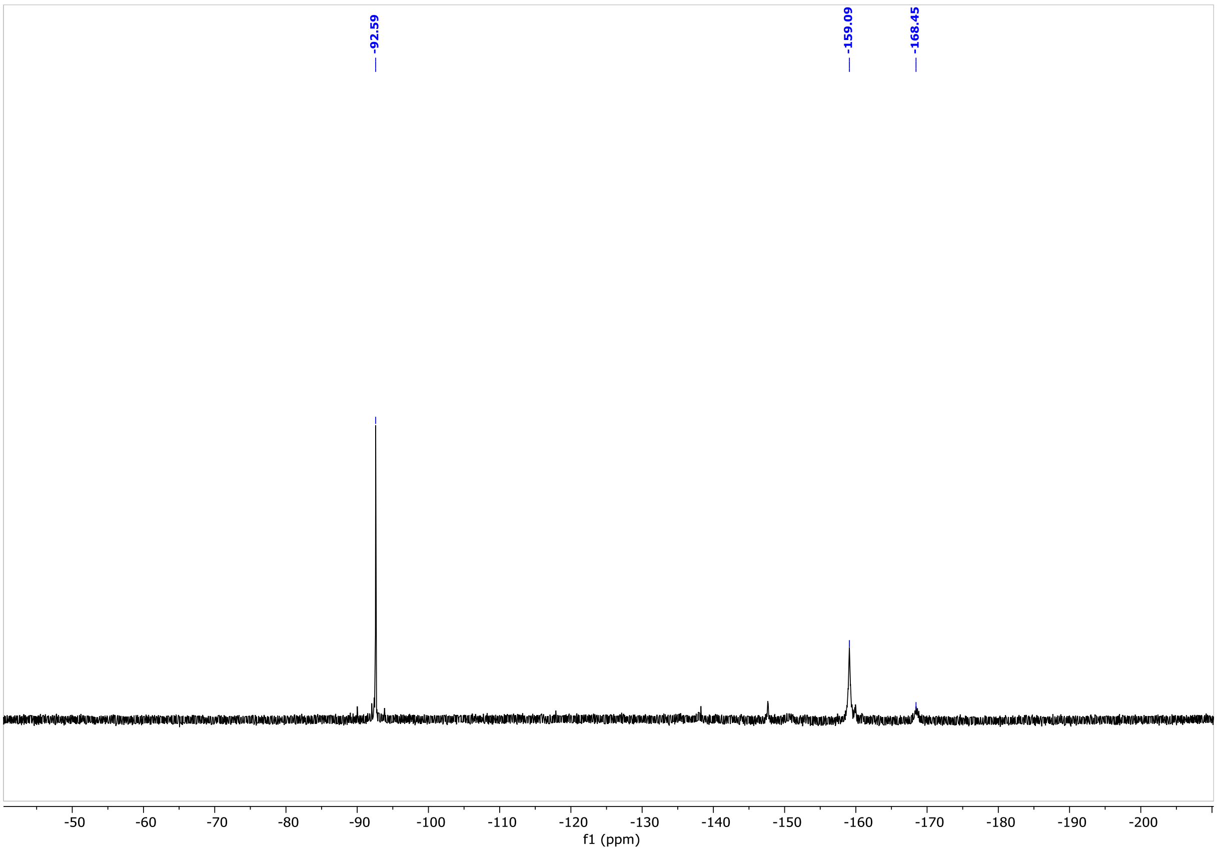

Figure S18. ^{19}F{^{1}H} NMR spectrum of a mixture of **8** and **9** in C_6D_6 at 300K.

1.2.7 Functionalization of compound 3

Preparation of 10:

3 (8 mg, 0.017 mmol) was dissolved in Tol-d_8 (0.3 mL) and added to a J. Young NMR tube with a 1,3,5-trimethoxybenzene internal standard. Then CD_3OD (10 eq.) was added and reacted at room temperature for 16 hours. The formation of **10** was confirmed by a singlet resonance at 7.17 ppm in the ^1H NMR spectra, and the yield (96 %) was determined by the in situ comparison of the new resonance in the ^1H NMR spectrum at 7.17 ppm (5H) to the 1,3,5-trimethoxybenzene internal standard. However, attempts to identify the fate of the aluminum part in **3** failed because the targeted Al–C cleavage often comes with Al–Si cleavage or NHC dissociation.

Preparation of 11:

3 (8 mg, 0.017 mmol) was dissolved in C_6D_6 (0.3 mL) and added to a J. Young NMR tube with a 1,3,5-trimethoxybenzene internal standard. Then pinacolborane (HBpin) (8.6 mg, 0.034 mmol) was added and the reaction was heated to 80 °C for 24 hours. The target product was confirmed by a singlet resonance at 31.5 ppm in the ^{11}B NMR spectra, which is in accordance with the literature[S1]. The yield (78 %) was determined by the in situ comparison of the new doublet resonance in the ^1H NMR spectrum at 8.16 ppm (2H) to the 1,3,5-trimethoxybenzene internal standard. Meanwhile, the concomitant formation of a (NHC)(Si^{t}Bu2Me)F-Al-Bpin species via Al–C cleavage was confirmed by LIFDI-MS ($C_{26}H_{53}AlBFN_2O_2Si$, Calcd: 510.3769; Found: 510.3763).

Preparation of 12:

3 (8 mg, 0.017 mmol) was dissolved in C_6D_6 (0.3 mL) and added to a J. Young NMR tube with a 1,3,5-trimethoxybenzene internal standard. Then bis(neopentyl glycolato)diboron (B_2nep_2) (7.7 mg, 0.034 mmol) was added and the reaction was heated to 100 °C for 24 hours. The reaction mixture was analyzed by ^1H and ^{11}B NMR spectroscopy. The product **12** was confirmed by the observation of new resonance at 28.6 ppm in the ^{11}B NMR spectra, which is in accordance with the literature[S2]. The yield (36 %) was determined by the in situ comparison of the new doublet resonance in the ^1H NMR spectrum at 8.23 ppm (2H) to the 1,3,5-trimethoxybenzene internal standard. However, we couldn’t get any clues regarding on the fate of the aluminum part, possibly due to the much higher temperature required in this case.

1.3 Kinetic Experiments

We carried out the experiments of the reaction from **2** to **3** at different temperatures. The results are presented in the figure below (Figure S**19**). Based on the kinetic profiles of the reactions at different temperatures, we determined the corresponding rate constants (k). By plotting ln(k) vs. -1/T according to the Arrhenius equation, we calculated the activation energy for the rate-determining step to be 30.1 kcal mol^{-1}, which is close to the calculated activation energy.

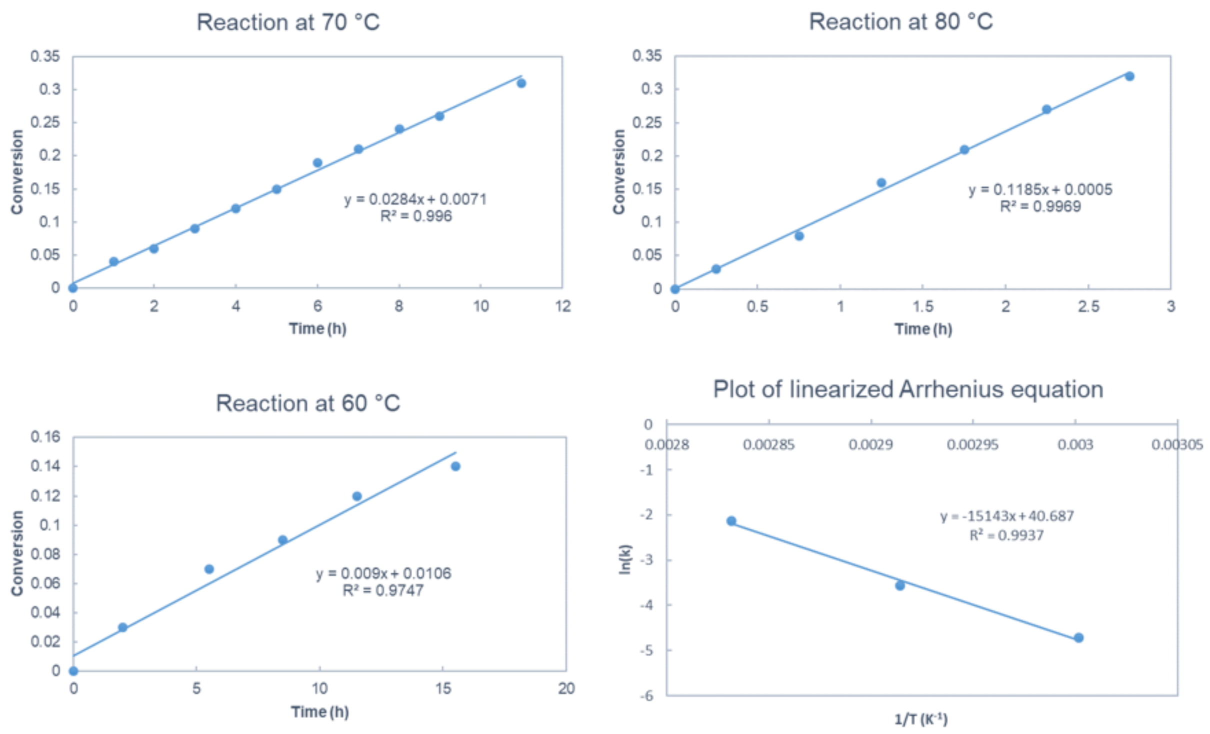

Figure S19. Kinetic experiments of the reaction from **2** to **3**.

2. Single Crystal X-Ray Structure Determination

Single crystal diffraction data were recorded on a Bruker Photon D8 Venture DUO IMS system equipped with a Helios optic monochromator and a Mo IMS microsource (λ = 0.71073 Å) The data collection was performed, using the APEX III & IV software package[S3] on single crystals coated with Fomblin®Y as perfluorinated ether. The single crystals were picked on a micro sampler, transferred to the diffractometer, and measured frozen under a stream of cold nitrogen (100 K). A matrix scan was used to determine the initial lattice parameters. Reflections were merged and corrected for Lorenz and polarization effects, scan speed, and background using SAINT.[S4] Absorption corrections, including odd and even ordered spherical harmonics were performed using SADABS.[S4] Space group assignments were based upon systematic absences, E statistics, and successful refinement of the structures. Structures were solved by direct methods with the aid of successive difference Fourier maps and were refined against all data using the APEX IV software in conjunction with SHELXL-2014[S5] and SHELXLE.[S6] H atoms were placed in calculated positions and refined using a riding model, with methylene and aromatic C–H distances of 0.99 and 0.95 Å, respectively, and Uiso(H) = 1.2·Ueq(C). Non-hydrogen atoms were refined with anisotropic displacement parameters. Full-matrix least-squares refinements were carried out by minimizing Σw(Fo2-Fc2)2 with the SHELXL weighting scheme.[S7] Neutral atom scattering factors for all atoms and anomalous dispersion corrections for the non-hydrogen atoms were taken from International Tables for Crystallography.[S8] The images of the crystal structures were generated by Mercury.[S9] The CCDC numbers 2351352 to 2351356 contain the supplementary crystallographic data for the structures **2** to **9**. These data can be obtained free of charge from the Cambridge Crystallographic Data Centre via https://www.ccdc.cam.ac.uk/structures/.

Table S1 Crystallographic details

	**Compound_2**	**Compound_3**	**Compound_5**
**CCDC-Number**	2351352	2351353	2351354
Chemical formula	$C_{46}H_{87}Al_2FN_4Si_2$	$C_{26}H_{46}AlFN_2Si$	$C_{26}H_{47}AlN_2Si$
M_r	825.33	460.72	442.72
Crystal system, space group	Triclinic, $P\bar{1}$	Monoclinic, $P\,2_1/c$	Orthorhombic, *Pnma*
Temperature (K)	100	100	100
a (Å), α(°)<br>*b (Å)*, β(°)<br>*c (Å)*, γ(°)	11.8137(16), 95.813(4)<br>12.1754(16), 97.979(4)<br>20.635(3), 111.729(4)	14.5764(4), 90<br>9.3652(3), 102.873(1)<br>21.2605(7), 90	17.1935(14), 90<br>15.3076(13), 90<br>10.5747(9), 90
V (Å^3)	2692.6(6)	2829.35(15)	2783.2(4)
Z	2	4	4
F(000)	908	1008	976
D_x (g/cm^3)	1.018	1.082	1.057
Radiation type	Mo $K\alpha$	Mo $K\alpha$	Mo $K\alpha$
μ (mm^{-1})	0.133	0.135	0.130
θ range (°) for cell meas.	2.23–25.52	2.39–25.58	2.34–25.66
Crystal size (mm)	0.215 × 0.186 × 0.128	0.198 × 0.123 × 0.107	0.266 × 0.261 × 0.126
Diffractometer	Bruker Photon CMOS	Bruker Photon CMOS	Bruker Photon CMOS
Radiation source	TXS rotating anode	TXS rotating anode	TXS rotating anode
Monochromator	Helios optic	Helios optic	Helios optic
Absorption correction	Multi-scan	Multi-scan	Multi-scan
T_{min}, T_{max}	0.972, 0.983	0.980, 0.986	0.966, 0.984
θ_{max} (°)	25.802	25.639	25.705
Range of *h*, *k*, *l*	h = -14→14, k = -14→14, l = -25→25	h = -17→17, k = -11→11, l = -25→25	h = -20→20, k = -18→18, l = -12→12
Refinement method	Full-matrix least-squares on F^2	Full-matrix least-squares on F^2	Full-matrix least-squares on F^2
Data/restraints/parameters	10172/296/639	5324/341/391	2746/0/149
Goodness-of-fit on F^2	1.089	1.031	1.041
Final R indices (I>2σ(I))	R_1 = 0.0945, wR_2 = 0.2579	R_1 = 0.0705, wR_2 = 0.2081	R_1 = 0.0498, wR_2 = 0.1495
$\Delta\rho_{max}$, $\Delta\rho_{min}$ (e Å^{-3})	0.632, -0.579	0.672, -0.456	0.428, -0.755

	Compound_6	Compound_9
**CCDC-Number**	2351355	2351356
Chemical formula	$C_{26}H_{45}AlF_2N_2Si$	$C_{46}H_{86}Al_2F_2N_4Si_2$
M_r	478.71	843.32
Crystal system, space group	Monoclinic, $P\ 2_1/c$	Triclinic, $P\bar{1}$
Temperature (K)	100	100
a (Å), α(°)<br>*b (Å)*, β(°)<br>*c (Å)*, γ(°)	14.4396(10), 90<br>9.5167(6), 102.938(2)<br>21.2259(15), 90	10.2063(12), 100.645(5)<br>13.861(2), 98.830(5)<br>20.555(3), 92.867(5)
V (Å^3)	2842.8(3)	2814.8(7)
Z	4	2
F(000)	1040	924
D_x (g/cm^3)	1.119	0.995
Radiation type	Mo $K\alpha$	Mo $K\alpha$
μ (mm^{-1})	0.142	0.131
θ range (°) for cell meas.	3.04–25.66	2.29–25.69
Crystal size (mm)	0.218 × 0.175 × 0.127	0.218 × 0.146 × 0.065
Diffractometer	Bruker Photon CMOS	Bruker Photon CMOS
Radiation source	IMS microsource	IMS microsource
Monochromator	Helios optic	Helios optic
Absorption correction	Multi-scan	Multi-scan
T_{min}, T_{max}	0.971, 0.982	0.972, 0.992
θ_{max} (°)	25.697	25.801
Range of *h*, *k*, *l*	h = -17→17, k = -11→11, l = -25→25	h = -12→12, k = -16→16, l = -25→25
Refinement method	Full-matrix least-squares on F^2	Full-matrix least-squares on F^2
Data/restraints/parameters	5369/174/400	10777/0/531
Goodness-of-fit on F^2	1.059	1.044
Final *R* indices (I>2σ(I))	R_1 = 0.0477, wR_2 = 0.1392	R_1 = 0.0520, wR_2 = 0.1464
$\Delta\rho_{max}$, $\Delta\rho_{min}$ (e Å^{-3})	0.531, -0.518	0.432, -0.276

3. Computational Details

3.1 Computational Methods

All the calculations were performed using the Gaussian 16 software package (version A.03).[S10] We compared the performance of various density functionals to identify the most suitable method for this system. As depicted in Table **S2**, the geometry of crystal structure **2** was optimized by using different density functionals. It was found that the B3LYP(D3) [S11]/def2-SVP [S12] method exhibited the most accurate reproduction of the experimental data, with a minimal relative deviation (RD) value of 1.08%. Therefore, B3LYP(D3) /def2-SVP method proves to be the most suitable for this system, and all calculations in this project were consequently conducted using this method. Hessian calculations for obtaining the vibrational frequencies were performed at the same level of theory as that for the geometry optimization to check whether the optimized geometrical structure is an energy minimum (with no imaginary frequency) or transition state (with one and only one imaginary frequency). The stability of the wavefunction was checked for all optimized structures at the B3LYP(D3)/def2-SVP level. The single-point energy calculations were performed at the B3LYP(D3)/def2-TZVP[S13] level of theory. The relative Gibbs free energy (ΔG) was the outcome of the formula $\Delta G = \Delta E + \Delta G_{ZPE}$, where ΔG_{ZPE} is the Gibbs free energy correction acquired from the B3LYP(D3)/def2-SVP method and the electronic energy ΔE was obtained from single-point energy calculations at the B3LYP(D3)/def2-TZVP level. IRC calculations were used to check further whether the TSs connect the corresponding reactants and products.[S14]

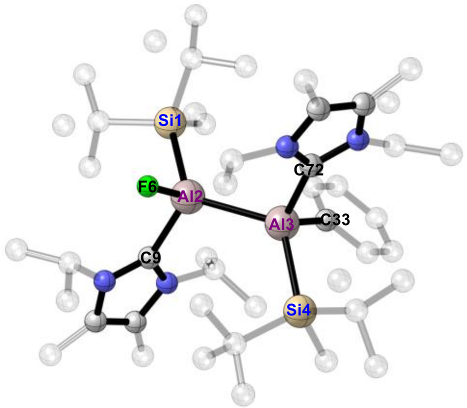

Table S2. Key distances (Å) of experimental and DFT-optimized structures of compound **2**.

Functional	Exp.	B3LYP	M06-2X	PBE0	TPSS	wB97XD	BP86
Si1-Al2	2.566	2.557	2.552	2.561	2.568	2.551	2.554
Al2-C9	2.110	2.117	2.109	2.105	2.107	2.110	2.100
Al2-F6	1.713	1.756	1.743	1.754	1.764	1.754	1.771
Al2-Al3	2.694	2.665	2.659	2.688	2.690	2.665	2.668
Al3-C33	2.057	2.041	2.033	2.037	2.044	2.033	2.043
Al3-Si4	2.564	2.562	2.550	2.566	2.571	2.553	2.551

Al3-C72	2.126	2.151	2.139	2.138	2.139	2.145	2.126
∠Si1-Al2-Al3	121.9	123.4	122.2	123.1	123.1	122.6	122.8
∠Si1-Al2-C9	104.6	103.2	101.0	103.3	103.4	102.3	103.0
∠Al2-Al3-C72	108.0	108.7	109.2	108.7	109.0	109.2	109.0
∠C9-Al2-Al3-C33	169.0	169.1	168.0	167.7	167.5	168.7	168.2
∠F6-Al2-Al3-C33	78.4	80.2	79.8	81.1	81.2	79.8	80.2
∠C9-Al2-Al3-C72	168.1	164.4	163.3	163.8	163.8	164.3	164.5
RD(%)[a]	0.00	1.08	1.23	1.10	1.15	1.13	1.12

[a] RD = $\frac{\sum_{i=1}^{n}\frac{|BL(DFT) - BL(Exp)|}{BL(Exp)}*100\%}{n}$; basis set: def2-SVP.

3.2 Dissociation energies of complexes AlF and AlH

The BDE represents the electronic energy change in a homolytic cleavage process of a specific bond. For example, BDE(A-B) = E(A•) + E(B•) - E(A-B), where the structures of radicals A• and B• are directly taken from A-B without an optimization.

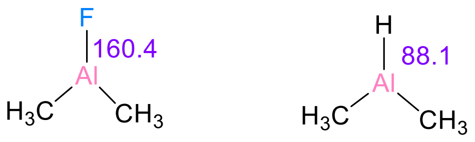

AlF **AlH**

Figure S20. Dissociation energies of complexes AlF and AlH (kcal/mol).

3.3 Gibbs energy profiles for the overall process

As shown in Figure **S21**, the intermediate **INT1** is formed by the 1,2-addition of the C-F bond in fluorobenzene via the transition state **TS1** with a reaction barrier of 29.1 kcal/mol (black line), which does match well with the experimental results. Subsequently, intermediate **INT1** is converted into a more thermodynamically stable product **2** through fast isomerization (purple line). In addition, the possible pathway (red line) for the 1,4-addition of Al=Al unit to the six-membered ring in fluorobenzene has also been examined. In contrast, the thermodynamically unfavorable (ΔG = 9.0 kcal/mol) intermediate **INT1'** was generated in this case through the transition state **TS1'** with a reaction barrier of 33.2 kcal/mol, indicating that the 1, 4-addition of **1** to the benzene ring in fluorobenzene is unfavorable both kinetically and thermodynamically.

According to the experimental results, the final product **3** was exclusively generated after heating the reaction mixture at 65 °C for 4 days (or 100 °C, 5 h). Herein, three possible reaction mechanisms are proposed for the generation of compound **3**: the first path is the direct oxidation addition of **2** with excess fluorobenzene in the reaction system (Figure **S23**); The second path is to firstly undergo migration reaction followed by oxidation addition with fluorobenzene (Figure **S24**); The third path is to firstly dissociate the NHC ligand followed by a migration reaction to ultimately achieve the generation of **3** (Figure **S21**). Compared to the third path, the first two mechanisms require higher reaction barriers (Figures **S23**-**S24**).

Specifically, intermediate **INT1** firstly undergoes NHC dissociation at different positions, resulting in the formation of the corresponding intermediates **INT-deN-a** and **INT-deN-b**, with the endergonicity of 17.9 kcal/mol and 19.7 kcal/mol, respectively. Subsequently, **INT-deN-a** undergoes a phenyl migration to form intermediate **P-deN-a** through transition state **TS-deN-a** with a reaction barrier of 34.0 kcal/mol, whereas **INT-deN-b** undergoes a fluoride migration to form intermediate **P-deN-b** through transition state **TS-deN-b** with a reaction barrier of 28.2 kcal/mol. This indicates that the F-migration is kinetically more favorable. After that, the dissociated NHC will rapidly coordinate to generate a thermodynamically more stable intermediate **INT4** with an exergonicity of 63.2 kcal/mol. Then, self-dimerization of alumylene complex is assumed to achieve thermodynamically more stable product, and the generation of final product **3** is also a thermodynamically favorable process, with an exergonicity of 154.4 kcal/mol.

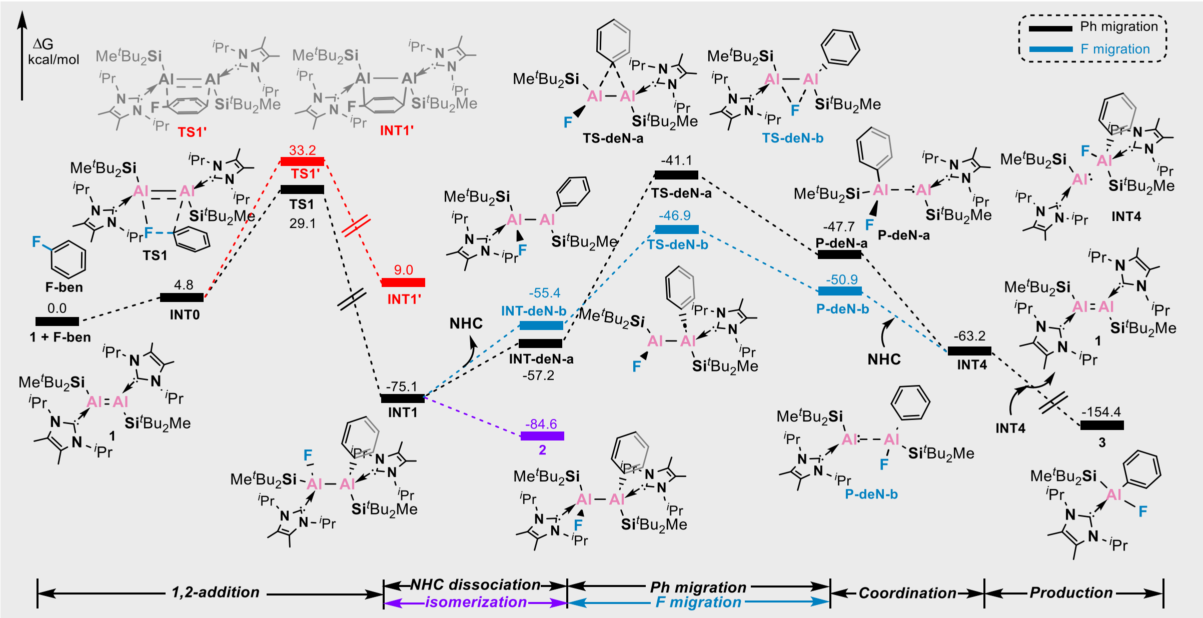

Figure S21. Gibbs energy profiles for the dialumene-mediated C-F bond activation at the B3LYP(D3)/def2-TZVP // B3LYP(D3)/def2-SVP level.

Also, the effect of the dissociated NHC was considered here (Figure **S22**). **INT1** firstly undergoes NHC dissociation reactions at different positions, resulting in the formation of **INT-deN-a$_0$** and **INT-deN-b$_0$**, with the endergonicity of 16.6 kcal/mol and 22.1 kcal/mol, respectively. Subsequently, **INT-deN-a$_0$** undergoes a phenyl migration to form intermediate **P-deN-a$_0$** through transition state **TS-deN-a$_0$** with a reaction barrier of 35.8 kcal/mol, whereas **INT-deN-b$_0$** undergoes an F-migration to form intermediate **P-deN-b$_0$** through transition state **TS-deN-b$_0$** with a reaction barrier of 30.8 kcal/mol. This also indicates that F-migration is kinetically more favorable. After that, the dissociated NHC will rapidly coordinate to generate a thermodynamically more stable intermediate **INT4** with an exergonicity of 63.2 kcal/mol. Then, self-dimerization of alumylene complex is assumed to achieve thermodynamically more stable product, and the generation of final product **3** is also a thermodynamically favorable process, with an exergonicity of 154.4 kcal/mol.

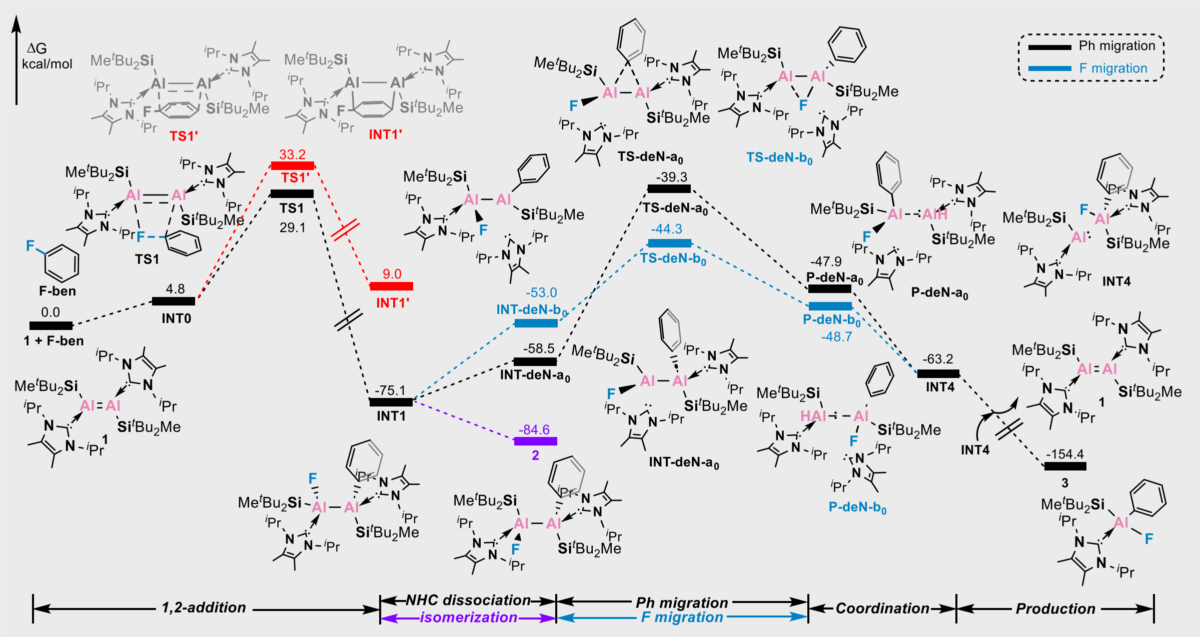

Figure S22. Gibbs energy profiles for the dialumene-mediated C-F bond activation at the B3LYP(D3)/def2-TZVP // B3LYP(D3)/def2-SVP level.

3.4 Oxidation addition of 2 with fluorobenzene to form 3

The reaction of **2** with fluorobenzene to generate product **3** via the transition state **TS2a** was calculated (Figure **S23**). During this process, both the C-F bond and the Al-Al bond undergo simultaneous cleavage with a very high reaction barrier of 75.6 kcal/mol, indicating that thermodynamically stable product **2** is difficult to form product **3** through directly oxidative addition with the C-F bond in another fluorobenzene.

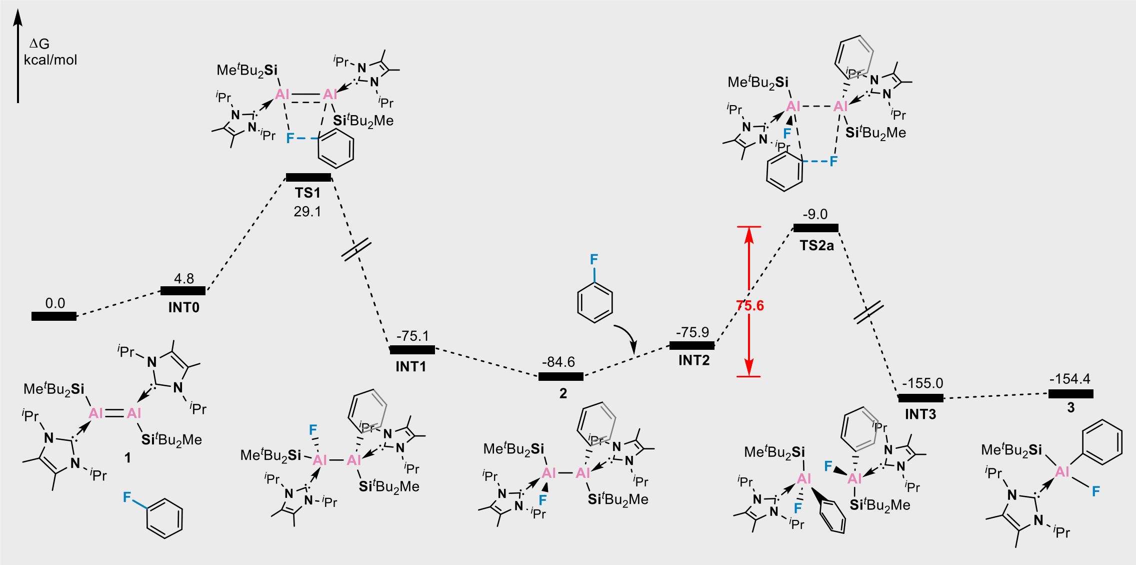

Figure S23. Gibbs energy profiles for the reaction from **2** to **3** via oxidative addition pathway at the B3LYP(D3)/def2-TZVP // B3LYP(D3)/def2-SVP level.

3.5 Formation of 3 via sequential migration reaction and oxidation addition

As shown in Figure **S24**, another possible path from **2** to **3** has been proposed, unfortunately, the calculation results indicate that it is not kinetically favorable. Specifically, intermediate **INT4** is firstly formed through the migration of phenyl group with reaction barrier of 49.8 kcal/mol (transition state **TS2b1**), followed by the separation of intermediate **INT4** to obtain product **3**. Thereafter, intermediate **4** undergoes an oxidative addition reaction with the C-F bond in fluorobenzene to obtain the thermodynamically more stable product **3**, through the transition state **TS2b2** with a reaction barrier of 49.9 kcal/mol. Also, direct oxidative addition of intermediate **INT4** to the C-F bond in fluorobenzene via the transition state **TS2b2'** is also kinetically unfavorable ($\Delta G^{\neq}$ = 50.8 kcal/mol, blue line).

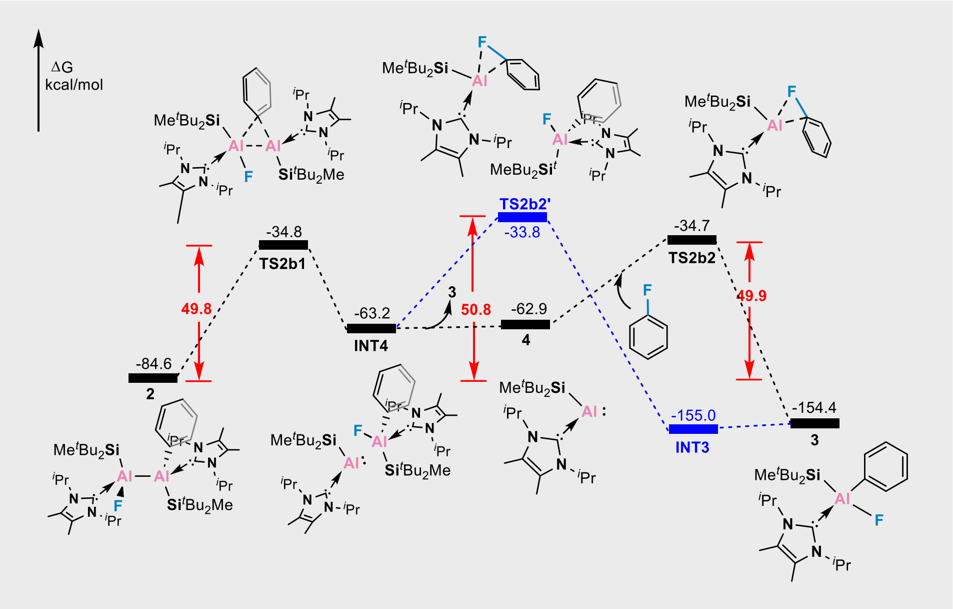

Figure S24. Gibbs energy profiles for the reaction from **2** to **3** via sequential migration reaction and oxidation addition at the B3LYP(D3)/def2-TZVP // B3LYP(D3)/def2-SVP level.

3.6 Gibbs energy profiles for C-F vs. C-H bond activation

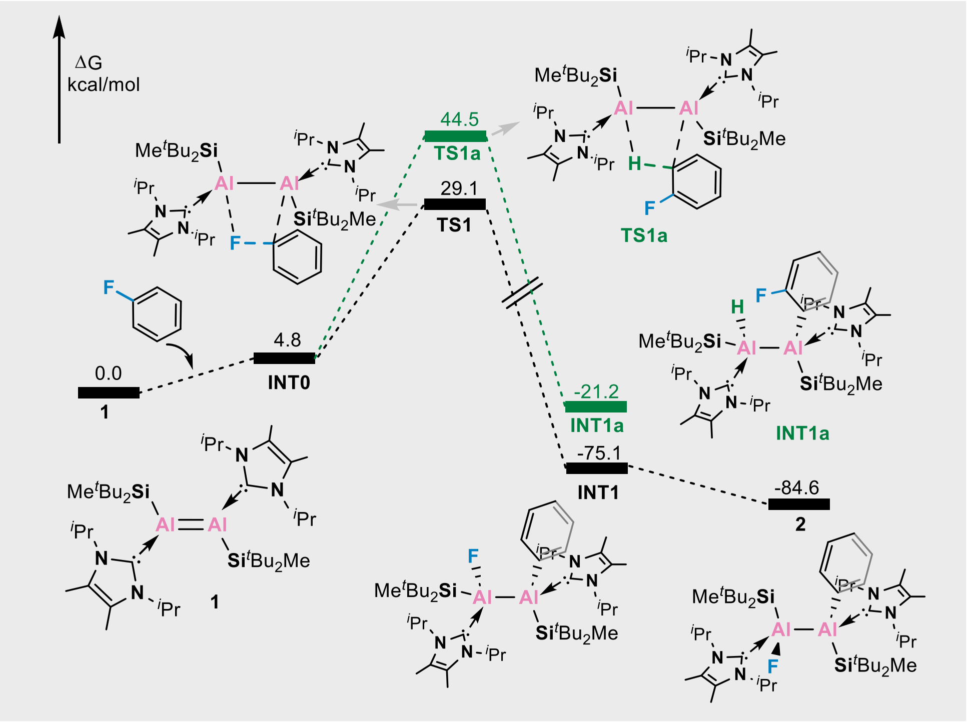

Figure S25. Gibbs energy profiles for the dialumene-mediated C-F *vs.* C-H bond activation at the B3LYP(D3)/def2-TZVP // B3LYP(D3)/def2-SVP level.

3.7 Gibbs energy profiles for the reaction from 5 to 3

When using **5** as the reference point and directly converting **5** to **3**, the specific calculation is as follows. As shown in the Figure **S26**, the calculation results indicate that the intermediate **4** is formed by the dissociation of benzene in compound **5**. This intermediate then undergoes an oxidative addition reaction with the C-F bond in fluorobenzene to obtain product **3**, which is thermodynamically and dynamically feasible. At the same time, such a dissociation process is also beneficial for the dimerization of **4** to form **1**. This also provides a certain degree of support for the mechanism in the manuscript (Figure **2**).

Unfortunately, **5** is prone to necrosis and inactivation in experiments. This is an important reason for the lack of the conversion from **5** to **3**.

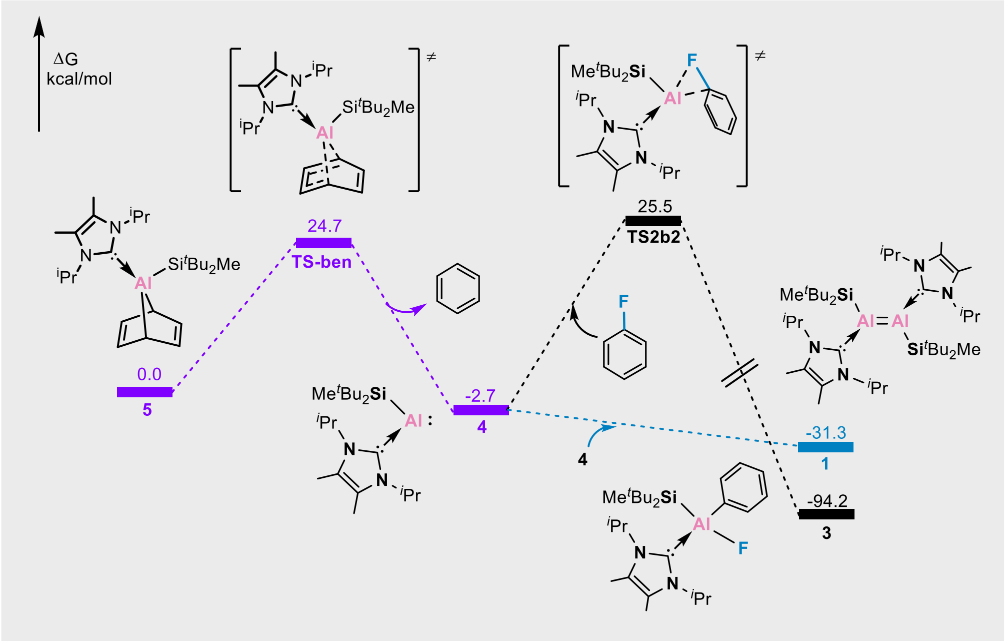

Figure S26. Gibbs energy profiles for the reaction from **5** to **3** via sequential dissociation reaction and oxidation addition at the B3LYP(D3)/def2-TZVP // B3LYP(D3)/def2-SVP level.

4. Appendix

Cartesian Coordinates

Fluorobenzene

B3LYP(D3)/def2-SVP

E = -331.24989 a.u.

C	0.93874300	-0.00000400	-0.00000100
C	0.26143000	1.21844600	0.00000200
C	-1.13665300	1.21056200	-0.00000200
C	-1.83866000	0.00000100	0.00000000
C	-1.13665900	-1.21055700	0.00000200
C	0.26143000	-1.21844800	-0.00000200
H	0.83554200	2.14705600	0.00000200
H	-1.67930300	2.15924200	-0.00000100
H	-2.93105700	0.00000600	0.00000000
H	-1.67930400	-2.15924000	0.00000200
H	0.83552600	-2.14706700	-0.00000200
F	2.28009000	0.00000100	0.00000000

1

B3LYP(D3)/def2-SVP

E = -2855.177003 a.u.

Si	-2.25942800	-2.63820300	0.27945400
Al	-1.09148000	-0.47942900	-0.11430200
Al	1.10595700	0.47940400	-0.27132400
Si	2.24077700	2.62142500	0.28841000
N	-2.91607100	1.63921500	1.13096100
N	-2.95163500	1.83128900	-1.02471800
N	2.83930300	-1.62881900	1.16981200
C	-2.47790300	1.05089500	-0.01575000
C	-2.51761500	-2.92754700	2.16375100
H	-1.55131700	-3.11534000	2.65870600
H	-3.17127600	-3.79328200	2.36647200
H	-2.96813800	-2.04881800	2.65104300
C	-4.08627800	-2.65110200	-0.46842400

C	-4.92805000	-1.63343600	0.33061500
H	-5.02970500	-1.91890800	1.38987800
H	-5.95061800	-1.56757600	-0.08784900
H	-4.49402900	-0.62174900	0.29275700
C	1.86205000	-2.46490100	-3.09419900
H	2.14338000	-3.52602500	-3.02339200
H	1.78457200	-2.20243300	-4.16095300
H	0.86998800	-2.33188200	-2.63933400
C	-2.58372700	1.07576600	2.45740000
H	-2.11944600	0.10669300	2.20605700
C	4.11779500	2.26414600	-1.91528200
H	3.66311900	1.27646900	-2.08404000
H	5.16212100	2.21811400	-2.28056100
H	3.57984400	2.98127100	-2.55243100
C	2.86667900	-1.53864000	-2.41355400
H	2.40491700	-0.53484300	-2.40287800
C	-4.09447300	-2.21595900	-1.94508300
H	-3.63242200	-1.22633200	-2.07429300
H	-5.13195000	-2.15296600	-2.32674500
H	-3.54950100	-2.91676000	-2.59421600
C	0.27253500	-3.92158900	0.07784700
H	0.66457000	-3.00534100	-0.38475500
H	0.91827300	-4.75967300	-0.24824600
H	0.39924400	-3.82094300	1.16693400
C	-1.27360600	-4.35255100	-1.84104700
H	-2.26900300	-4.69285300	-2.16629700
H	-0.54336800	-5.10856000	-2.18846300
H	-1.05465100	-3.41080000	-2.36956200
C	-4.76269000	-4.02973000	-0.35348400
H	-4.27275700	-4.78422900	-0.98757000
H	-5.81864500	-3.97014300	-0.68074500
H	-4.76463500	-4.41005000	0.68093900
C	-3.62757500	2.80358900	0.85080400
C	-2.72470100	1.49044800	-2.44668100
H	-2.30229600	0.47157700	-2.39279600
C	-3.64818900	2.92835200	-0.51639900
C	-3.82333200	0.79051900	3.30467500
H	-4.27893200	1.70179500	3.71845400
H	-3.53545100	0.15203100	4.15411400
H	-4.58131100	0.25062200	2.71797800
C	-1.63125400	-5.50028500	0.36154200
H	-1.53298400	-5.45230100	1.45771400
H	-0.99268700	-6.33570300	0.01484300
H	-2.67205800	-5.77119100	0.13279300
N	3.02037600	-1.84784300	-0.97526500
C	4.08217000	2.65583700	-0.42659800
C	4.75597200	4.03059700	-0.26016500
H	4.27745300	4.80316100	-0.88106400
H	5.81784600	3.98041800	-0.56952100
H	4.73881200	4.38083400	0.78470700
C	2.47179600	-1.05969700	-0.01085000
C	-1.19520400	-4.18445400	-0.31301200
C	4.21118500	-1.42995300	-3.13470200
H	4.66675900	-2.40915100	-3.34097900
H	4.92195500	-0.82445200	-2.55246800
H	4.05677900	-0.92934000	-4.10275500
C	1.19270700	4.19201400	-0.27123100
C	4.14959100	-3.66125800	2.02316200
H	5.04356500	-3.21803000	2.49166100
H	4.44715500	-4.62982200	1.59889000
H	3.42090300	-3.85938700	2.82115500
C	2.46429200	2.85957500	2.18379500
H	1.48533500	3.00674000	2.66755000
H	3.09152600	3.73594700	2.42150500
H	2.93117200	1.98046700	2.65233200
C	3.69703700	-2.92757300	-0.40864800
C	4.40349200	-3.99962500	-1.17257100
H	3.80433900	-4.36199400	-2.01954800
H	4.60203400	-4.85854700	-0.51718200
H	5.37267800	-3.65877300	-1.57205700
C	3.58370400	-2.78630400	0.95234700
C	1.61872000	5.48321200	0.45555200
H	1.49496500	5.40066600	1.54696600
H	0.99111400	6.33205400	0.12158100
H	2.66532400	5.75707600	0.25940000
C	1.34167800	-1.86242000	3.14878000
H	0.45152500	-1.88388400	2.50173400
H	1.05927600	-1.37704800	4.09622700
H	1.65186400	-2.89333500	3.37510900
C	-1.65102300	2.36666100	-3.08697600
H	-1.87403200	3.44090600	-3.00966700
H	-1.55739300	2.11154200	-4.15433400
H	-0.68086100	2.16926600	-2.60646000
C	-1.50513800	1.89253700	3.16544700
H	-0.59434000	1.90344400	2.54728800
H	-1.25765000	1.41806100	4.12795800
H	-1.81830900	2.92765400	3.36769000
C	2.43764100	-1.04584500	2.46888600
H	1.96972900	-0.09056800	2.17749100
C	-0.28206700	3.92083800	0.08547600

H	-0.66872100	3.02312200	-0.41485100
H	-0.92021100	4.77202600	-0.22154200
H	-0.42716700	3.78227900	1.16812500
C	-4.02922400	1.41556700	-3.24322000
H	-4.79992400	0.86021300	-2.68782400
H	-3.84005900	0.87799800	-4.18482600
H	-4.42738500	2.40596000	-3.50619800
C	3.63694000	-0.72951100	3.36261700
H	4.08321000	-1.62656000	3.81577000
H	3.30680900	-0.07597400	4.18458400
H	4.41464600	-0.19479100	2.79737600
C	-4.27754700	4.00488000	-1.33888800
H	-3.61279400	4.34138200	-2.14682700
H	-4.49950000	4.87702400	-0.70890200
H	-5.22481800	3.68093700	-1.80005900
C	4.91179800	1.61699500	0.35802300
H	4.99978700	1.87686400	1.42507800
H	5.93995500	1.55985200	-0.04800700
H	4.47728200	0.60704200	0.29129700
C	-4.24789600	3.69886200	1.87332000
H	-5.16849700	3.26687200	2.29877500
H	-4.51691300	4.66104000	1.41684600
H	-3.56344100	3.90797600	2.70727100
C	1.29929800	4.41426100	-1.79059100
H	2.30207700	4.76100900	-2.08492200
H	0.57935200	5.18686700	-2.12313500
H	1.08551500	3.49439300	-2.35736200

INT0

B3LYP(D3)/def2-SVP

E = -3186.448511 a.u.

Si	-2.28297600	2.56775300	-0.91421100
Al	-1.15925400	0.42249500	-0.34312200
Al	1.02886900	-0.54165300	-0.52910800
Si	2.16976500	-2.74042700	-0.66181000
N	-2.89406000	-1.80728400	-1.56539700
N	-3.07740300	-1.85227600	0.58975200
N	2.83104100	1.41550100	-2.02698200
C	-2.54424700	-1.13245300	-0.43574700
C	-2.69330000	2.65267500	-2.79003000
H	-1.76196100	2.71421900	-3.37624200
H	-3.31117800	3.53041500	-3.04601700
H	-3.23143400	1.75540700	-3.12780600
C	-4.02605300	2.71338100	-0.00593700
C	-4.93054300	1.59275700	-0.56240500
H	-5.14207500	1.72794000	-1.63560700
H	-5.90513900	1.58629100	-0.03761000
H	-4.48166400	0.59538700	-0.43491400
C	1.79192900	2.58668500	2.16245800
H	2.02641600	3.63949800	1.94787600
H	1.76522800	2.45165400	3.25359500
H	0.78963800	2.36303700	1.77068200
C	-2.55890200	-1.29215400	-2.91025300
H	-2.01075100	-0.35950600	-2.68795600
C	4.04014900	-2.09840800	1.48125300
H	3.56276200	-1.10910500	1.51569700
H	5.08379000	-1.97874900	1.83074400
H	3.52386100	-2.73777700	2.21257300
C	2.80523300	1.62036900	1.55552800
H	2.35596100	0.61619000	1.62547700
C	-3.87932200	2.51445700	1.51468900
H	-3.31173900	1.60610400	1.76421900
H	-4.87344300	2.43430400	1.99522900
H	-3.35514400	3.35272600	1.99476100
C	0.27847300	3.80123600	-1.05408800
H	0.68915500	2.94739200	-0.50019700
H	0.95878700	4.65986500	-0.89291500
H	0.32274400	3.55014500	-2.12528600
C	-1.12040700	4.52560000	0.89276100
H	-2.07707000	4.95741600	1.22478000
H	-0.33905400	5.28605200	1.08484600
H	-0.90683800	3.65963500	1.53795200
C	-4.72469500	4.06127900	-0.26432100
H	-4.17861400	4.90317700	0.18784900
H	-5.73980300	4.06125300	0.17770900
H	-4.83899700	4.27361600	-1.33984200
C	-3.60981500	-2.96119200	-1.25375800
C	-2.96726700	-1.40554900	1.99541300
H	-2.31761400	-0.51893900	1.92591200
C	-3.73127500	-2.98659000	0.11330000
C	-3.80999400	-0.92317800	-3.70884300
H	-4.36100700	-1.80157200	-4.07708500
H	-3.51585700	-0.32744400	-4.58636600
H	-4.49344900	-0.31486000	-3.09802600
C	-1.61625400	5.37436600	-1.41184700
H	-1.57621800	5.18875800	-2.49675900
H	-0.95512900	6.23842700	-1.20625500
H	-2.64229900	5.68440600	-1.16544200
N	2.96850600	1.81286600	0.09659300

C	4.00655900	-2.69083300	0.06096400
C	4.67311700	-4.07844900	0.09097900
H	4.18996500	-4.75190200	0.81539400
H	5.73510800	-3.99074100	0.39141200
H	4.65395300	-4.57410700	-0.89298700
C	2.43145500	0.95203400	-0.81016100
C	-1.15215100	4.14843700	-0.59979200
C	4.14608200	1.58777100	2.29176600
H	4.57887700	2.58814900	2.43366000
H	4.87565300	0.95780700	1.76121000
H	3.98353100	1.15640200	3.29018900
C	1.08645900	-4.17166200	0.14451700
C	4.18536900	3.35195200	-3.02031300
H	5.07961600	2.85484000	-3.43073700
H	4.49403400	4.34698200	-2.67217100
H	3.47694900	3.49583900	-3.84776300
C	2.39602700	-3.27855800	-2.49404600
H	1.41848600	-3.51784600	-2.94275200
H	3.03925400	-4.16942900	-2.59780800
H	2.84087100	-2.47160000	-3.09752500
C	3.66676400	2.83566600	-0.54555100
C	4.35811500	3.96949100	0.13880000
H	3.74311600	4.39523700	0.94375300
H	4.56275000	4.77344600	-0.58139200
H	5.32212400	3.66793700	0.58002000
C	3.58268200	2.58015600	-1.89192200
C	1.50547200	-5.57881900	-0.32325900
H	1.42337600	-5.68836200	-1.41638000
H	0.84661500	-6.34441800	0.13042800
H	2.53773800	-5.83077300	-0.04110500
C	1.36861200	1.51380900	-4.04141900
H	0.47560700	1.62624200	-3.40731100
H	1.07721900	0.95034500	-4.94173600
H	1.71231500	2.50874200	-4.36084300
C	-2.25092700	-2.42037800	2.88562500
H	-2.86829100	-3.30353300	3.10328600
H	-2.00614000	-1.94017000	3.84401300
H	-1.31175200	-2.75480600	2.42526000
C	-1.57608600	-2.19425300	-3.64998900
H	-0.65116100	-2.28951200	-3.06039100
H	-1.31395200	-1.72936700	-4.61344900
H	-1.98318300	-3.19417000	-3.85866300
C	2.43639900	0.73316600	-3.27812500
H	1.94130900	-0.18500700	-2.91513000
C	-0.37351700	-3.94470500	-0.29220100
H	-0.74937100	-2.96634500	0.03737600
H	-1.03589400	-4.71901600	0.14128800
H	-0.49239400	-3.99229700	-1.38635800
C	-4.31439800	-0.95407300	2.56231600
H	-4.82366000	-0.27002200	1.87007600
H	-4.13784500	-0.41308700	3.50383000
H	-4.98533800	-1.80013300	2.77728600
C	3.63992800	0.31031000	-4.11991600
H	4.12236000	1.15449500	-4.63396100
H	3.30459300	-0.39791300	-4.89334900
H	4.39014100	-0.19952100	-3.49705000
C	-4.34899100	-4.04661800	0.96539100
H	-3.58304000	-4.69140100	1.42724500
H	-4.99743100	-4.69052000	0.35598900
H	-4.96342900	-3.62760400	1.77375600
C	4.84068800	-1.77278500	-0.85793800
H	4.92110200	-2.17377600	-1.88100100
H	5.87107500	-1.66936300	-0.46712600
H	4.41257900	-0.76010900	-0.92511800
C	-4.04315100	-4.00133100	-2.23482800
H	-4.51334600	-3.57044000	-3.12842800
H	-4.77239800	-4.67847500	-1.76989600
H	-3.19132800	-4.61633100	-2.57004800
C	1.15073400	-4.10816100	1.68125700
H	2.13624400	-4.41416800	2.06541100
H	0.40326500	-4.78797600	2.13329500
H	0.94754900	-3.09178600	2.05779900
C	-0.44653600	0.70854000	4.26575500
C	0.39458700	-0.21391300	3.64838000
C	1.65356300	-0.44364200	4.21088100
C	2.05777300	0.24746600	5.35888700
C	1.19714100	1.18035500	5.94975700
C	-0.07073000	1.41535500	5.40456100
H	0.09025900	-0.68263900	2.71010800
H	2.32485900	-1.15870600	3.73001900
H	3.04403800	0.06228400	5.79126900
H	1.50877500	1.72788500	6.84268800
H	-0.76647000	2.13312000	5.84338400
F	-1.67064900	0.93104700	3.74107900

TS1

B3LYP(D3)/def2-SVP

E = -3186.413951 a.u.

Si	-2.01026900	2.93261200	-0.19292300

Al	-1.12876400	0.58718000	-0.10615800
Al	1.09834400	-0.47751600	0.06497600
Si	1.83206700	-2.76348700	-0.66465300
N	-3.06521700	-1.11795400	-1.66075600
N	-3.43501100	-1.59393000	0.41777600
N	2.71917800	1.24743700	-1.98258100
C	-2.73210900	-0.74751800	-0.38861800
C	-2.38129500	3.45446500	-2.00445400
H	-1.44806700	3.57260300	-2.57750300
H	-2.91547000	4.41873100	-2.04192100
H	-2.99924800	2.71492300	-2.53181400
C	-3.72538900	3.13875700	0.74878700
C	-4.76469700	2.30243800	-0.02850000
H	-4.94963000	2.70861600	-1.03637500
H	-5.73532500	2.29845900	0.50311400
H	-4.44813000	1.25362300	-0.14562600
C	3.39749200	2.75002800	2.24188600
H	4.23107700	3.44810700	2.08227300
H	3.34660100	2.54192300	3.31908200
H	2.46338500	3.24449500	1.94483500
C	-2.55038100	-0.40058300	-2.84470500
H	-1.89926300	0.38087700	-2.40937700
C	3.68625200	-2.76998300	1.56833500
H	3.31629600	-1.75447800	1.78010400
H	4.72511600	-2.83585100	1.94544900
H	3.07664000	-3.45451700	2.17520800
C	3.58644300	1.42679600	1.49841800
H	2.74529300	0.76606100	1.76881200
C	-3.63587000	2.60526700	2.19139000
H	-3.22425300	1.58732200	2.23727800

H	-4.63725600	2.59017300	2.66264700
H	-2.98929400	3.23123200	2.82316200
C	0.68968100	3.80857900	-0.23338100
H	1.00014300	2.80504000	0.08981800
H	1.49115400	4.51480700	0.05659800
H	0.65105500	3.80341100	-1.33438000
C	-0.50649800	4.19897400	1.92969700
H	-1.38012900	4.64257300	2.43188100
H	0.37830200	4.78268500	2.24900200
H	-0.39280400	3.17335500	2.31215800
C	-4.22008700	4.59619600	0.79547500
H	-3.57432100	5.22900000	1.42322200
H	-5.23720700	4.64379700	1.22980900
H	-4.27260300	5.05595600	-0.20487600
C	-3.93580600	-2.20485600	-1.65524000
C	-3.46381000	-1.46733800	1.89266800
H	-2.67093600	-0.74558600	2.11176400
C	-4.17525800	-2.50294400	-0.33917700
C	-3.66388900	0.30394900	-3.62054400
H	-4.28195600	-0.39284600	-4.20579900
H	-3.21804800	1.02092500	-4.32655200
H	-4.32123800	0.86044100	-2.93668500
C	-0.95990400	5.66489900	-0.05861700
H	-1.01417100	5.74659900	-1.15586800
H	-0.15744000	6.34917400	0.27829000
H	-1.90550600	6.04791100	0.35086100
N	3.44340700	1.57535600	0.03000600
C	3.62914600	-3.11270600	0.06667400
C	4.09652300	-4.56684100	-0.12812400
H	3.49462200	-5.27785300	0.45798000

H	5.14611000	-4.68310400	0.20479600	H	1.23707000	2.65494600	-3.86403400
H	4.05499200	-4.88139500	-1.18383700	C	-3.10743100	-2.76846300	2.61996400
C	2.54216900	0.84848800	-0.69041100	H	-3.95407900	-3.46562800	2.68380700
C	-0.65288100	4.22524700	0.39773500	H	-2.80210300	-2.52443900	3.64822000
C	4.88574600	0.71835900	1.88627200	H	-2.26841800	-3.28406200	2.13232500
H	5.76850600	1.36541100	1.76529900	C	-1.64029500	-1.27667700	-3.70145800
H	5.03576400	-0.19161400	1.29251200	H	-0.79977300	-1.64347400	-3.09330400
H	4.82639400	0.42331200	2.94497700	H	-1.22687800	-0.67522900	-4.52551300
C	0.55348000	-4.17755400	-0.15722000	H	-2.16658800	-2.13274900	-4.14597900
C	4.15145900	2.89569900	-3.33298000	C	1.90734400	0.70803500	-3.09340300
H	4.76697100	2.23346500	-3.96278700	H	1.39233100	-0.15192200	-2.63269900
H	4.75366700	3.78245900	-3.09479400	C	-0.86125700	-3.65291300	-0.45693200
H	3.30015200	3.23279600	-3.94119500	H	-1.08681800	-2.75046400	0.12630300
C	2.04071600	-3.01090000	-2.56109400	H	-1.62544700	-4.41501400	-0.20688500
H	1.06399800	-2.97089600	-3.06998500	H	-0.99708000	-3.39455100	-1.51931900
H	2.50148100	-3.98422600	-2.80200200	C	-4.79090400	-0.87112400	2.37111000
H	2.67284000	-2.22864000	-3.00237800	H	-5.01899200	0.06155300	1.83940900
C	4.17335700	2.42664000	-0.79798000	H	-4.72043000	-0.64079900	3.44509900
C	5.23561700	3.37884000	-0.35000400	H	-5.63187600	-1.56746200	2.23123200
H	4.81107600	4.27718600	0.12660900	C	2.75618500	0.18936100	-4.25646000
H	5.83076200	3.70998100	-1.21143400	H	3.10090400	0.99031400	-4.92570000
H	5.92763400	2.91769200	0.36766500	H	2.14905600	-0.50364400	-4.85817800
C	3.70724700	2.22584800	-2.07229500	H	3.63396700	-0.36408300	-3.89109100
C	0.75600400	-5.47738700	-0.96062600	C	-4.99456900	-3.62341700	0.21266200
H	0.60453300	-5.32316100	-2.04064000	H	-4.36002400	-4.43289800	0.60804400
H	0.02365500	-6.24235700	-0.63671600	H	-5.62217700	-4.05359700	-0.57916300
H	1.75828400	-5.90896100	-0.82430800	H	-5.66192800	-3.29695200	1.02223000
C	0.82344800	1.69444700	-3.52187100	C	4.61089600	-2.19324400	-0.69186700
H	0.14120800	1.87952100	-2.67902900	H	4.70699300	-2.47956300	-1.75165400
H	0.23698000	1.26529100	-4.34909300	H	5.62256500	-2.25294800	-0.24652900

H	4.29933200	-1.13738600	-0.66371900
C	-4.41235300	-2.94120700	-2.86518400
H	-4.73135600	-2.26865300	-3.67162200
H	-5.27006800	-3.57630400	-2.60631500
H	-3.62726900	-3.60064400	-3.27135200
C	0.64064500	-4.50170400	1.34448800
H	1.57441500	-5.02516200	1.59984900
H	-0.19322200	-5.16436700	1.64864500
H	0.59203400	-3.59581900	1.96629200
C	0.21129800	-0.04854300	2.90458500
C	0.10393800	-1.37151300	3.39003600
C	0.93310500	-1.80048800	4.41832000
C	1.81418600	-0.90915600	5.05913400
C	1.79202600	0.44085900	4.67823700
C	0.97121900	0.88503800	3.64332900
H	-0.55479600	-2.07327600	2.88508100
H	0.90069300	-2.85115600	4.72150500
H	2.46633600	-1.25214100	5.86480400
H	2.41952900	1.16543200	5.20688300
H	0.95410800	1.93478100	3.35498500
F	-1.00825200	0.51247200	2.31778800

TS1’

B3LYP(D3)/def2-SVP

E = -3186.412476 a.u.

Si	-1.88712900	-2.81568300	0.31857900
Al	-1.12106600	-0.46492300	-0.21089800
Al	1.20260800	0.45481400	-0.38636600
Si	2.01983200	2.79231300	0.13239500
N	-2.74316500	1.09443100	1.94719700
N	-3.51980700	1.60159500	-0.00454200
N	2.94830200	-1.05431500	1.72821200
C	-2.58556200	0.82476800	0.61674800
C	-2.17063000	-3.14228100	2.19421400
H	-1.22509200	-3.18034800	2.75599800
H	-2.68672100	-4.10482300	2.35051500
H	-2.79563500	-2.36229600	2.64794200
C	-3.67049700	-3.14585300	-0.45752400
C	-4.67087300	-2.30813800	0.36916600
H	-4.80163300	-2.70965200	1.38656200
H	-5.66793100	-2.31105300	-0.11092300
H	-4.35382100	-1.25742600	0.46756900
C	3.46750000	-3.10639100	-2.29158400
H	4.30395200	-3.78776100	-2.08474400
H	3.37548200	-3.03293200	-3.38558000
H	2.54155300	-3.55176200	-1.90315000
C	-1.87601200	0.50957100	2.98889300
H	-1.34898600	-0.29770300	2.45714900
C	3.73444000	2.86318100	-2.22587000
H	3.47488200	1.81048000	-2.40436000
H	4.72835200	3.04335100	-2.67852200
H	3.00949600	3.47988800	-2.77781500
C	3.68352700	-1.70427100	-1.72002600
H	2.83890000	-1.08747000	-2.03568000
C	-3.72733600	-2.68270300	-1.92295700
H	-3.43403900	-1.63209800	-2.04442900
H	-4.75146200	-2.79320300	-2.32856000
H	-3.05884800	-3.27372800	-2.56823300
C	0.79086200	-3.80302900	0.43216300
H	1.11814900	-2.80742800	0.10349600

H	1.57266700	-4.52924600	0.13642100
H	0.76329300	-3.79912400	1.53251500
C	-0.40076500	-4.27657200	-1.70620700
H	-1.32755500	-4.59348800	-2.20998700
H	0.38164900	-5.01300200	-1.97486600
H	-0.10345700	-3.30656600	-2.12918000
C	-4.11962200	-4.61667400	-0.39344900
H	-3.53118100	-5.25440800	-1.07062100
H	-5.17743800	-4.70703300	-0.70704300
H	-4.04405200	-5.04025900	0.62120200
C	-3.74367900	2.04294700	2.15398700
C	-3.76514100	1.53155200	-1.46079300
H	-2.95350300	0.88334000	-1.80780700
C	-4.23774300	2.36066400	0.91604500
C	-2.65716800	-0.11508100	4.14798300
H	-2.96895100	0.62514000	4.89793000
H	-2.01407100	-0.84855100	4.65698600
H	-3.54980900	-0.64678700	3.78824800
C	-0.92488200	-5.59477700	0.36198300
H	-1.04577400	-5.59809100	1.45693600
H	-0.11272700	-6.30881000	0.12404600
H	-1.84633900	-5.99896500	-0.07719600
N	3.58036400	-1.65762400	-0.24836900
C	3.75695500	3.18720300	-0.72203300
C	4.20182200	4.65015100	-0.53858600
H	3.54528500	5.35383000	-1.07239900
H	5.22341800	4.79470200	-0.93995900
H	4.22587500	4.95139500	0.52100000
C	2.69936600	-0.83890800	0.40007400
C	-0.56696600	-4.19650800	-0.18022500

C	4.95871100	-1.02941800	-2.23113200
H	5.86797300	-1.59999700	-1.98812300
H	5.06135700	-0.01831400	-1.81456200
H	4.90567500	-0.94097000	-3.32722700
C	0.68239500	4.21025900	-0.21615500
C	4.47602000	-2.48269700	3.22146000
H	5.06206400	-1.70938100	3.74224100
H	5.13137100	-3.34993500	3.06820100
H	3.66992000	-2.79971700	3.89840400
C	2.43700600	3.02772900	1.99508100
H	1.54310700	2.97539900	2.63386100
H	2.91248900	4.00623800	2.17304000
H	3.13825400	2.25780600	2.33886800
C	4.35785900	-2.38175900	0.65252000
C	5.39864900	-3.39132800	0.28744800
H	4.95018200	-4.34405700	-0.03725300
H	6.03599200	-3.60432200	1.15577700
H	6.05455400	-3.04056600	-0.52067700
C	3.95325500	-2.00386700	1.90513200
C	0.64668400	4.64682200	-1.69090000
H	1.59261400	5.10353800	-2.01900700
H	-0.14783700	5.40176900	-1.84712500
H	0.42600000	3.80265200	-2.35756000
C	1.09568800	-1.48427800	3.29753600
H	0.41270500	-1.73771100	2.47629300
H	0.50727400	-1.06141100	4.12438400
H	1.56762000	-2.40883100	3.66118200
C	-3.62466900	2.87958400	-2.16762200
H	-4.45940800	3.56129900	-1.95192700
H	-3.62598000	2.69939300	-3.25250100

H	-2.68324500	3.37840900	-1.90257400
C	-0.82487800	1.51993000	3.44382300
H	-0.18032800	1.78456200	2.59547000
H	-0.19715500	1.08699400	4.23611700
H	-1.27702400	2.44034300	3.84129600
C	2.12462600	-0.46810000	2.80661600
H	1.57484100	0.33872800	2.29927900
C	0.93362400	5.45775000	0.65569400
H	0.85726600	5.23499400	1.73117900
H	0.17575100	6.23295900	0.43171900
H	1.92115600	5.90864300	0.47818000
C	-5.08501400	0.83406700	-1.79561300
H	-5.18330300	-0.11103600	-1.24822500
H	-5.08910800	0.59963500	-2.86954300
H	-5.96345300	1.46090200	-1.57710400
C	2.94728300	0.14777700	3.94234800
H	3.25240700	-0.59313000	4.69408000
H	2.33425900	0.90346000	4.45540300
H	3.84709300	0.65276800	3.56355700
C	-5.30577900	3.34913800	0.57493900
H	-4.88716600	4.26315900	0.12421700
H	-5.84881400	3.64470800	1.48221700
H	-6.04214300	2.93892900	-0.12948800
C	4.80917400	2.27741700	-0.05095500
H	5.00665000	2.57164300	0.99173800
H	5.77317300	2.33462100	-0.59159900
H	4.49613600	1.22070700	-0.04159100
C	-4.17953500	2.57114300	3.48308500
H	-4.74708900	1.82454000	4.06090400
H	-4.82929600	3.44475500	3.34187600
H	-3.33050200	2.89347100	4.10248200
C	-0.70705500	3.65269500	0.14198900
H	-0.97101000	2.79002800	-0.48555900
H	-1.48608300	4.42630200	-0.00275900
H	-0.77175400	3.32432600	1.19029800
C	-1.39800300	-0.04932600	-3.40253900
C	-0.82888400	1.24917900	-3.56027000
C	0.53363300	1.37462200	-3.47239100
C	1.34373300	0.22641100	-3.19869500
C	0.77509000	-1.05079300	-3.54716800
C	-0.58787900	-1.18804600	-3.64048500
H	-1.47923200	2.10696500	-3.72899500
H	1.00835800	2.35213700	-3.55728200
H	2.42326200	0.35224300	-3.30051700
H	1.41488700	-1.92387800	-3.68151200
H	-1.07169400	-2.14030400	-3.86151100
F	-2.73671500	-0.16402000	-3.69696200

INT1

B3LYP(D3)/def2-SVP

E = -3186.586077 a.u.

Si	-3.33999900	1.50450600	-0.50137200
Al	-1.41831100	0.05790800	0.29970800
Al	1.27932200	0.04408100	0.22991800
Si	2.82611200	-1.37154000	-1.29616400
N	-2.18207800	-2.60968800	-1.09015400
N	-1.73757500	-2.96287200	0.99779100
N	1.50507600	2.73972900	-1.36105600
C	-1.90721700	-1.97316400	0.08182300
C	-3.65769300	1.79063000	-2.37846100

H	-2.93299900	2.50854300	-2.79201600
H	-4.66316000	2.20723700	-2.55455800
H	-3.57730500	0.86911200	-2.97710400
C	-5.02632200	0.72439400	0.17639700
C	-5.33289500	-0.52792100	-0.66930600
H	-5.41906800	-0.30477700	-1.74488000
H	-6.29230800	-0.98014900	-0.35325900
H	-4.55761700	-1.29748000	-0.53716200
C	1.15297000	3.40847800	3.11542600
H	1.18166400	4.50248100	3.22466500
H	1.32545500	2.96415300	4.10701400
H	0.14481900	3.11042200	2.79050900
C	-2.41405600	-1.85062400	-2.33796300
H	-2.68193300	-0.84906400	-1.97774400
C	5.24330400	-0.34612800	0.06503700
H	4.59947000	0.36206700	0.61024200
H	6.24430300	0.11783500	-0.02256000
H	5.36275600	-1.24436600	0.68970700
C	2.21111700	2.87453300	2.15006900
H	2.19145600	1.78533200	2.23288000
C	-4.91914200	0.27425600	1.64695200
H	-4.08475600	-0.42284500	1.80527900
H	-5.85465600	-0.23004600	1.95721200
H	-4.75596100	1.11644600	2.33318000
C	-1.58783300	3.71644800	-0.10012900
H	-0.85879800	3.03824400	0.36794900
H	-1.37273600	4.73652200	0.27214000
H	-1.38689500	3.72177000	-1.18223600
C	-3.21168100	3.34509600	1.75186800
H	-4.26323600	3.21042100	2.04823600
H	-2.88724500	4.32388800	2.15561100
H	-2.62157400	2.55594900	2.24228100
C	-6.21245000	1.69809200	0.04530100
H	-6.10448900	2.56883400	0.70923300
H	-7.15619500	1.19299300	0.32777500
H	-6.34169000	2.07274900	-0.98321500
C	-2.15226000	-3.99385100	-0.92207300
C	-1.48124600	-2.67545000	2.43275000
H	-1.32901700	-1.59209100	2.46304300
C	-1.88577400	-4.21653600	0.40471400
C	-3.59688600	-2.36225100	-3.16333900
H	-3.33388200	-3.22880800	-3.78541400
H	-3.92245500	-1.55943400	-3.84208500
H	-4.45096500	-2.62695300	-2.52497500
C	-3.96565200	4.37217200	-0.41585800
H	-3.82905700	4.43874500	-1.50730800
H	-3.74386400	5.37413500	0.00018400
H	-5.02973000	4.17180700	-0.22706300
N	1.86927500	3.11940400	0.73352600
C	4.68593300	-0.67396700	-1.33029400
C	5.66902500	-1.63485700	-2.02381800
H	5.82279200	-2.55491000	-1.43923700
H	6.65993600	-1.15435200	-2.13616900
H	5.33691700	-1.92872000	-3.03206000
C	1.69899100	2.10807300	-0.16438300
C	-3.03970400	3.31726100	0.22138800
C	3.64211100	3.30504700	2.47754700
H	3.77997000	4.39685900	2.47351000
H	4.35050200	2.86128500	1.76104200
H	3.90342200	2.93837900	3.48202500

C	2.73208500	-3.30499200	-0.86152600
C	1.24848500	5.11600200	-2.29285100
H	2.10608600	5.23214800	-2.97399700
H	1.03469500	6.09975800	-1.85491500
H	0.37526200	4.83417400	-2.89838800
C	2.38014400	-1.41720400	-3.17898600
H	1.59742300	-2.16306100	-3.37349400
H	3.26240200	-1.72118100	-3.76524600
H	2.02777100	-0.46629300	-3.60124800
C	1.74362200	4.36544400	0.12135200
C	1.76040200	5.67929900	0.83228000
H	0.83025800	5.83719800	1.40251600
H	1.85045500	6.49954500	0.10800900
H	2.59965800	5.76746100	1.53490700
C	1.50854200	4.12444800	-1.20586200
C	3.33522900	-4.19939800	-1.96538200
H	2.86492300	-4.03882100	-2.94717800
H	3.18249200	-5.26488500	-1.70656500
H	4.41707800	-4.05074200	-2.08574400
C	-0.15369800	1.91160600	-3.03732400
H	-0.76542100	1.50121200	-2.22453400
H	-0.24816200	1.24594800	-3.90846500
H	-0.56169300	2.89308500	-3.32067600
C	-0.20773400	-3.33433100	2.96271300
H	-0.31275900	-4.41787400	3.11385100
H	0.04308200	-2.87895000	3.93124100
H	0.64401900	-3.14349200	2.29622800
C	-1.13212300	-1.71853200	-3.15520500
H	-0.33294200	-1.27836700	-2.54491100
H	-1.30679100	-1.06256200	-4.02154800
H	-0.78606100	-2.69301200	-3.53269800
C	1.31297900	1.99774300	-2.62293600
H	1.64374800	0.98594800	-2.36850200
C	1.23724600	-3.65685900	-0.73802100
H	0.73636700	-3.06384600	0.03968600
H	1.10664600	-4.72423800	-0.47610700
H	0.68840300	-3.48851300	-1.67707100
C	-2.72189400	-2.96723600	3.27970600
H	-3.59847600	-2.43775000	2.87821300
H	-2.55027900	-2.61003200	4.30664200
H	-2.95564100	-4.04190900	3.33349600
C	2.20659100	2.50221700	-3.76153400
H	1.77445700	3.37096600	-4.27674300
H	2.31431900	1.70206800	-4.50914800
H	3.21117700	2.76559300	-3.40461900
C	-1.70065000	-5.52531300	1.10060400
H	-0.63555700	-5.73156900	1.29440200
H	-2.09271200	-6.34194600	0.47997400
H	-2.22690100	-5.56155500	2.06418900
C	4.65121500	0.64742100	-2.12363200
H	4.31237000	0.50893800	-3.16134400
H	5.66216600	1.09548600	-2.16969200
H	3.99171700	1.38627900	-1.64205600
C	-2.32519800	-4.99692500	-2.01537500
H	-3.36668300	-5.06738400	-2.36640100
H	-2.02449300	-5.99121300	-1.66003000
H	-1.69446900	-4.75816200	-2.88498400
C	3.41856300	-3.66134300	0.46802500
H	4.49035200	-3.40524400	0.46765600
H	3.34572800	-4.74975200	0.65622000

H	2.95200600	-3.15321800	1.32221400
C	1.75406800	-0.35285700	2.20130700
C	2.95830500	-0.96557500	2.61145100
C	3.25873400	-1.24751200	3.95059800
C	2.35068000	-0.91375300	4.95775900
C	1.14807500	-0.29632800	4.59877500
C	0.86103700	-0.03016100	3.25489900
H	3.70360500	-1.23447200	1.86860000
H	4.20889700	-1.72811200	4.20353100
H	2.57714900	-1.12867600	6.00611400
H	0.42088400	-0.02206300	5.36973300
H	-0.09111600	0.44745800	3.02748700
F	-1.80922200	0.36294000	1.98549500

INT1’

B3LYP(D3)/def2-SVP

E = -3186.455218 a.u.

Si	2.05245600	2.75070600	0.25921900
Al	1.27819700	0.42405700	-0.49374700
Al	-1.29732100	-0.41375000	-0.53990600
Si	-2.12843600	-2.75268200	0.12770100
N	2.73252500	-1.24279000	1.74025000
N	3.61521500	-1.75126200	-0.16221300
N	-2.93069000	1.19308000	1.62198900
C	2.68015600	-0.93867600	0.40996600
C	2.22552500	2.93112500	2.17093100
H	1.26509100	2.89997600	2.70270800
H	2.69888300	3.89424400	2.41980900
H	2.86417500	2.14240900	2.58771700
C	3.89508700	3.15743700	-0.32444900
C	4.82615400	2.16945200	0.41200500
H	4.87671800	2.37056600	1.49396400
H	5.85828900	2.24568800	0.02021200
H	4.50042600	1.12481100	0.28478900
C	-3.79970400	3.03205300	-2.46419300
H	-4.63773400	3.71796800	-2.27925400
H	-3.74606000	2.87214300	-3.55168300
H	-2.86649200	3.51656400	-2.14499900
C	1.84735800	-0.62061200	2.74251700
H	1.42330000	0.24662900	2.22079700
C	-3.98795200	-2.79072600	-2.11899100
H	-3.76996700	-1.73101000	-2.31257000
H	-4.99845300	-2.99788400	-2.52083100
H	-3.27344400	-3.38496800	-2.70800700
C	-3.97571200	1.67689900	-1.77413200
H	-3.20251100	1.01059200	-2.15319700
C	4.07755300	2.95071200	-1.83842000
H	3.80903000	1.93617400	-2.15861300
H	5.13278200	3.12764700	-2.12409200
H	3.46407900	3.64579500	-2.43085700
C	-0.65052500	3.65690800	-0.17268900
H	-0.80893600	2.85389900	-0.90674100
H	-1.37607200	4.46538200	-0.39060800
H	-0.91209200	3.25315000	0.81755700
C	1.03773400	4.74762000	-1.64384800
H	2.00876500	5.25682400	-1.73209400
H	0.25887300	5.48795700	-1.91047600
H	1.00397600	3.95327400	-2.40171200
C	4.33856400	4.58855900	0.03513300
H	3.77049800	5.35077200	-0.51920400

H	5.40557000	4.73322800	-0.22207100
H	4.23451600	4.80801200	1.10991300
C	3.65837200	-2.25489600	1.99405800
C	3.99011900	-1.67702100	-1.59066400
H	3.27994300	-0.97167400	-2.01486300
C	4.22016200	-2.57258900	0.78704300
C	2.59841200	-0.09983300	3.97257900
H	2.78881900	-0.88714000	4.71429300
H	1.98916400	0.67350800	4.46247200
H	3.55718500	0.35933500	3.69246900
C	0.88388800	5.38438400	0.76472700
H	0.59499400	5.09365400	1.78690700
H	0.19779400	6.19562000	0.45324800
H	1.89529600	5.81503700	0.81483700
N	-3.69585500	1.72680700	-0.32321100
C	-3.92650800	-3.11290200	-0.61473800
C	-4.39381500	-4.56454500	-0.39662700
H	-3.78857500	-5.28830600	-0.96179300
H	-5.44101100	-4.68584800	-0.73504700
H	-4.36261600	-4.85895600	0.66468100
C	-2.78643300	0.91488600	0.29359400
C	0.78975400	4.20431200	-0.22447200
C	-5.32442400	1.01757500	-2.07775300
H	-6.17949700	1.63479800	-1.76393500
H	-5.40374800	0.03865500	-1.58717200
H	-5.41014700	0.85974900	-3.16391800
C	-0.85748500	-4.22107300	-0.29832100
C	-4.28033600	2.75068300	3.16233300
H	-4.88991400	2.04684600	3.75058500
H	-4.87461700	3.66212600	3.01686800
H	-3.40887900	3.02611000	3.77279400
C	-2.46025900	-2.98337800	2.01199700
H	-1.54458700	-2.97916400	2.61928200
H	-2.96476800	-3.94537400	2.19672000
H	-3.11989100	-2.19546400	2.39639700
C	-4.37503700	2.52048500	0.60079800
C	-5.39762500	3.55974400	0.26961600
H	-4.93926600	4.45459900	-0.18075700
H	-5.91864000	3.87713800	1.18228600
H	-6.15999500	3.18993400	-0.42916400
C	-3.88578200	2.18703600	1.83462700
C	-1.00875600	-4.74764000	-1.73774900
H	-1.98737900	-5.21744200	-1.91405000
H	-0.24001700	-5.51713500	-1.94318800
H	-0.87525200	-3.95361300	-2.48353100
C	-0.98379300	1.51378700	3.09529100
H	-0.33612600	1.73260600	2.23961200
H	-0.37420400	1.06128500	3.88937800
H	-1.37854800	2.46491200	3.48128800
C	3.81844200	-3.00923500	-2.32329000
H	4.62661900	-3.72376600	-2.11228600
H	3.82869300	-2.81652200	-3.40672100
H	2.85679000	-3.47726900	-2.06968100
C	0.70177700	-1.56416100	3.09826300
H	0.09478200	-1.75876200	2.20751200
H	0.05976900	-1.11947000	3.87091600
H	1.06573800	-2.52734300	3.48414800
C	-2.09855100	0.56485900	2.66558600
H	-1.63906100	-0.29175600	2.15679300
C	-1.03658200	-5.41314100	0.66465400

H	-0.82370800	-5.13999700	1.70984200
H	-0.33603200	-6.22655200	0.39426400
H	-2.05272800	-5.83497500	0.63075500
C	5.36954300	-1.04615000	-1.79699300
H	5.43655600	-0.07758000	-1.28372900
H	5.51175300	-0.86179300	-2.87238800
H	6.19512300	-1.68683600	-1.45149500
C	-2.91172300	0.02129000	3.84512200
H	-3.14532200	0.79567900	4.58807500
H	-2.32481000	-0.75671200	4.35448800
H	-3.85166400	-0.43836600	3.50783500
C	5.24339100	-3.62329600	0.49731700
H	4.79745600	-4.49844800	-0.00163400
H	5.70106800	-3.97076600	1.43277500
H	6.05271000	-3.25221200	-0.14542900
C	-4.91866600	-2.18244100	0.11647600
H	-5.06572300	-2.47757800	1.16715300
H	-5.91235700	-2.21564900	-0.36992800
H	-4.58137500	-1.13346400	0.11316300
C	3.96317100	-2.84715900	3.33293900
H	4.54728000	-2.16343200	3.96899300
H	4.55168500	-3.76519100	3.20655200
H	3.05323500	-3.11923300	3.88609300
C	0.58274600	-3.69313100	-0.14130000
H	0.79459100	-2.87116600	-0.84025000
H	1.31261500	-4.50268000	-0.33815400
H	0.78672700	-3.32173200	0.87458800
C	1.39074700	0.24400700	-2.60627100
C	0.87443400	-1.08966300	-3.07312500
C	-0.45186700	-1.31460500	-3.08828300
C	-1.39738200	-0.24392700	-2.65111200
C	-0.86157800	1.08922000	-3.08578400
C	0.46543500	1.31018600	-3.09953200
H	1.59427300	-1.84119100	-3.39997600
H	-0.85572500	-2.27496800	-3.41318400
H	-2.40193500	-0.43583500	-3.05512400
H	-1.54569200	1.87511500	-3.41183300
H	0.91340700	2.24658200	-3.43538600
F	2.67581400	0.45516800	-3.22444700

2

B3LYP(D3)/def2-SVP

E = -3186.60069 a.u.

Si	2.33851300	2.61755000	0.22921400
Al	1.21937500	0.42109900	-0.45118300
Al	-1.18422500	-0.33240400	0.42011400
Si	-2.00719800	-2.73873400	0.10600300
N	2.99721800	-1.50593200	1.19791400
F	1.25433800	0.41625900	-2.20691200
N	3.52441600	-1.71519600	-0.88913800
N	-3.69980300	1.59720000	0.07518300
C	2.70915900	-1.02568700	-0.04217100
C	2.54205300	2.93752000	2.11383800
H	1.59015000	3.25282200	2.56859900
H	3.26930500	3.74625300	2.29485700
H	2.88661900	2.05199900	2.66372500
C	4.17947600	2.70354900	-0.47722700
C	5.01745700	1.66869500	0.30378800
H	5.07869500	1.91233200	1.37718300
H	6.05462200	1.64042100	-0.08239100

H	4.60743400	0.65147000	0.21549300
C	-1.00429100	1.89401000	-3.38004000
H	-1.57321600	2.51916700	-4.08455100
H	-0.10747600	1.51229000	-3.88409700
H	-0.66650000	2.52350600	-2.55087200
C	2.32641500	-0.99832900	2.41525700
H	1.74178900	-0.13778700	2.06287800
C	-4.31923800	-2.34359600	-1.62811200
H	-3.99591500	-1.29834100	-1.74402700
H	-5.41598200	-2.36602600	-1.77866800
H	-3.86928100	-2.93326200	-2.44285100
C	-1.93674900	-0.71587200	3.38443500
H	-2.57915700	-1.53511100	3.05484600
C	-1.82159500	0.71223200	-2.85974400
H	-1.13418100	0.06810200	-2.29923200
C	-1.24362500	0.05429000	2.42340000
C	4.21519900	2.33619600	-1.97305500
H	3.68652300	1.39651900	-2.18147600
H	5.25967800	2.22495900	-2.32244700
H	3.74688400	3.10880000	-2.59892500
C	-0.20873900	3.85053100	0.00149200
H	-0.58210200	2.91009100	-0.43199200
H	-0.87754600	4.66409800	-0.33973600
H	-0.33167500	3.76905200	1.09350700
C	1.36703800	4.31835400	-1.90378800
H	2.34107500	4.74343200	-2.19142700
H	0.58942500	5.01030900	-2.28048700
H	1.25584400	3.36383100	-2.43916500
C	4.83935700	4.08288900	-0.29732400
H	4.34891900	4.85694300	-0.90672100
H	5.89959900	4.04700300	-0.61468000
H	4.82695600	4.42271200	0.75094200
C	-0.33053300	1.35757200	4.31289200
H	0.30702200	2.17658700	4.66008900
C	3.96552000	-2.50499300	1.13630300
C	3.59434000	-1.44468800	-2.34641700
H	2.93667000	-0.58123100	-2.48589600
C	4.30034500	-2.63801800	-0.18690400
C	3.31532800	-0.47595300	3.45907700
H	3.80365900	-1.28662000	4.01764400
H	2.76305600	0.13859000	4.18517200
H	4.09067700	0.14962800	2.99373000
C	1.64459900	5.47740100	0.30115000
H	1.52705500	5.43330100	1.39545500
H	0.99273400	6.29565400	-0.06170500
H	2.68265000	5.77218500	0.09110600
C	-1.02314000	0.56371900	5.23294600
H	-0.93374200	0.75175400	6.30665100
N	-2.81376200	1.15153100	-1.84736300
C	-3.94801900	-2.89326000	-0.24190000
C	-4.48755300	-4.33109800	-0.11287800
H	-4.08695300	-5.00390800	-0.88326200
H	-5.58958700	-4.33415000	-0.22105700
H	-4.25910300	-4.77433400	0.86889300
C	-2.67423400	0.90944600	-0.51024100
C	-0.45018000	1.10022100	2.94179800
H	0.10831600	1.73277100	2.24990300
C	1.25666800	4.14844700	-0.37724800
C	-2.42457100	-0.11311700	-3.99828500
H	-2.96486800	0.50782200	-4.72639200

H	-3.10377000	-0.88975100	-3.62709500
H	-1.60403100	-0.60968000	-4.53658800
C	-0.91079200	-3.69204700	-1.22419000
C	-5.65924900	3.11503500	-0.58520400
H	-6.53728600	2.51199200	-0.30488200
H	-5.93008900	3.69948800	-1.47453200
H	-5.47021400	3.82610700	0.23144000
C	-1.80491800	-3.81560200	1.69098800
H	-0.86796300	-3.62517500	2.23151700
H	-1.82682600	-4.88724800	1.43063300
H	-2.62651500	-3.63853600	2.40160000
C	-3.90742600	1.97934800	-2.09632200
C	-4.39242700	2.39429100	-3.44780600
H	-3.58710000	2.79311600	-4.07893000
H	-5.15330500	3.17947800	-3.34668200
H	-4.85622800	1.55459600	-3.98986100
C	-4.46228600	2.26946800	-0.87778800
C	-1.48092900	-5.06679100	-1.61558300
H	-1.68618500	-5.70407400	-0.74057100
H	-0.76122600	-5.61306800	-2.25555400
H	-2.41517900	-4.97500100	-2.19093200
C	-3.32195700	2.97383400	2.10603800
H	-2.27219100	3.09431300	1.81038900
H	-3.35989700	2.94334600	3.20538600
H	-3.88724900	3.85544600	1.76464200
C	-1.83534000	-0.47343300	4.75967800
H	-2.38629200	-1.10140500	5.46668200
C	2.99765300	-2.58061600	-3.17692100
H	3.58185500	-3.51117900	-3.11535200
H	2.96805900	-2.27499900	-4.23424300
H	1.97111900	-2.79169500	-2.85796800
C	1.33802600	-2.01255000	2.98043900
H	0.62995400	-2.32353300	2.20401600
H	0.75952700	-1.55007800	3.79184800
H	1.84036100	-2.90760500	3.37685500
C	-3.88839900	1.67120200	1.54238400
H	-3.26238400	0.86103400	1.92411500
C	0.47888700	-3.90544400	-0.58821400
H	0.93515900	-2.95327400	-0.27545900
H	1.16956900	-4.37868500	-1.31106400
H	0.43548100	-4.56380800	0.29334700
C	5.00686400	-1.05196800	-2.79066800
H	5.46004600	-0.33929600	-2.08757600
H	4.94668100	-0.55864700	-3.77247900
H	5.67525800	-1.91813900	-2.89855300
C	-5.32169300	1.39236300	2.00663900
H	-5.97355500	2.27495500	1.94887100
H	-5.28489700	1.08245500	3.06218800
H	-5.78223200	0.57444600	1.43512900
C	5.32098100	-3.55194000	-0.78489400
H	4.96602500	-4.02045000	-1.71214100
H	5.55987700	-4.35829500	-0.07818800
H	6.26221900	-3.02733300	-1.01562300
C	-4.65791700	-2.02799200	0.81729500
H	-4.48416500	-2.39160900	1.84282200
H	-5.75322900	-2.03220400	0.65526500
H	-4.32074800	-0.98431900	0.77347000
C	4.53534000	-3.23658700	2.30841700
H	5.26048100	-2.62178100	2.86584300
H	5.06337400	-4.13703400	1.96668300

H	3.75839300	-3.55700200	3.01534000
C	-0.74191400	-2.85565500	-2.50418300
H	-1.70800100	-2.62720300	-2.97543600
H	-0.14559300	-3.40748800	-3.25547200
H	-0.22399800	-1.90307600	-2.31151100

INT2

B3LYP(D3)/def2-SVP

E = -3517.863932 a.u.

Si	1.80024300	-0.24709500	-3.01736200
Al	0.82612200	0.12654800	-0.68833500
Al	-1.74982400	0.25850200	0.01342200
Si	-2.76146500	1.20040100	2.20176200
N	1.60202800	3.11320400	-0.45898600
F	1.64252800	-1.18605500	0.15033700
N	2.64571100	1.99009300	1.07014400
N	-3.81273400	-2.02766400	-0.59633800
C	1.81784600	1.84820800	0.00025500
C	1.24723400	0.88207400	-4.46845100
H	0.21884900	0.64161600	-4.77909200
H	1.89554200	0.73286600	-5.34788700
H	1.27189400	1.95149100	-4.21894400
C	3.76730400	-0.02935300	-2.95017700
C	4.06994000	1.47982300	-2.85873200
H	3.72557500	2.02525000	-3.75172100
H	5.16056200	1.64938400	-2.77511900
H	3.60064600	1.93849000	-1.97492600
C	0.29407300	-3.96502300	0.08038300
H	0.11606200	-5.04089300	0.21722000
H	1.30151300	-3.73996400	0.45047800
H	0.27002100	-3.73713900	-0.98931600
C	0.74408300	3.39663800	-1.63517600
H	0.53577700	2.40394600	-2.05840300
C	-1.16023300	-0.48918900	3.89857800
H	-0.67591500	-0.82494900	2.96805100
H	-1.06620500	-1.30978100	4.63506700
H	-0.57057700	0.35745400	4.28510500
C	-3.64636400	2.22430600	-1.45759900
H	-4.16392500	2.35896600	-0.50583400
C	-0.70003000	-3.10378900	0.85779700
H	-0.28196800	-2.09137400	0.90491400
C	-2.57912500	1.30346500	-1.54677200
C	4.37259300	-0.70299000	-1.70472900
H	3.91754100	-0.33354400	-0.77586200
H	5.46011800	-0.50287300	-1.64605700
H	4.24158700	-1.79404100	-1.71428600
C	-0.19964600	-2.25648900	-3.32089600
H	-0.44422800	-2.10573700	-2.25802400
H	-0.51807000	-3.28155000	-3.59299900
H	-0.83253200	-1.56047900	-3.89566200
C	2.12214000	-3.11356800	-2.86794700
H	3.17102200	-3.12691100	-3.20097000
H	1.71669500	-4.12826600	-3.04571600
H	2.11933500	-2.93557900	-1.78362300
C	4.47206000	-0.57825100	-4.20445300
H	4.38453300	-1.67192100	-4.28546800
H	5.55359800	-0.34263500	-4.17307300
H	4.07272300	-0.14012900	-5.13347300
C	-2.40815000	1.94558800	-3.92772100
H	-1.90806800	1.82455200	-4.89329100

C	2.26016900	4.04806000	0.33794200	H	-6.03899700	-4.10180300	-0.45706600
C	3.16083100	0.83307200	1.84554700	H	-4.99576600	-5.12703600	-1.46125300
H	2.86553100	-0.03838400	1.25373600	H	-5.61446100	-3.60034400	-2.10632300
C	2.92048000	3.33734500	1.30730200	C	-4.65989500	1.50866600	2.09227500
C	1.46348200	4.21162800	-2.71230200	H	-4.92726500	2.24154200	1.31777900
H	1.56987400	5.26892300	-2.43376200	H	-5.04846200	1.89447700	3.05057300
H	0.86339300	4.17824100	-3.63362300	H	-5.20675000	0.58216100	1.86513900
H	2.45674600	3.80097200	-2.93487900	C	-2.85152900	-3.98115000	-0.20416800
C	1.49594800	-2.26413300	-5.14096100	C	-2.52547400	-5.43817100	-0.12636400
H	0.88011400	-1.57232500	-5.73690400	H	-3.42557300	-6.03346700	-0.32790800
H	1.19615500	-3.29141300	-5.42552200	H	-2.15359400	-5.73195200	0.86443600
H	2.54149100	-2.13291400	-5.45555100	H	-1.76094800	-5.72458200	-0.86542800
C	-3.46217800	2.85440100	-3.79242600	C	-3.99875200	-3.40505200	-0.68320700
H	-3.79568800	3.45027000	-4.64671400	C	-2.68412700	4.00954400	1.71961300
N	-1.99893900	-2.93768900	0.15464300	H	-2.66023800	3.70425700	0.66488200
C	-2.63126200	-0.08511500	3.69271200	H	-2.16483100	4.98413900	1.79297200
C	-3.19310800	0.40832900	5.04161000	H	-3.73892400	4.18435500	1.98337900
H	-2.56324700	1.17573700	5.50836500	C	-5.03024400	-0.81485400	-2.41095900
H	-3.25111400	-0.43573900	5.75556800	H	-4.06738500	-0.66473600	-2.91728000
H	-4.21038300	0.82083700	4.94669200	H	-5.63761700	0.08508000	-2.58863700
C	-2.57541800	-1.72619300	-0.10616900	H	-5.54790600	-1.67274600	-2.86653300
C	-1.98550400	1.19503200	-2.82346200	C	-4.08317100	2.98873000	-2.54623200
H	-1.15107200	0.50563800	-2.96323100	H	-4.90951800	3.69514500	-2.41997700
C	1.30077300	-2.05438700	-3.62623900	C	2.45830800	0.70044900	3.19632700
C	-0.87837800	-3.62138800	2.28796200	H	2.61822200	1.57314500	3.84735000
H	-1.18872100	-4.67679400	2.30960100	H	2.83662700	-0.18951700	3.71788000
H	-1.61356200	-3.03616700	2.84595000	H	1.37947900	0.56592300	3.05058700
H	0.08701600	-3.54417000	2.80647300	C	-0.59322600	4.01093100	-1.23893100
C	-2.02808300	2.97913500	2.66425300	H	-1.09499100	3.38728100	-0.49568600
C	-5.22611700	-4.08542000	-1.20085200	H	-1.25070700	4.05714000	-2.11887800

H	-0.48458900	5.02602300	-0.82948000
C	-4.82359600	-0.99295200	-0.90731200
H	-4.37284400	-0.07148800	-0.52776400
C	-0.50389800	3.00230500	2.45880900
H	0.01180900	2.32702200	3.15768100
H	-0.09656100	4.01768100	2.62771300
H	-0.21565200	2.69548900	1.44513400
C	4.68818300	0.82509400	1.94135100
H	5.14378800	1.01053900	0.95696200
H	5.01369100	-0.16944700	2.27759900
H	5.07936000	1.56053600	2.65779800
C	-6.12813000	-1.20103500	-0.13228600
H	-6.76614500	-1.98200200	-0.56879100
H	-6.70085500	-0.26175300	-0.14673500
H	-5.92544100	-1.45756800	0.91820400
C	3.78173000	3.86714100	2.40768300
H	3.58439000	3.36326100	3.36340400
H	3.58477900	4.93806300	2.55267800
H	4.85566000	3.75592000	2.18732800
C	-3.47861000	-1.31403100	3.29554900
H	-4.55390600	-1.07485100	3.29729500
H	-3.33516400	-2.13648100	4.02160300
H	-3.23231200	-1.70070900	2.29744800
C	2.23961200	5.52841200	0.13648200
H	2.88638900	5.84371800	-0.69816200
H	2.60333700	6.03300100	1.04165400
H	1.22689700	5.90254800	-0.06567300
C	-2.32111300	3.43646500	4.10499400
H	-3.38685100	3.34648800	4.36952800
H	-2.04111400	4.50056700	4.22804100
H	-1.74048900	2.86713000	4.84531200
C	3.54306900	-2.68936500	2.61225200
C	4.39319900	-2.82663900	3.70703400
C	5.76694400	-2.95372000	3.47399600
C	6.26551500	-2.94284000	2.16647500
C	5.38572300	-2.80212800	1.08692400
C	4.01053900	-2.67215100	1.30112700
H	3.97381800	-2.83007400	4.71498800
H	6.44881800	-3.06047900	4.32136600
H	7.33929000	-3.03882400	1.98979100
H	5.76824300	-2.77337800	0.06423500
H	3.30359100	-2.50213100	0.49057000
F	2.22117300	-2.54022800	2.83907700

TS2a

B3LYP(D3)/def2-SVP

E = -3517.762852 a.u.

Si	-2.60965800	-2.61389900	0.42285000
Al	-1.53520200	-0.43063800	-0.42678800
Al	1.53194100	0.24044100	0.21661400
Si	2.46620400	2.62836900	0.09422600
N	-3.30548300	1.31005300	1.57532200
F	-2.45466400	-0.60930700	-1.90705400
N	-3.71667100	1.76302200	-0.50214600
N	4.29053200	-1.10792100	1.08253800
C	-2.82672300	1.12782000	0.31188300
C	-2.32912600	-3.34229900	2.17656200
H	-1.33024200	-3.79900400	2.24780500
H	-3.06588700	-4.13464700	2.39115900
H	-2.39552700	-2.59156700	2.97499100

C	-4.59020600	-2.55396300	0.24809100
C	-5.18125800	-1.84922900	1.48485800
H	-4.95160700	-2.37911800	2.42311400
H	-6.28427300	-1.79880600	1.40357500
H	-4.81782300	-0.81563300	1.57401800
C	2.76821600	-3.87779800	-2.19305900
H	3.65084000	-4.32676400	-2.66621200
H	1.91485800	-4.06339400	-2.86113500
H	2.56752500	-4.39512100	-1.24411000
C	-2.49430800	1.02963000	2.78604000
H	-1.58474000	0.55356100	2.39766900
C	3.15255800	2.76712300	-2.78597000
H	2.62611100	1.81533500	-2.93368400
H	3.95008600	2.82947900	-3.55095800
H	2.44363400	3.57788300	-3.00104300
C	1.00959200	0.76593600	3.15616400
H	1.29255600	1.79715000	2.92947300
C	2.89642900	-2.36556700	-1.98948300
H	1.89859400	-1.99670500	-1.75634500
C	0.95392700	-0.18738600	2.11591200
C	-5.04927100	-1.76359300	-0.98944300
H	-4.74974600	-0.71099200	-0.91896100
H	-6.15313900	-1.79805000	-1.07434000
H	-4.62314000	-2.15034400	-1.92511600
C	-0.31979800	-3.87060200	-0.69377000
H	0.01914400	-2.86941400	-1.00370400
H	0.15579500	-4.61263000	-1.35896800
H	0.07935600	-4.04555300	0.31958600
C	-2.30820500	-3.83508600	-2.21149800
H	-3.37769300	-4.06803900	-2.33255200
H	-1.75266300	-4.52351100	-2.87863300
H	-2.15663200	-2.80645300	-2.56797700
C	-5.20298200	-3.96579000	0.18047300
H	-4.93791500	-4.48696800	-0.75096200
H	-6.30798600	-3.90068300	0.20831300
H	-4.89603600	-4.60331400	1.02535000
C	0.26578000	-1.82798300	3.82141100
H	-0.03538800	-2.84840300	4.06972900
C	-4.52841900	1.98414400	1.54943200
C	-3.43327200	2.04700200	-1.93313800
H	-2.50494500	1.51030600	-2.13279500
C	-4.78718600	2.26936300	0.23379000
C	-3.14141300	0.05978700	3.77373300
H	-4.00666800	0.49782100	4.29094800
H	-2.39068200	-0.20500700	4.53176200
H	-3.45853700	-0.86230800	3.27928000
C	-2.17568300	-5.45407100	-0.29668300
H	-1.84530700	-5.65028000	0.73570200
H	-1.65166500	-6.18126600	-0.94732000
H	-3.24642800	-5.68808800	-0.35329400
C	0.36422700	-0.86443700	4.83100500
H	0.14946300	-1.12710700	5.87044000
N	3.68937600	-2.00078400	-0.79187900
C	3.76739000	2.87512400	-1.37759300
C	4.48611000	4.23442000	-1.25320400
H	3.78833400	5.08400800	-1.31154000
H	5.21708100	4.35832500	-2.07501000
H	5.04422000	4.32703800	-0.30863000
C	3.27681800	-1.11011700	0.16150500
C	0.55584300	-1.48828400	2.49547400

H	0.47625700	-2.26629100	1.73166300
C	-1.85188700	-4.01547900	-0.75096700
C	3.39360200	-1.64376000	-3.24265200
H	4.38031700	-2.01112900	-3.56432800
H	3.45320200	-0.56184700	-3.07423000
H	2.68091800	-1.81163700	-4.06395000
C	1.01211800	4.00520500	0.10415300
C	6.57616900	-2.23200300	1.47100600
H	7.29082400	-1.39710700	1.40351900
H	7.07139500	-3.12683300	1.07209600
H	6.38006600	-2.41798800	2.53571000
C	3.54384300	3.07930600	1.62608300
H	3.07479200	2.81106800	2.58437000
H	3.76817800	4.15558200	1.66061000
H	4.50600000	2.55740000	1.57344700
C	4.94658100	-2.51917800	-0.49086000
C	5.72728400	-3.48095400	-1.33028600
H	6.78217300	-3.46573300	-1.02613200
H	5.69389100	-3.21657400	-2.39563800
H	5.36677600	-4.51686100	-1.23097400
C	5.31790700	-1.96918900	0.70556900
C	1.23311800	5.02652500	1.23839900
H	1.29594200	4.54819900	2.22848500
H	0.38691700	5.73879200	1.27449400
H	2.15032100	5.61929000	1.09463400
C	3.89215300	-1.23701200	3.53438000
H	2.95829400	-1.77710800	3.34143400
H	3.73830800	-0.61437400	4.42795100
H	4.68665700	-1.96562300	3.75862300
C	0.73495900	0.43989900	4.49023100
H	0.81281900	1.20728400	5.26683000
C	-3.16120900	3.53853800	-2.14945500
H	-4.06039700	4.16143000	-2.03279000
H	-2.78849800	3.68554400	-3.17409300
H	-2.39296100	3.90462800	-1.45482100
C	-2.08838500	2.33966200	3.47228500
H	-1.64793200	3.04684800	2.75936400
H	-1.34153200	2.12077900	4.24622800
H	-2.94300700	2.82903300	3.96101000
C	4.26260600	-0.34128300	2.35165700
H	3.45600500	0.38306900	2.20862300
C	-0.30880200	3.27276700	0.38673300
H	-0.59183700	2.58843300	-0.42423800
H	-1.14268800	3.98878700	0.51967100
H	-0.25258100	2.66266800	1.29867800
C	-4.46622600	1.47104000	-2.89823500
H	-4.59602000	0.39838700	-2.71518300
H	-4.07531200	1.58313700	-3.92113100
H	-5.43876300	1.98249300	-2.85173600
C	5.55135400	0.44594000	2.61825300
H	6.33588300	-0.16917900	3.07741200
H	5.32408900	1.25893200	3.32191100
H	5.95209500	0.89635400	1.69961600
C	-6.01169300	2.89178500	-0.35433000
H	-5.77663600	3.68798200	-1.07326300
H	-6.63016800	3.33284400	0.43851700
H	-6.62645300	2.14307300	-0.87980900
C	4.82914800	1.76040900	-1.25683800
H	5.35136100	1.77576600	-0.28758300
H	5.60137800	1.88132700	-2.03981900

H	4.39026100	0.75959500	-1.38200400
C	-5.41530300	2.20740500	2.73143400
H	-5.83876800	1.25745300	3.09656600
H	-6.25510700	2.85742600	2.45268700
H	-4.89451600	2.68346400	3.57298800
C	0.84694800	4.80415900	-1.20236100
H	1.74070000	5.39538500	-1.45099600
H	0.00444600	5.51527400	-1.10385600
H	0.62182200	4.16668000	-2.06870400
C	0.15442700	0.16824100	-2.48722800
C	-0.11809800	1.45803600	-2.98291100
C	-0.77512400	1.60698300	-4.20316800
C	-1.11026500	0.49134900	-4.98076500
C	-0.74499500	-0.78250800	-4.52509600
C	-0.07459300	-0.95031900	-3.31742500
H	0.16081200	2.32968000	-2.39682600
H	-1.01536000	2.61474200	-4.55371900
H	-1.62682800	0.61318900	-5.93507800
H	-0.98659900	-1.66795700	-5.11939000
H	0.17601600	-1.95516100	-2.97990600
F	1.54116800	0.11695300	-1.77526100

INT3

B3LYP(D3)/def2-SVP

E = -3517.978018 a.u.

Si	-4.33354900	-1.97769000	0.82171600
Al	-3.86611000	0.08116100	-0.49522700
Al	2.48101900	0.07151900	0.47243400
Si	3.53291200	2.22407300	-0.16091400
N	-2.55098400	2.04653300	1.61076400
F	-5.40139000	0.74024000	-0.94972100
N	-3.02497100	2.99346300	-0.27327700
N	4.64559500	-2.04722200	0.04903400
C	-3.04222700	1.79838800	0.37222600
C	-4.63387700	-1.78028400	2.70808400
H	-3.68480500	-1.72759600	3.26492700
H	-5.19414500	-2.64261500	3.10634100
H	-5.21048500	-0.87144300	2.94026400
C	-6.03287900	-2.65183700	0.10454100
C	-7.14481400	-1.72165100	0.63792900
H	-7.24762200	-1.78128300	1.73352800
H	-8.12124800	-2.01063200	0.20435900
H	-6.95809200	-0.67195600	0.35976700
C	0.73821700	-3.09136300	-1.85709900
H	0.95097000	-4.12174200	-2.18192600
H	-0.19333000	-2.76373900	-2.34085200
H	0.56247400	-3.08975500	-0.77389300
C	-2.34704300	0.96764300	2.60332400
H	-2.81912600	0.09904500	2.12942400
C	2.99183500	1.62427800	-2.94051100
H	2.67821000	0.62137200	-2.61631900
H	3.34296800	1.53545400	-3.98620300
H	2.09370700	2.25801700	-2.95058000
C	2.97452100	0.29617900	3.45270600
H	3.50817600	1.22732000	3.23315000
C	1.85264200	-2.11340200	-2.21888200
H	1.51020700	-1.12599400	-1.89174300
C	2.39852700	-0.44076800	2.39899600
C	-6.06154500	-2.58093800	-1.43641500
H	-5.99446300	-1.53923600	-1.78715800

H	-7.01644000	-2.98916700	-1.81885800	N	3.08753800	-2.35354000	-1.42272300
H	-5.24903100	-3.15726000	-1.90503300	C	4.10430500	2.19117000	-2.03905200
C	-1.56188300	-2.50713300	1.07537100	C	4.51637000	3.57703900	-2.56614000
H	-1.31205800	-1.68483900	0.38563000	H	3.65686100	4.26154100	-2.63230400
H	-0.70301800	-3.20497300	1.06938500	H	4.94211400	3.49365000	-3.58428900
H	-1.61116200	-2.08929300	2.09336500	H	5.27979300	4.05632400	-1.93154000
C	-2.70877900	-3.78579200	-0.74874100	C	3.46712200	-1.54287700	-0.40419200
H	-3.56864700	-4.40705600	-1.04446700	C	1.72793700	-1.63061800	2.75719400
H	-1.80582400	-4.41917900	-0.84003800	H	1.25319300	-2.23894400	1.98024400
H	-2.61581500	-2.96823900	-1.48132300	C	-2.85362900	-3.25662400	0.68978600
C	-6.33443600	-4.09492400	0.54568300	C	2.13841100	-2.04082100	-3.71941900
H	-5.63742700	-4.81873800	0.09484900	H	2.32726400	-3.02605500	-4.16643600
H	-7.35384000	-4.38775800	0.22910200	H	2.99556100	-1.38421100	-3.93174200
H	-6.28706900	-4.21926600	1.64024800	H	1.25358600	-1.62123000	-4.21675800
C	1.63218300	-2.06164600	4.08477500	C	2.25055700	3.65303200	0.23577900
H	1.09859200	-2.98649900	4.32396600	C	6.24223200	-3.99252000	-0.45790700
C	-2.22947400	3.39462700	1.75618000	H	7.12720300	-3.49966200	-0.89246500
C	-3.51579500	3.14918700	-1.66578100	H	6.14173600	-4.97161100	-0.94526500
H	-3.63766400	2.12109600	-2.02234800	H	6.44953100	-4.17161200	0.60563500
C	-2.53933700	3.99731400	0.56156100	C	5.10411800	2.63593300	0.85737400
C	-3.09095500	1.22757800	3.91341300	H	4.84389400	2.85083600	1.90651600
H	-2.61438900	2.00854600	4.52216000	H	5.63214000	3.51901600	0.45962200
H	-3.09607600	0.30299400	4.50862000	H	5.81846600	1.79752100	0.86262100
H	-4.13709500	1.51202900	3.72390100	C	4.00672000	-3.38770700	-1.59494600
C	-3.01214800	-4.44778800	1.65495100	C	3.90677400	-4.47670000	-2.61396400
H	-3.08254200	-4.12236600	2.70546100	H	4.56316500	-5.31201200	-2.33436600
H	-2.13424900	-5.11884800	1.58169700	H	4.21301900	-4.13718600	-3.61632800
H	-3.90260500	-5.05358800	1.43473500	H	2.88457100	-4.87018700	-2.69339300
C	2.21761100	-1.30551600	5.10712700	C	4.99827800	-3.19032300	-0.66480900
H	2.14754600	-1.63636000	6.14716600	C	2.88127500	5.05731900	0.18778200

H	3.71969300	5.15541900	0.89638700
H	2.13149100	5.82397600	0.46343100
H	3.25848600	5.31770700	-0.81148200
C	5.34400400	-2.26475600	2.43899800
H	4.30695400	-2.52243500	2.69283200
H	5.75528800	-1.68028400	3.27546400
H	5.93506900	-3.18849600	2.34987500
C	2.89099000	-0.12234800	4.78717900
H	3.34971000	0.47628200	5.58002500
C	-2.47775400	3.80136100	-2.58024800
H	-2.39328100	4.88733800	-2.43390500
H	-2.77552000	3.62970000	-3.62550000
H	-1.48957200	3.34058800	-2.43528700
C	-0.86673900	0.64353100	2.78408900
H	-0.39084200	0.44122700	1.81550900
H	-0.75334400	-0.25540900	3.40614200
H	-0.31372900	1.45132100	3.28418900
C	5.39395100	-1.42045400	1.16528100
H	4.83832500	-0.49657700	1.37165400
C	1.72655900	3.41576200	1.66752500
H	1.17620900	2.46700600	1.75738100
H	1.03072600	4.22499000	1.95567400
H	2.53527700	3.40350700	2.41630300
C	-4.89746900	3.80095200	-1.69820100
H	-5.60297400	3.19269000	-1.11533600
H	-5.25764100	3.83278600	-2.73812000
H	-4.88738500	4.83254100	-1.31321600
C	6.80993100	-1.00668600	0.75889000
H	7.48740500	-1.86545600	0.65335500
H	7.22670600	-0.35322700	1.54007900
H	6.80084400	-0.44347800	-0.18510700
C	-2.40000600	5.43871500	0.19160900
H	-1.55336500	5.61071700	-0.49116500
H	-2.22662400	6.04146400	1.09282100
H	-3.30483400	5.82635100	-0.29692900
C	5.32128600	1.24721300	-2.13640800
H	6.18622200	1.62304100	-1.56657400
H	5.64486200	1.14274300	-3.18926000
H	5.08523600	0.23558900	-1.76726200
C	-1.69404000	4.02485800	3.00100000
H	-2.47186300	4.13603400	3.77378200
H	-1.30093200	5.02471900	2.77579400
H	-0.87070900	3.44230300	3.43430400
C	1.04935900	3.59143300	-0.72666000
H	1.32476300	3.88110800	-1.75270700
H	0.25827200	4.28773500	-0.39506000
H	0.60324000	2.58457100	-0.76558500
C	-2.75332300	-0.16159200	-2.14386200
C	-1.41976900	0.29642700	-2.20847100
C	-0.66376600	0.22379900	-3.38410500
C	-1.21978800	-0.33167800	-4.54173400
C	-2.53165800	-0.81720200	-4.50638300
C	-3.27983600	-0.72667600	-3.32619400
H	-0.94250000	0.71981800	-1.32164700
H	0.35898900	0.60778900	-3.39418400
H	-0.63736600	-0.38183400	-5.46675500
H	-2.97654400	-1.25666000	-5.40445100
H	-4.30807700	-1.10155800	-3.33513600
F	0.87195000	-0.01066200	-0.16231000

3

B3LYP(D3)/def2-SVP

E = -1758.972748 a.u.

Al	-0.38766600	0.46060700	-0.82482900
Si	-2.28106900	-0.44880800	0.48913500
F	-0.47202200	-0.13661100	-2.44148000
C	-0.03214800	2.42196100	-0.80215600
N	1.99840700	-0.00579700	1.06399100
N	2.26803900	-1.22014600	-0.70523400
C	0.68443200	5.17661400	-0.67668600
H	0.96111400	6.23354700	-0.62678500
C	-0.34539000	4.67813500	0.12742600
H	-0.87975800	5.34765900	0.80828000
C	-0.69272800	3.32342700	0.05760900
H	-1.50216200	2.96134000	0.70105200
C	1.42265600	-0.33275300	-0.12273500
C	3.21222900	-0.67161400	1.22553600
C	4.10344200	-0.56759700	2.42067700
H	4.25141900	0.47422100	2.73693200
H	5.09183500	-0.98659300	2.18905800
H	3.70335300	-1.12474500	3.28310800
C	0.99608300	2.96070500	-1.60588100
H	1.54141900	2.30657000	-2.29676600
C	1.35398500	4.31272800	-1.55070400
H	2.15627700	4.69583900	-2.18874700
C	3.38410000	-1.44192200	0.10206100
C	2.01945400	-1.77645200	-2.05928600
H	0.98899100	-1.48044700	-2.28376700
C	2.08581400	-3.30463400	-2.09781600
H	3.11285700	-3.69520500	-2.08164500

H	1.61770300	-3.65235000	-3.03115600
H	1.52537100	-3.74528600	-1.25983600
C	2.90814300	-1.09330600	-3.10050500
H	2.77041300	-0.00227200	-3.06294500
H	2.62238200	-1.43547800	-4.10681600
H	3.97702000	-1.31695300	-2.95790500
C	4.50976700	-2.37201000	-0.21619900
H	4.22218600	-3.42779700	-0.08719600
H	5.35761700	-2.17772900	0.45434300
H	4.86847800	-2.24958000	-1.24763700
C	1.39373900	0.98964700	1.98167900
H	0.39131000	1.15720200	1.56711000
C	2.13667100	2.32507200	1.92820900
H	3.14781300	2.25137400	2.35721100
H	1.57660300	3.07636900	2.50433000
H	2.20894200	2.68700900	0.89347900
C	1.20282800	0.45255300	3.40172500
H	0.74217000	-0.54535500	3.38957100
H	0.52830900	1.12990200	3.94641000
H	2.14303100	0.40391900	3.96780400
C	-1.49683400	-3.06375600	-0.47992900
H	-2.22517800	-3.01132700	-1.30175900
H	-1.29167200	-4.13423400	-0.28593700
H	-0.56326800	-2.61240500	-0.84862500
C	-1.99860600	-2.36887100	0.80092700
C	-3.26586500	-3.08660300	1.29801300
H	-3.69273800	-2.60583700	2.19359100
H	-3.03781700	-4.13596300	1.56696500
H	-4.05053200	-3.11467900	0.52682800
C	-0.90269700	-2.51288300	1.87773300

H	0.03149500	-2.00763800	1.58250500
H	-0.65888200	-3.58021500	2.03931500
H	-1.21791800	-2.10131700	2.85001500
C	-3.92571500	-0.04698900	-0.49683100
C	-3.99554400	-0.87620600	-1.79336400
H	-3.07629700	-0.77632100	-2.39385700
H	-4.84350900	-0.54121200	-2.42028000
H	-4.15249500	-1.94719200	-1.58820100
C	-5.19870900	-0.28275100	0.33829000
H	-5.31993800	-1.33428300	0.63679200
H	-6.09655200	-0.00370600	-0.24555100
H	-5.20763600	0.32743800	1.25610100
C	-3.87567900	1.44850300	-0.87954100
H	-3.80135400	2.10424100	0.00392200
H	-4.79689100	1.73663100	-1.42005600
H	-3.02492900	1.68635500	-1.53862300
C	-2.51950900	0.33528700	2.22247500
H	-1.59127900	0.33130300	2.81367900
H	-3.28639400	-0.19971400	2.80786500
H	-2.84671600	1.38371500	2.13038600

TS2b1

B3LYP(D3)/def2-SVP

E = -3186.520662 a.u.

Si	2.58344100	2.37607100	0.20285000
Al	1.21426900	0.25232900	-0.19975400
Al	-1.23720500	-0.42751700	-0.10502000
Si	-2.89645900	-2.34548200	0.19843700
N	3.52228600	-1.63274900	0.86667400
F	1.38925900	0.57555500	-1.95385600
N	3.06371000	-1.96949000	-1.22278000
N	-3.35466900	1.81273000	0.70037300
C	2.60219000	-1.34824800	-0.10091000
C	3.01944000	2.90226000	2.00422200
H	2.16694800	3.39553000	2.49694900
H	3.86253500	3.61346700	2.02925700
H	3.28853000	2.03593400	2.62871400
C	4.36037600	2.28525400	-0.68741200
C	5.30348800	1.43941100	0.18990800
H	5.47044400	1.88924500	1.18160100
H	6.29532200	1.34485600	-0.29337400
H	4.91617900	0.42104000	0.33227500
C	-1.56577800	2.76022800	-3.39121400
H	-2.35901600	3.09919400	-4.07249600
H	-0.68292000	2.51980500	-4.00067200
H	-1.28558900	3.58503400	-2.72312100
C	3.26832200	-1.34949100	2.29921200
H	2.42664100	-0.64922000	2.27467300
C	-4.73023200	-2.65432300	-2.12249500
H	-4.00109000	-2.12642600	-2.75497100
H	-5.73078600	-2.46127000	-2.55391900
H	-4.54183400	-3.73255900	-2.22285700
C	-0.09478300	-1.17455800	2.54019100
H	-0.06063200	-2.10592200	1.97009200
C	-1.95063600	1.51149100	-2.59685000
H	-1.01424300	1.08651200	-2.19615700
C	0.22981600	0.04052100	1.89434800
C	4.28854500	1.59835700	-2.06265400
H	3.93719600	0.56334400	-1.96788200
H	5.29220800	1.57550600	-2.53138300

H	3.60347400	2.10232700	-2.75766100
C	0.09908200	3.78537700	-0.03319000
H	-0.35767400	2.83142500	-0.33605500
H	-0.52694100	4.60011800	-0.44767900
H	0.02270200	3.85576800	1.06465800
C	1.55751500	3.88250600	-2.05555200
H	2.55395400	4.09985100	-2.46829100
H	0.87074700	4.65065200	-2.46193000
H	1.24750100	2.89762900	-2.43527600
C	5.01131200	3.67094000	-0.85603500
H	4.46695500	4.30377300	-1.57171200
H	6.04309800	3.56293500	-1.24376500
H	5.08078300	4.22136000	0.09639100
C	-0.12220800	1.12815100	4.08494800
H	-0.11209300	2.04209600	4.68697800
C	4.57526700	-2.38275600	0.34618500
C	2.22260500	-2.15258400	-2.43622900
H	1.29844300	-1.61175600	-2.20100000
C	4.29420600	-2.58211800	-0.98120500
C	4.41150600	-0.66163500	3.04799800
H	5.23450600	-1.34407600	3.30174900
H	4.00782800	-0.27053600	3.99426200
H	4.81509200	0.18565200	2.48370400
C	2.07612200	5.28291100	-0.03240800
H	2.06241700	5.36480300	1.06640700
H	1.43524500	6.09639900	-0.42615400
H	3.10129400	5.49132900	-0.36650800
C	-0.41762200	-0.10297800	4.68746400
H	-0.64671400	-0.15710700	5.75547000
N	-2.75602500	1.81386300	-1.38317600
C	-4.69359300	-2.18264000	-0.65623900
C	-5.72741300	-3.01454200	0.13363900
H	-5.45009700	-4.07706600	0.20680800
H	-6.71305100	-2.96644600	-0.36738800
H	-5.86767400	-2.63689500	1.15809000
C	-2.48760900	1.24013500	-0.17626100
C	0.16199000	1.18858400	2.71886200
H	0.37074700	2.16525300	2.28132900
C	1.55671500	3.91482400	-0.51645600
C	-2.61122600	0.43751000	-3.45896700
H	-3.60226900	0.74821400	-3.82599200
H	-2.73082200	-0.49882000	-2.89631700
H	-1.97233800	0.22449400	-4.32952500
C	-1.97914400	-3.96664400	-0.45162800
C	-5.33757400	3.44129400	0.67611900
H	-6.15069700	2.75890500	0.97225700
H	-5.75533100	4.16856300	-0.03204000
H	-5.02913100	3.99777600	1.57187000
C	-3.27759900	-2.69417200	2.04470900
H	-2.35148300	-2.78158800	2.62762400
H	-3.85690300	-3.62145700	2.18217000
H	-3.85683700	-1.87052400	2.49102800
C	-3.83137500	2.69449100	-1.27884500
C	-4.44904600	3.43690700	-2.41919400
H	-3.83675600	4.29262500	-2.74343800
H	-5.43425900	3.82275700	-2.12553300
H	-4.59800700	2.78500000	-3.29208300
C	-4.20302900	2.70308500	0.04261800
C	-2.85065500	-5.22733400	-0.29317000
H	-3.16259300	-5.38656300	0.75170700

H	-2.28661600	-6.12721400	-0.60487300
H	-3.76017200	-5.18946300	-0.91094900
C	-2.74898700	2.85420700	2.86393800
H	-1.83565900	3.22686300	2.38208000
H	-2.50996500	2.61818400	3.90997200
H	-3.50080200	3.65699800	2.86058400
C	-0.41497600	-1.25793100	3.90390100
H	-0.65008000	-2.22721800	4.35408500
C	1.86998600	-3.63107100	-2.62346200
H	2.72678400	-4.23915400	-2.94747700
H	1.09275500	-3.72037800	-3.39592200
H	1.46910700	-4.05867800	-1.69396200
C	2.80712300	-2.62775700	3.00659000
H	1.99303300	-3.11177000	2.44995700
H	2.43077400	-2.37980900	4.00988400
H	3.63029000	-3.35147700	3.11677600
C	-3.25403800	1.59105300	2.16171200
H	-2.47540800	0.82260200	2.26030100
C	-0.69334400	-4.16806900	0.37787500
H	0.00882900	-3.32743400	0.25304000
H	-0.16557400	-5.08249300	0.04619400
H	-0.89942000	-4.28760900	1.45367500
C	2.80708500	-1.49929300	-3.68578500
H	2.91447400	-0.42062900	-3.52331900
H	2.10177700	-1.64459300	-4.51910000
H	3.77088300	-1.93440800	-3.98800600
C	-4.53142100	1.04251100	2.79945200
H	-5.33749200	1.78839400	2.84390400
H	-4.30203100	0.73887500	3.83184600
H	-4.90127300	0.15750500	2.26568800
C	5.16845800	-3.20514300	-2.02146500
H	4.63917100	-3.93752900	-2.64467200
H	6.01101900	-3.72316800	-1.54446600
H	5.58878000	-2.44076300	-2.69535000
C	-5.16415700	-0.71593600	-0.63877300
H	-5.13332200	-0.27685500	0.36705200
H	-6.20935200	-0.64441600	-0.99550900
H	-4.55350800	-0.07858000	-1.29210400
C	5.79029200	-2.79555800	1.11074000
H	6.42064200	-1.93001100	1.37011300
H	6.39944500	-3.48167700	0.50769500
H	5.53642400	-3.31462300	2.04623900
C	-1.55379900	-3.82120200	-1.92578300
H	-2.40794400	-3.76086500	-2.61216500
H	-0.95197800	-4.69457800	-2.23757700
H	-0.93382800	-2.92238700	-2.07807800

INT4

B3LYP(D3)/def2-SVP

E = -3186.552567 a.u.

Si	-4.52200500	-1.04286500	-0.26447500
Al	-2.06436600	-0.31473800	-0.03253900
Al	2.46441400	0.30932200	-0.07871400
Si	4.93723400	0.09094600	-0.17578900
N	-2.47549200	2.74626000	0.48668300
F	1.81601800	0.50287200	-1.67827800
N	-2.03491500	2.36646700	-1.59588400
N	1.01247300	-1.72234700	1.78204900
C	-2.31948400	1.74401400	-0.42030900
C	-5.90919100	0.26029000	-0.52523000

H	-6.06387500	0.84417400	0.39679500	H	-4.94246600	0.03539400	2.50604600
H	-6.88042400	-0.19158400	-0.79174600	C	-4.21985600	-3.24617600	1.61199400
H	-5.63752200	0.97508000	-1.31852500	H	-4.60194600	-4.01469700	0.92253200
C	-4.69730000	-2.29120000	-1.78136200	H	-4.34063700	-3.64344900	2.63864100
C	-4.65600700	-1.44268500	-3.06792700	H	-3.13927200	-3.13186900	1.42447800
H	-5.49637300	-0.73331800	-3.13044700	C	-6.01466800	-3.08640400	-1.76115200
H	-4.70489200	-2.09006900	-3.96411600	H	-6.05610600	-3.79306700	-0.91791300
H	-3.72257000	-0.86124600	-3.14002800	H	-6.12398600	-3.68269200	-2.68780900
C	0.42989300	-2.07175600	-2.62702300	H	-6.89698900	-2.42957900	-1.69130400
H	-0.43667600	-2.74891600	-2.61276500	C	0.98618200	4.23838600	0.63451200
H	0.82901400	-2.04611400	-3.65293300	H	0.43447700	4.95463800	0.02078500
H	0.10506100	-1.05938500	-2.35689200	C	-2.30272800	3.99663800	-0.11465000
C	-2.79295800	2.45067000	1.89502200	C	-1.89253200	1.58969200	-2.84267700
H	-2.69802900	1.35490900	1.95918500	H	-1.92234200	0.53993900	-2.50105300
C	5.38265500	-2.77164600	-0.10634300	C	-2.01459500	3.75641500	-1.43304700
H	4.31691000	-2.86196300	-0.37144100	C	-4.24169000	2.80441800	2.23706400
H	5.65941400	-3.67984600	0.46293100	H	-4.40478700	3.89261800	2.24041400
H	5.96206600	-2.78635400	-1.04238800	H	-4.49158600	2.42646400	3.24014700
C	2.43748900	2.40966300	2.14042400	H	-4.93305000	2.35121800	1.51310100
H	3.05476500	1.73189100	2.74271500	C	-6.47573400	-2.13878300	1.64326200
C	1.53281000	-2.52942000	-1.67464200	H	-7.04290600	-1.19521500	1.60070400
H	2.34902500	-1.80350200	-1.75345300	H	-6.67928900	-2.59680400	2.63088500
C	1.94433600	1.98512700	0.88805000	H	-6.89494200	-2.81052100	0.88003900
C	-3.51621600	-3.27918900	-1.83787700	C	1.47678500	4.61916000	1.88678100
H	-2.55075600	-2.75079100	-1.89144400	H	1.30809100	5.63184200	2.26441800
H	-3.59155000	-3.92549000	-2.73370800	N	1.11927300	-2.44773900	-0.25252300
H	-3.47471100	-3.94012900	-0.96043900	C	5.65063100	-1.50322400	0.72120100
C	-4.48329400	-0.96349000	2.57740700	C	7.15893200	-1.39132200	1.01407300
H	-3.38544400	-0.83599500	2.56046100	H	7.75871700	-1.29711500	0.09724800
H	-4.74283400	-1.37077900	3.57355300	H	7.51637300	-2.29254800	1.54875000

H	7.38864100	-0.52278400	1.65163500
C	1.50328800	-1.42370200	0.55252700
C	1.20318200	2.94040800	0.15789000
H	0.80090100	2.66862700	-0.82153400
C	-4.96475800	-1.90817100	1.45577100
C	2.11519100	-3.89750000	-2.04140300
H	1.34020900	-4.65508100	-2.22131000
H	2.79552800	-4.26772400	-1.26032800
H	2.69501500	-3.79271200	-2.97093000
C	5.49896700	0.25488100	-2.04914000
C	-0.49751500	-3.46956900	2.89139000
H	0.17270900	-3.97349600	3.60613200
H	-1.21667900	-4.21588900	2.53024700
H	-1.06912600	-2.70762500	3.43992100
C	5.72548500	1.57156300	0.74836900
H	6.79928000	1.68207600	0.52145900
H	5.62497000	1.44805300	1.83929600
H	5.21883900	2.51309200	0.48592900
C	0.30923700	-3.33947400	0.44327500
C	-0.35194800	-4.54408700	-0.14214800
H	-0.78896200	-4.32772900	-1.12562000
H	-1.17319700	-4.86944500	0.51038200
H	0.34232800	-5.39220400	-0.25536500
C	0.24299100	-2.87997500	1.73635800
C	7.00169500	-0.00795400	-2.24962400
H	7.62644300	0.61645600	-1.58985800
H	7.29870000	0.21914800	-3.29160700
H	7.26519500	-1.06103400	-2.06291100
C	0.06351300	0.04047500	3.26403600
H	-0.24041500	0.58221800	2.35956700
H	0.34187700	0.77482800	4.03455900
H	-0.79780300	-0.54055500	3.62801100
C	2.19829700	3.69221200	2.64738900
H	2.59671200	3.97887000	3.62529400
C	-0.54126100	1.78686800	-3.53043500
H	-0.42846900	2.80059300	-3.94024800
H	-0.46731200	1.08314900	-4.37416300
H	0.29075900	1.56966400	-2.84552700
C	-1.77741800	3.04641900	2.87323600
H	-0.74794000	2.89032800	2.52434100
H	-1.89225800	2.55846000	3.85311100
H	-1.93048700	4.12437800	3.02278700
C	1.26231100	-0.85003500	2.94952100
H	2.07549300	-0.19670800	2.61297900
C	5.18568400	1.70430100	-2.48088800
H	4.12248200	1.95487700	-2.32887300
H	5.40320500	1.83840000	-3.55769100
H	5.79187100	2.44175700	-1.93129300
C	-3.09385900	1.80440000	-3.76695900
H	-4.03644800	1.63916300	-3.22551900
H	-3.05199700	1.09477800	-4.60689700
H	-3.10572900	2.82044300	-4.18888100
C	1.78773600	-1.61503400	4.16701600
H	0.99482100	-2.15875100	4.69828800
H	2.22093800	-0.89332000	4.87621600
H	2.57722300	-2.32612500	3.88303400
C	-1.65297400	4.73961300	-2.49767600
H	-0.57029400	4.72848300	-2.70720800
H	-1.91533400	5.75672300	-2.17663900
H	-2.17529800	4.54655100	-3.44527300

C	4.90767100	-1.64195900	2.06609900
H	4.99190500	-0.73473000	2.68834500
H	5.32158300	-2.48102500	2.65779100
H	3.83776000	-1.84800100	1.91371400
C	-2.39399600	5.30559500	0.59789000
H	-3.32025700	5.39315700	1.18464200
H	-2.38381200	6.13013600	-0.12748200
H	-1.54647800	5.45737900	1.28423400
C	4.69151300	-0.69096600	-2.96250900
H	4.81295500	-1.75061600	-2.68761400
H	5.02659200	-0.58826200	-4.01245300
H	3.61910200	-0.44508300	-2.93513600

INT5

B3LYP(D3)/def2-SVP

E = -1427.549183 a.u.

Si	1.88012400	-0.01077600	0.13775900
Al	0.27355400	-0.02158100	-1.89837300
N	-2.08281600	1.10128200	-0.13368400
N	-2.13271300	-1.05861700	-0.16277000
C	-1.39500300	0.01063800	-0.56918800
C	1.03651600	0.08862900	1.86285200
H	0.59119600	1.08703000	2.00856400
H	1.73269700	-0.08854000	2.70081500
H	0.21458800	-0.64113800	1.94169600
C	2.93436300	-1.67679400	0.16909600
C	1.99116500	-2.81425100	0.60852200
H	1.59301800	-2.66192800	1.62436200
H	2.52458100	-3.78372600	0.60674400
H	1.13420100	-2.92083300	-0.07621000
C	-1.61499500	2.46136000	-0.45650200
H	-0.59178700	2.30477700	-0.82763700
C	3.47214700	-2.02628300	-1.23258000
H	2.65990600	-2.12964600	-1.97137900
H	4.02002200	-2.98792300	-1.20706100
H	4.16612300	-1.26577700	-1.61901900
C	2.12251900	2.79791200	-0.26701000
H	1.64538000	2.70187600	-1.25818700
H	2.71158900	3.73474400	-0.28559600
H	1.32985800	2.93197200	0.48690600
C	4.07553900	1.46886900	-1.06762200
H	4.82814100	0.69638700	-0.84698000
H	4.62063400	2.42376300	-1.19540800
H	3.61332100	1.22027800	-2.03762000
C	4.11223200	-1.62028500	1.15880700
H	4.88019300	-0.89763100	0.84388300
H	4.60798800	-2.60793500	1.22836200
H	3.78957700	-1.34544300	2.17647200
C	-3.26165800	0.72420500	0.52077600
C	-1.73353800	-2.42999500	-0.52748500
H	-0.72028500	-2.30665200	-0.94251500
C	-3.29283200	-0.64522800	0.50434700
C	-1.49844800	3.36740500	0.77076800
H	-2.47576400	3.72340400	1.12541400
H	-0.89996400	4.25287400	0.50930500
H	-0.99186400	2.84585200	1.59608700
C	3.74186900	1.88262100	1.38857700
H	3.02628100	2.03830000	2.21189900
H	4.34971800	2.80463800	1.30707500
H	4.42084900	1.07192600	1.68919200

C	3.02863500	1.59109600	0.05392100
C	-2.61440700	-2.99232800	-1.64571300
H	-3.65106600	-3.14912000	-1.31100200
H	-2.21872400	-3.96339800	-1.98085800
H	-2.62152000	-2.30730700	-2.50671300
C	-2.43150300	3.07741500	-1.59507300
H	-2.43155800	2.40985100	-2.46974200
H	-1.98907300	4.03965000	-1.89565600
H	-3.47386500	3.26478400	-1.29441800
C	-1.60793700	-3.35837000	0.68145700
H	-1.02734600	-2.87979500	1.48316900
H	-1.07788300	-4.27505900	0.38227000
H	-2.58699600	-3.65790600	1.08079700
C	-4.30201200	-1.56048000	1.11804000
H	-4.61423800	-2.35888300	0.42925900
H	-5.20200600	-0.99589000	1.39678700
H	-3.91998700	-2.04280200	2.03288300
C	-4.23431500	1.66993500	1.14692200
H	-3.83850700	2.11902200	2.07260400
H	-5.15968100	1.13948900	1.40916400
H	-4.50700600	2.49305100	0.47020700

TS2b2

B3LYP(D3)/def2-SVP

E = -1758.781164 a.u.

Al	0.10747300	0.72525300	-0.71405600
C	-0.75812000	3.05133900	-0.35849500
C	-2.13631300	3.27828800	-0.55239800
C	0.17078100	4.09458100	-0.55048200
C	-2.54515500	4.47560700	-1.13333300
H	-2.85693300	2.49449200	-0.32063200
C	-0.26619800	5.28140800	-1.13314500
H	1.22067200	3.94131400	-0.30137300
C	-1.61930300	5.48284200	-1.44627400
H	-3.60838600	4.62646000	-1.34144200
H	0.46480900	6.06886000	-1.33744300
F	-0.42566800	2.12632900	0.77997200
H	-1.95033700	6.41855300	-1.90116100
Si	2.32840100	0.22300600	0.39356400
C	2.96155200	1.96103500	0.90274100
C	2.21013700	-0.78850400	2.07611400
C	3.70642600	-0.47553600	-0.82462100
H	3.23727300	2.54492200	0.00885600
H	3.84928800	1.90866100	1.55486700
H	2.17775800	2.52021000	1.43636800
C	1.40110700	0.08968000	3.05478500
C	1.45984400	-2.11954900	1.87786900
C	3.58380200	-1.07792800	2.70842800
C	3.45414700	0.12720900	-2.22214200
C	3.65455600	-2.00890300	-0.93751300
C	5.12357200	-0.06412600	-0.37510100
H	1.95755200	0.99213800	3.35195400
H	1.16821900	-0.47077700	3.98031000
H	0.44567100	0.42601500	2.62118000
H	0.45596600	-1.95951000	1.45444900
H	1.33728200	-2.64488400	2.84467600
H	1.99658400	-2.80450600	1.20308900
H	4.18232700	-1.77422800	2.10097600
H	3.46117500	-1.54298700	3.70584600
H	4.17763100	-0.16028300	2.84987800

H	2.49316800	-0.20290300	-2.65090200
H	4.25321100	-0.17542300	-2.92577400
H	3.43317500	1.22942800	-2.20245300
H	3.93562300	-2.50505900	0.00404900
H	4.35695900	-2.36586700	-1.71466900
H	2.65317900	-2.37048600	-1.21351100
H	5.25032800	1.02939500	-0.36071100
H	5.87940500	-0.46967700	-1.07482100
H	5.37266200	-0.44117500	0.62811600
C	-1.22712500	-0.81886300	-0.20840400
N	-2.35144000	-0.88346500	0.56934700
N	-1.28938200	-1.93396800	-1.00158000
C	-2.58263300	0.03777400	1.70065700
C	-3.11566500	-2.01182100	0.25021900
C	-0.26057600	-2.20235800	-2.02422600
C	-2.44808500	-2.66930900	-0.74868900
H	-1.84242500	0.82975900	1.55755900
C	-2.28159800	-0.63874100	3.04229800
C	-3.96313800	0.70319700	1.66759000
C	-4.42151700	-2.37271200	0.88154000
H	0.54923300	-1.49552700	-1.77967100
C	-0.75988900	-1.86341700	-3.43074100
C	0.34057500	-3.60448500	-1.90937300
C	-2.83519800	-3.93677700	-1.44112800
H	-3.02346500	-1.41261200	3.29119100
H	-2.29785600	0.11248500	3.84707900
H	-1.28597400	-1.10239500	3.03070900
H	-4.22636100	1.02263600	0.64826400
H	-3.94615400	1.59768500	2.30830600
H	-4.76122400	0.04653700	2.04102900
H	-4.36489600	-2.38597200	1.97973400
H	-4.72984100	-3.37608900	0.55975900
H	-5.22423100	-1.67190800	0.60089600
H	-1.55830800	-2.54694900	-3.75770100
H	0.07001900	-1.94520300	-4.14934700
H	-1.13804200	-0.83044600	-3.46150200
H	0.63006400	-3.82200600	-0.87075400
H	1.24369100	-3.66148300	-2.53463700
H	-0.34698800	-4.38645900	-2.25862400
H	-2.73419400	-3.86007000	-2.53314100
H	-3.88513900	-4.17597000	-1.22686200
H	-2.22608700	-4.79356900	-1.11037300

TS2b2’

B3LYP(D3)/def2-SVP

E = -3517.78378 a.u.

Al	2.73727300	0.77547200	-0.00172200
C	1.72552500	2.84822500	0.92317500
C	0.40203800	2.77696000	1.39553000
C	2.24879300	4.05577000	0.42036200
C	-0.44266100	3.86215500	1.17483900
H	0.01864500	1.85202200	1.82199400
C	1.37692700	5.12061100	0.20511300
H	3.30220100	4.12377800	0.14923200
C	0.02418400	5.03529800	0.56743600
H	-1.48980800	3.78021400	1.47390300
H	1.76514500	6.03705400	-0.24841000
F	2.72881600	2.00743100	1.69115500
H	-0.65334500	5.87141000	0.38522100
Si	5.25734600	0.84395100	-0.24924800

C	5.74090800	2.56306800	0.45643100	C	2.39325700	-1.07946100	0.90758900
C	6.30277300	-0.44403300	0.81440000	N	2.15670200	-1.51523600	2.17906500
C	5.80739400	0.91322100	-2.13741800	N	2.26243200	-2.19549800	0.12464300
H	5.45401700	3.35892200	-0.25028600	C	2.02136600	-0.57847400	3.31731600
H	6.82571200	2.65406400	0.63294400	C	1.90206400	-2.89012100	2.19634200
H	5.21754800	2.76328300	1.40404400	C	2.24895900	-2.09270600	-1.34814000
C	5.98583500	-0.12295000	2.28957200	C	1.96990300	-3.31889500	0.89789500
C	5.88106500	-1.90003400	0.54127300	H	2.39691300	0.37047200	2.92550400
C	7.82178700	-0.32374800	0.59768400	C	2.89424600	-0.94785200	4.51965200
C	4.76370800	1.75429800	-2.90269700	C	0.54481700	-0.37104700	3.67561300
C	5.85333200	-0.49052000	-2.76432600	C	1.63172500	-3.69664800	3.42359100
C	7.18389100	1.58462900	-2.31740100	H	2.68436400	-1.09951800	-1.55347000
H	6.37636400	0.86083200	2.59339800	C	0.80814700	-2.06495000	-1.86705800
H	6.43776000	-0.87815800	2.96128700	C	3.13651300	-3.11939600	-2.05148400
H	4.89986500	-0.11949600	2.47596000	C	1.78486200	-4.70089800	0.36409500
H	4.81302600	-2.06036300	0.74724400	H	2.48370100	-1.78271800	5.10444900
H	6.45009400	-2.59478700	1.18894000	H	2.95502400	-0.07850700	5.19216700
H	6.07220700	-2.20086300	-0.50048600	H	3.91663500	-1.20415800	4.20628000
H	8.11957300	-0.64944200	-0.41135800	H	-0.05001300	-0.16185100	2.77538800
H	8.36860700	-0.96341500	1.31762100	H	0.44703000	0.48153400	4.36484900
H	8.18472800	0.70709700	0.73818800	H	0.11334100	-1.25253400	4.17553900
H	3.76409600	1.28945100	-2.88986400	H	2.53149300	-3.81650500	4.04808800
H	5.06589200	1.87551300	-3.96068800	H	1.28738300	-4.70161200	3.14495400
H	4.65130000	2.76556100	-2.47731100	H	0.85148200	-3.24241700	4.05101300
H	6.65119100	-1.11310100	-2.32969600	H	0.28773000	-3.01680800	-1.68176300
H	6.04638200	-0.42793900	-3.85240800	H	0.80022100	-1.88074700	-2.95185700
H	4.90165900	-1.02962000	-2.63327200	H	0.24372500	-1.25799300	-1.37680600
H	7.19149100	2.61637900	-1.93237800	H	4.12076700	-3.18543000	-1.56722200
H	7.44785500	1.63673800	-3.39140600	H	3.29163600	-2.80239100	-3.09364200
H	7.99076500	1.03548500	-1.81075300	H	2.68523000	-4.12068400	-2.08001700

H	1.05863600	-4.72939200	-0.46273100
H	1.40922400	-5.36166900	1.15659700
H	2.72730800	-5.13350900	-0.00749300
Al	-2.54175800	0.57750100	-0.18101400
Si	-4.52215900	1.30777600	1.12864600
F	-1.25511500	0.06097900	0.87062800
N	-2.79434800	-1.46446500	-2.57820100
C	-1.27774900	-2.93991200	1.30751900
H	-0.88988000	-3.90982700	0.96436600
H	-1.28370700	-2.95037200	2.40766200
H	-0.60432700	-2.13918700	0.98459500
C	-6.28544000	-0.97658900	0.88876500
H	-5.39889000	-1.53955700	0.55830700
H	-7.17022200	-1.45804200	0.42964800
H	-6.37677300	-1.10213800	1.97915900
C	-2.73207300	2.88959500	-2.09772600
H	-3.81460900	2.85360200	-1.93070600
C	-2.68852600	-2.67685200	0.79410300
H	-3.00521200	-1.70573200	1.18588700
C	-1.89014400	1.96165500	-1.44754100
C	0.01116700	3.06429900	-2.54654900
H	1.09357400	3.13064700	-2.68930500
C	-0.85028800	3.95958900	-3.18811000
H	-0.45110400	4.73218800	-3.85179000
N	-2.70359900	-2.48201800	-0.67426600
C	-6.19022100	0.50185200	0.47934700
C	-7.44392100	1.25024700	0.97003000
H	-7.54908300	1.21723400	2.06417200
H	-8.35768700	0.79738800	0.53938100
H	-7.43349600	2.30978800	0.66923500
C	-2.77638000	-1.24637400	-1.23701200
C	-0.50778900	2.08382300	-1.69560600
H	0.20979800	1.41964600	-1.19918800
C	-3.70653300	-3.71390600	1.27597600
H	-3.34766600	-4.74601100	1.16105500
H	-4.66885100	-3.61411200	0.75377700
H	-3.88875800	-3.54836600	2.34841000
C	-4.20029100	1.06723700	3.05204700
C	-2.67929200	-3.42537300	-4.23016000
H	-3.68796800	-3.46691000	-4.67181100
H	-2.29948800	-4.45511900	-4.18436000
H	-2.03645300	-2.86471400	-4.92176300
C	-4.76935100	3.19246400	0.89051800
H	-5.41717600	3.62113200	1.67341600
H	-5.23797500	3.40774800	-0.08261700
H	-3.80945600	3.73068000	0.90690700
C	-2.62659200	-3.47033500	-1.65184900
C	-2.49799100	-4.93591800	-1.39008500
H	-1.74520600	-5.14791100	-0.61726100
H	-2.18689800	-5.45341800	-2.30764700
H	-3.44744200	-5.38817800	-1.06254000
C	-2.69031400	-2.82448300	-2.86192900
C	-5.45153700	1.34969100	3.90192100
H	-5.89058600	2.33690800	3.68460500
H	-5.19904800	1.33517000	4.97946400
H	-6.23556600	0.59212000	3.74436600
C	-1.58732500	-0.10322600	-4.28785800
H	-0.77486100	0.04204300	-3.56341000
H	-1.67279900	0.82622200	-4.87016700
H	-1.31827800	-0.91724000	-4.97846200

C	-2.22856500	3.87043200	-2.96112200
H	-2.91026800	4.57507200	-3.44741000
C	-2.90633500	-0.35850900	-3.56024500
H	-3.09335700	0.52250900	-2.93952400
C	-3.09171600	2.06831300	3.44329900
H	-2.17734200	1.91559700	2.84793900
H	-2.81338600	1.93702500	4.50603400
H	-3.40762700	3.11536000	3.31708600
C	-4.11091600	-0.51943300	-4.48887500
H	-3.96202500	-1.28910700	-5.25931900
H	-4.28226800	0.43575600	-5.00793300
H	-5.02032500	-0.76099300	-3.91760900
C	-6.15683700	0.57462300	-1.06286000
H	-6.05199900	1.60877300	-1.43054300
H	-7.09303300	0.17012000	-1.49280300
H	-5.32448000	-0.01376300	-1.48322700
C	-3.67895000	-0.34580000	3.37919900
H	-4.38879100	-1.13596000	3.08736600
H	-3.50770100	-0.44994300	4.46758900
H	-2.71672500	-0.53594900	2.87927200

INT-deN-a$_0$

B3LYP(D3)/def2-SVP

E = -3186.543572 a.u.

Si	1.52508800	3.12534600	-0.71910400
Al	0.44023700	1.16853500	0.36307800
Al	-1.95488900	0.16937000	0.23756900
Si	-2.69700000	-1.07521400	2.27346900
N	-3.64851600	-1.21772300	-1.96051900
C	0.78641800	3.59509400	-2.41878800
H	0.73690900	2.72853200	-3.09778100
H	1.38166300	4.37851300	-2.91758400
H	-0.23796500	3.97792500	-2.29356000
C	1.07604200	4.59864800	0.49765200
C	-0.41464700	4.45715100	0.87776000
H	-1.08329500	4.44340000	0.00322900
H	-0.72929500	5.30163000	1.51897200
H	-0.61611200	3.53877600	1.45663900
C	0.56795800	-0.69092300	-3.00999000
H	0.53147800	-1.03772900	-4.05434500
H	1.61967000	-0.55601300	-2.72490800
H	0.07870300	0.29326200	-2.95136100
C	-0.75647400	-3.11999900	1.63786200
H	-0.58719300	-2.62480700	0.66973000
H	-0.48825700	-4.18640400	1.51029100
H	-0.04048200	-2.68622200	2.34776700
C	-3.89635000	2.51213300	0.59046300
H	-4.18608800	2.05916800	1.54378100
C	-0.09321400	-1.67348700	-2.04432400
H	0.02666900	-1.25544100	-1.03543400
C	-2.93624600	1.87294400	-0.21977900
C	1.91345000	4.52151100	1.78821000
H	1.83489100	3.53168100	2.26610300
H	1.56199000	5.27577200	2.51743400
H	2.97957200	4.72626900	1.60264200
C	3.62629800	1.95976600	-2.21099600
H	3.15852400	0.97467800	-2.05065600
H	4.70278300	1.78453400	-2.39437400
H	3.20762500	2.39670500	-3.13154400
C	4.09911100	2.17372200	0.22449800

H	4.00378300	2.75576800	1.15260800
H	5.17948200	2.01879500	0.03937900
H	3.65676900	1.18316100	0.40740400
C	1.27061700	5.97471500	-0.16793600
H	2.31444800	6.15633800	-0.46434900
H	0.98430600	6.78162900	0.53340100
H	0.64265200	6.08899900	-1.06618700
C	-3.21654700	3.72161400	-1.82618400
H	-2.93762600	4.19056100	-2.77471300
C	4.17130300	4.20616400	-1.25641900
H	3.72683300	4.75917700	-2.10054900
H	5.23304800	4.02189700	-1.50962200
H	4.15976500	4.86873900	-0.37778200
C	-4.16522700	4.32851000	-0.99553300
H	-4.63369400	5.27201100	-1.28884700
N	-1.55889400	-1.69392200	-2.24467200
C	-2.21626100	-2.97522300	2.10739200
C	-2.40443000	-3.75084900	3.42423500
H	-1.71747200	-3.40585500	4.21105700
H	-2.20624100	-4.82909900	3.27033600
H	-3.43190300	-3.66140500	3.81366900
C	-2.40833200	-1.00932400	-1.43690600
C	-2.61795400	2.51911200	-1.43528000
H	-1.87102100	2.07492100	-2.10394400
C	3.44726800	2.87481600	-0.98419000
C	0.54410100	-3.06216300	-2.01657800
H	0.77820900	-3.45172000	-3.01789500
H	-0.10525500	-3.78188700	-1.49636100
H	1.47925000	-2.97618100	-1.44421800
C	-1.97476900	-0.25771200	3.90981900
C	-4.73924400	-2.46684400	-3.92375400
H	-5.29301200	-3.28669400	-3.43851800
H	-4.38893300	-2.83827200	-4.89625900
H	-5.45110400	-1.65323000	-4.11741900
C	-4.60053800	-1.03692500	2.51293200
H	-4.94000600	-0.01196600	2.73562600
H	-4.91996400	-1.68120400	3.34921300
H	-5.13703900	-1.37203900	1.61414500
C	-2.24756800	-2.31344800	-3.28391100
C	-1.61817500	-3.15609900	-4.34489200
H	-0.73593200	-2.67130800	-4.78660800
H	-2.33678900	-3.34018300	-5.15471000
H	-1.29738400	-4.13553400	-3.95617600
C	-3.57490800	-2.01416400	-3.10299600
C	-2.79981800	-0.64375100	5.15432300
H	-3.84463300	-0.30285200	5.08178200
H	-2.36741400	-0.17132600	6.05671100
H	-2.81517300	-1.72971300	5.33192800
C	-5.46145600	0.48694700	-2.19246700
H	-4.69651000	1.24391900	-2.40856400
H	-6.26348500	0.97909900	-1.62202300
H	-5.89302200	0.12804500	-3.13922400
C	-4.50321400	3.71826400	0.21663700
H	-5.24202500	4.18541000	0.87487400
C	-4.87822100	-0.65110600	-1.35406800
H	-4.52612300	-0.21372800	-0.41149700
C	-2.04815000	1.27509900	3.74409200
H	-1.39569100	1.63338400	2.93240600
H	-1.71265200	1.77958300	4.66953400
H	-3.07203700	1.62628200	3.53576100

C	-5.90792900	-1.72870000	-1.00497300
H	-6.44133800	-2.10668300	-1.88804800
H	-6.66043600	-1.29351700	-0.33072100
H	-5.43662600	-2.57511800	-0.48631900
C	-3.12737700	-3.61737400	1.04106200
H	-4.18604500	-3.61575500	1.34674100
H	-2.83895100	-4.67274000	0.87399900
H	-3.05427900	-3.10283400	0.06991100
C	-0.50244300	-0.64242400	4.14400600
H	-0.38137800	-1.71654400	4.35296700
H	-0.10215400	-0.09720100	5.01991200
H	0.13040700	-0.37466500	3.28724500
F	1.19060100	1.10017100	1.90790600
N	3.79615200	-1.51589700	1.16198300
N	4.37237100	-1.79253900	-0.88528000
C	3.27915500	-1.55505600	-0.09824700
C	2.91033000	-1.41530600	2.33974400
H	1.94817500	-1.12408000	1.90544800
C	5.17965900	-1.72687900	1.17261400
C	4.21152800	-2.07898600	-2.31938800
H	3.19191100	-1.73377100	-2.52786400
C	5.54859100	-1.90727900	-0.13418100
C	3.31283100	-0.31657700	3.32517200
H	4.17559800	-0.59814400	3.94557000
H	2.46496800	-0.11975000	3.99575000
H	3.52794500	0.62077100	2.79629000
C	4.25747000	-3.58695300	-2.59110800
H	5.25643100	-4.00484200	-2.39100500
H	4.01392500	-3.79120700	-3.64612100
H	3.53201100	-4.11744600	-1.95709600
C	2.73462800	-2.78115500	3.00978000
H	2.38156500	-3.52658200	2.28175000
H	1.98862600	-2.70515500	3.81557400
H	3.67287400	-3.14792900	3.45506900
C	5.16084600	-1.29482500	-3.23083700
H	5.22425700	-0.24359200	-2.92127300
H	4.77731900	-1.32329700	-4.26282400
H	6.17493700	-1.71751100	-3.25011300
C	6.91723600	-2.13004500	-0.69259400
H	6.95526100	-2.99111500	-1.37753600
H	7.62867900	-2.32792600	0.12080300
H	7.28816800	-1.25214000	-1.24677700
C	6.05413100	-1.69685200	2.38466800
H	6.16003100	-0.67765300	2.78988600
H	7.06080200	-2.05913000	2.13421800
H	5.66628200	-2.33246400	3.19426700

TS-deN-a$_0$

B3LYP(D3)/def2-SVP

E = -3186.516326 a.u.

Si	1.88954200	2.93533500	-0.91742700
Al	0.12538800	1.77209000	0.39458900
Al	-1.61033500	0.06790400	0.22745200
Si	-3.32981900	-0.51002400	1.97708400
N	-3.31686500	-0.98173200	-2.22247300
C	1.11698900	3.81608000	-2.43199700
H	0.83298900	3.08940800	-3.21067100
H	1.82882500	4.52586000	-2.88507100
H	0.20897500	4.37059200	-2.15372000
C	2.61455000	4.35951700	0.21539100

C	1.46306400	5.35478600	0.47825700
H	1.11055900	5.83953000	-0.44623000
H	1.80223200	6.15789600	1.15952000
H	0.60068200	4.86348900	0.95789800
C	0.93518800	-2.52042600	-2.54603800
H	0.87188300	-3.51235800	-3.01546500
H	1.93027400	-2.41882500	-2.09318700
H	0.82470700	-1.74638600	-3.31960400
C	-2.86880100	-3.31330200	2.70331500
H	-1.84289100	-3.19638000	2.31860800
H	-3.14488900	-4.37767100	2.58084000
H	-2.84074100	-3.11045100	3.78320600
C	-2.63942000	3.21808100	0.50131700
H	-2.56322900	3.10568500	1.58388400
C	-0.07505500	-2.32681800	-1.41797600
H	0.21020300	-1.40233900	-0.88961900
C	-1.80784100	2.43488600	-0.33300900
C	3.09318300	3.81882900	1.57830500
H	2.27461800	3.33049500	2.12718400
H	3.46494000	4.65134400	2.20584000
H	3.91989100	3.09930300	1.47687900
C	2.52303700	0.49349400	-2.19445000
H	2.11848100	-0.10599800	-1.36563900
H	3.21578400	-0.16070800	-2.75615400
H	1.69033900	0.74319500	-2.87516800
C	4.29632000	1.30761100	-0.63716700
H	4.91857500	2.14794900	-0.29291200
H	4.97849200	0.55053400	-1.06587400
H	3.82993600	0.84351700	0.24320500
C	3.77401700	5.10797100	-0.46756100

H	4.65930500	4.46602600	-0.59951900
H	4.08984700	5.97148900	0.14858500
H	3.49395200	5.50174200	-1.45845200
C	-2.81241800	3.59525500	-2.25600300
H	-2.86079000	3.75489600	-3.33668800
C	3.98500400	2.41101500	-2.86400100
H	3.29892000	2.66008800	-3.68865400
H	4.74768500	1.72073300	-3.27354400
H	4.50545500	3.33616200	-2.57324800
C	-3.62461100	4.34744500	-1.40084200
H	-4.31392200	5.09089300	-1.81101200
N	-1.44467900	-2.03128400	-1.92011800
C	-3.87699700	-2.41363300	1.96323000
C	-5.26614600	-2.57963800	2.61572900
H	-5.29535800	-2.21939200	3.65367200
H	-5.55303800	-3.64841800	2.63151100
H	-6.04894700	-2.04281500	2.05691300
C	-2.15446500	-0.94435800	-1.51206300
C	-1.93685700	2.64429400	-1.72346300
H	-1.30999500	2.06484300	-2.41108700
C	3.25378800	1.74828800	-1.67920200
C	-0.04958600	-3.45413400	-0.38965000
H	-0.34463900	-4.42546500	-0.81631000
H	-0.71731700	-3.23168800	0.45525800
H	0.98059300	-3.52353000	-0.00666000
C	-2.65409900	0.01346200	3.74716300
C	-4.50203200	-2.50917600	-3.91087700
H	-5.37004700	-2.83144700	-3.31393400
H	-4.20929400	-3.35009000	-4.55266900
H	-4.83539700	-1.69570600	-4.57009300

C	-4.93882400	0.49420900	1.71334100
H	-4.70915700	1.54531800	1.48260000
H	-5.58141400	0.47101400	2.60923400
H	-5.52783000	0.09430800	0.87512300
C	-2.17108500	-2.77634700	-2.84335200
C	-1.71450600	-4.05461200	-3.46923800
H	-0.97683300	-3.88417800	-4.26920600
H	-2.56966900	-4.58409300	-3.90972700
H	-1.25438300	-4.72595500	-2.73174000
C	-3.35393800	-2.10725300	-3.04231200
C	-3.58593200	-0.45621000	4.88152200
H	-4.61468200	-0.07938300	4.75861000
H	-3.21738000	-0.07690800	5.85299800
H	-3.63625200	-1.55199300	4.96417500
C	-4.24417900	0.91675300	-3.51603800
H	-3.21129900	1.18721900	-3.76887300
H	-4.82056300	1.84632800	-3.40835700
H	-4.67232400	0.34616600	-4.35355900
C	-3.54146400	4.15191100	-0.01741900
H	-4.17002500	4.74006000	0.65746300
C	-4.30021200	0.12734900	-2.20462200
H	-3.94666100	0.78186900	-1.39595400
C	-2.56640400	1.55286800	3.81078800
H	-1.80976600	1.95033800	3.11952600
H	-2.25423600	1.86478300	4.82502000
H	-3.53493200	2.03827000	3.60904300
C	-5.72821600	-0.31061000	-1.86929900
H	-6.22717700	-0.80344100	-2.71495400
H	-6.31693400	0.58259700	-1.61341800
H	-5.75465000	-0.98708300	-1.00602900
C	-3.98228600	-2.93092000	0.51534400
H	-4.64400600	-2.31016900	-0.10478500
H	-4.39491200	-3.95749000	0.50484000
H	-3.00507900	-2.97153000	0.01485000
C	-1.23667400	-0.54074200	3.98657600
H	-1.21362100	-1.63803800	4.04640600
H	-0.82667400	-0.15281600	4.93785000
H	-0.54898800	-0.23049900	3.18600100
F	0.12578400	2.35939900	2.02289600
N	3.50577200	-1.54064100	1.46376600
N	4.58610900	-2.39105000	-0.17901200
C	3.31742600	-2.17796600	0.27493300
C	2.34690900	-1.17869500	2.30579400
H	1.50415000	-1.20085900	1.59878500
C	4.86262100	-1.37510600	1.76601100
C	4.81010900	-3.13889900	-1.42469200
H	3.79719200	-3.23843300	-1.83737100
C	5.55481800	-1.91169700	0.71214900
C	2.42358500	0.23747600	2.88282500
H	3.04031700	0.29354400	3.79054900
H	1.41840000	0.59356500	3.14110400
H	2.82706100	0.94611800	2.14794800
C	5.33446800	-4.55249600	-1.15079900
H	6.35161400	-4.54189100	-0.72997200
H	5.36717000	-5.13310100	-2.08656100
H	4.67432700	-5.07293500	-0.44049600
C	2.10401500	-2.24181300	3.37971900
H	1.97948200	-3.23381500	2.91912300
H	1.19453900	-2.00233900	3.95018300
H	2.94541300	-2.29504000	4.08972600

C	5.65363300	-2.37572200	-2.45044900
H	5.27792200	-1.34975900	-2.57734000
H	5.59611800	-2.88513200	-3.42524400
H	6.71537900	-2.32437100	-2.17020700
C	7.03238400	-1.93222600	0.48610800
H	7.39488700	-2.91639000	0.15375000
H	7.56098300	-1.68551500	1.41734500
H	7.34449000	-1.19294200	-0.27089100
C	5.40834700	-0.70464500	2.98526800
H	5.25574100	0.38632400	2.96332400
H	6.48949000	-0.88552100	3.06333900
H	4.94245800	-1.08295100	3.90764700

P-deN-a$_0$

B3LYP(D3)/def2-SVP

E = -3186.523712 a.u.

Si	1.13703200	3.38817300	-0.69886300
Al	-0.55441400	2.23966700	0.72436600
Al	-1.38899600	-0.19857700	0.28863900
Si	-3.06595800	-1.49405300	1.65517200
N	-2.60073600	-0.87315600	-2.48907000
C	0.30304100	4.76235600	-1.74566000
H	-0.24597200	4.34074500	-2.60322900
H	1.04183000	5.47794600	-2.14511900
H	-0.42477000	5.32637900	-1.14211200
C	2.39034000	4.32442700	0.48663800
C	1.58216400	5.43668800	1.19010900
H	1.23383900	6.20734400	0.48419400
H	2.21083200	5.94457400	1.94583800
H	0.70105400	5.03500100	1.71789200
C	1.72544200	-2.20744300	-2.45414600
H	1.81918500	-3.12716400	-3.04827200
H	2.63401500	-2.10197400	-1.84821800
H	1.64767500	-1.34410500	-3.12832100
C	-2.67669000	-4.39726700	1.74610100
H	-1.61557500	-4.22588600	1.50629500
H	-2.94338400	-5.39544300	1.35082500
H	-2.76330100	-4.45121000	2.84112000
C	-3.25432800	3.11435900	1.66240600
H	-2.86952000	2.83345700	2.64731100
C	0.54850000	-2.26299200	-1.48192600
H	0.70310000	-1.43539700	-0.76596500
C	-2.40916100	3.00046800	0.53545800
C	2.93618700	3.37527300	1.57062700
H	2.12571900	2.96654000	2.19122400
H	3.63277100	3.91642700	2.23996400
H	3.49295100	2.53064700	1.13574700
C	0.88403500	1.52032600	-2.81616000
H	0.24579300	0.89504700	-2.17358700
H	1.31271900	0.86576800	-3.59910800
H	0.23128200	2.24411800	-3.33141000
C	2.86128300	1.13899800	-1.32922500
H	3.72328400	1.56904100	-0.79618600
H	3.26525600	0.42510900	-2.07082700
H	2.28366900	0.55118400	-0.59697700
C	3.57363700	4.96830600	-0.25673300
H	4.26765900	4.21090000	-0.65403600
H	4.15703700	5.60908000	0.43199500
H	3.25071500	5.60339300	-1.09808200
C	-4.27476500	3.86273400	-0.82047400

H	-4.66243400	4.17625400	-1.79546600
C	2.87951800	2.99250900	-3.02248300
H	2.30856500	3.77721300	-3.54449100
H	3.27558300	2.30669900	-3.79682400
H	3.74359000	3.47371200	-2.54410200
C	-5.09088500	3.94576400	0.31312100
H	-6.11945300	4.30769800	0.22838600
N	-0.74483800	-1.93094600	-2.13263700
C	-3.59874600	-3.32174800	1.14218400
C	-5.04546000	-3.60296000	1.60284000
H	-5.17287400	-3.49148900	2.68974300
H	-5.33028800	-4.64040600	1.34405500
H	-5.77192800	-2.93609100	1.11311000
C	-1.57865100	-1.00229900	-1.60842400
C	-2.95837800	3.39952800	-0.70164200
H	-2.33537600	3.36626700	-1.60075000
C	2.00578500	2.21706200	-2.01772000
C	0.50076800	-3.56348600	-0.68547000
H	0.34454600	-4.43709900	-1.33753400
H	-0.30658800	-3.54112800	0.05984200
H	1.46490900	-3.66806600	-0.16256100
C	-2.53885600	-1.36338200	3.53369500
C	-3.40721300	-1.93402000	-4.67767500
H	-4.35724400	-2.36862600	-4.32807600
H	-2.98892000	-2.61005600	-5.43460000
H	-3.64148900	-0.98413700	-5.17981700
C	-4.64464300	-0.42693600	1.44304100
H	-4.42551400	0.64867000	1.51845000
H	-5.39620800	-0.67470800	2.21124700
H	-5.11024600	-0.60070600	0.46029100
C	-1.26394500	-2.43546700	-3.32513800
C	-0.63303700	-3.51511800	-4.14262200
H	0.22850300	-3.14784100	-4.72196700
H	-1.36383300	-3.92487200	-4.85245200
H	-0.28142900	-4.34505200	-3.51336000
C	-2.43622900	-1.75788700	-3.55642400
C	-3.57095700	-2.01402700	4.47455300
H	-4.57814000	-1.58546700	4.34424000
H	-3.28255500	-1.84794500	5.52939600
H	-3.64648100	-3.10243400	4.32896100
C	-3.26775800	1.32849100	-3.38729900
H	-2.22353100	1.65723200	-3.28858200
H	-3.92065700	2.19323900	-3.20715400
H	-3.43004500	0.98461400	-4.41970600
C	-4.57108600	3.57701100	1.55931400
H	-5.19674400	3.65075700	2.45413200
C	-3.58499100	0.22883300	-2.37137700
H	-3.41142000	0.65003400	-1.36823300
C	-2.43163100	0.13477800	3.89140200
H	-1.67067900	0.66900200	3.30079200
H	-2.14209300	0.24645700	4.95291600
H	-3.39077800	0.65986900	3.76088600
C	-5.04252200	-0.22913500	-2.42831600
H	-5.36794600	-0.46544000	-3.45105100
H	-5.67893800	0.58680800	-2.05517400
H	-5.21372900	-1.10932800	-1.79358000
C	-3.55722200	-3.46151100	-0.39235900
H	-4.15021300	-2.68456500	-0.89817600
H	-3.97103500	-4.44024600	-0.70010000
H	-2.53313500	-3.40157900	-0.78700900

C	-1.16054200	-2.01327200	3.76381800
H	-1.17604000	-3.10511000	3.63683700
H	-0.80441200	-1.80875500	4.79078700
H	-0.40246500	-1.61034700	3.07457900
F	-0.14796100	2.29783500	2.40374400
N	3.65606100	-1.39001200	1.59896800
N	4.90081600	-2.01233200	-0.02878100
C	3.67162700	-2.22420200	0.52402000
C	2.47943400	-1.34492200	2.48723500
H	1.73013200	-1.92303400	1.92555000
C	4.85229800	-0.67403500	1.73352400
C	5.33378500	-2.80356300	-1.18983900
H	4.42654400	-3.36481500	-1.45495800
C	5.64723200	-1.06734100	0.68809100
C	1.94023300	0.06999200	2.67920300
H	2.55973900	0.67594700	3.35322700
H	0.93045700	0.05210700	3.10720200
H	1.87403200	0.59600200	1.71931400
C	6.40867400	-3.82962900	-0.81579700
H	7.36067700	-3.35176800	-0.53917600
H	6.60735800	-4.49654900	-1.66981900
H	6.07140500	-4.44298500	0.03325200
C	2.73430600	-2.08089500	3.80513300
H	3.08788100	-3.10552700	3.61371200
H	1.80154400	-2.13483500	4.38729500
H	3.48474200	-1.56500100	4.42395600
C	5.72203400	-1.93981500	-2.39382900
H	4.95186000	-1.17967600	-2.59352900
H	5.82132700	-2.57437100	-3.28850200
H	6.68259200	-1.42513200	-2.24833400

C	7.00417500	-0.56342400	0.31387100
H	7.70674400	-1.37642200	0.07805500
H	7.43540100	0.01295800	1.14399900
H	6.96852500	0.10592300	-0.56228300
C	5.15555500	0.32699900	2.80239500
H	4.61674400	1.27502100	2.65050000
H	6.23013400	0.55699600	2.81018300
H	4.89112300	-0.04538300	3.80285900

INT-deN-b$_0$

B3LYP(D3)/def2-SVP

E = -3186.534207 a.u.

Si	-2.33284700	-2.91788400	0.03830500
Al	-2.01141200	-0.53267200	-0.66304300
Al	0.30091200	0.66191300	-0.84272300
Si	1.18830300	2.95556100	-0.28457800
N	-3.29837400	0.95225500	1.71710400
F	-2.61953000	-0.49581900	-2.29701800
N	-4.25702700	1.57661700	-0.11911700
C	-3.32580700	0.72043000	0.37676900
C	-1.82097200	-3.36506600	1.83453000
H	-0.83599800	-2.94933300	2.09617300
H	-1.76484500	-4.45780600	1.97192100
H	-2.55116800	-2.98329100	2.56490100
C	-4.24988200	-3.35902700	-0.06347000
C	-5.03184200	-2.32447100	0.77143900
H	-4.72318700	-2.32610900	1.82987800
H	-6.11430400	-2.55294000	0.75162000
H	-4.90549300	-1.30068400	0.39007900
C	-2.37818300	0.22829900	2.62079200

H	-1.97848800	-0.58319800	1.99566200
C	2.30663700	3.01969800	-2.96675600
H	2.12738000	1.93872500	-3.07305000
H	3.14832900	3.27476200	-3.63807700
H	1.41942900	3.55014600	-3.34616800
C	-4.74011900	-3.27213600	-1.52185700
H	-4.44755300	-2.32267600	-2.00005700
H	-5.84319800	-3.35356900	-1.56669600
H	-4.33277700	-4.08904600	-2.13726700
C	0.25131200	-3.80112900	-0.71197200
H	0.53803600	-2.74531600	-0.82591100
H	0.93333900	-4.38014000	-1.36132700
H	0.44989600	-4.10796100	0.32823600
C	-1.37096600	-3.65884800	-2.60385000
H	-2.38397600	-3.85780700	-2.98297600
H	-0.66357400	-4.24009600	-3.22450100
H	-1.16044800	-2.59505800	-2.77954400
C	-4.57337900	-4.75245700	0.50982200
H	-4.08981300	-5.56300000	-0.05203500
H	-5.66434600	-4.93726600	0.47514900
H	-4.26252300	-4.84609200	1.56331500
C	-4.19506700	1.96263000	2.06435600
C	-4.64223300	1.58760100	-1.55323400
H	-3.90634900	0.93246900	-2.03185200
C	-4.80688600	2.35319200	0.90052400
C	-3.09983000	-0.43412000	3.79654200
H	-3.38013200	0.28518600	4.57807100
H	-2.42700500	-1.17333900	4.25551800
H	-4.00314800	-0.96129000	3.45844800
C	-1.51807600	-5.54863300	-0.96742800
H	-1.47887300	-5.88570100	0.08167500
H	-0.77420000	-6.14467000	-1.52938800
H	-2.50834200	-5.81203800	-1.36925800
C	2.65016200	3.40680500	-1.51356900
C	3.02525400	4.90004000	-1.47261100
H	2.23646200	5.53928600	-1.89798800
H	3.93896900	5.07678000	-2.07109400
H	3.23394400	5.25324300	-0.44935100
C	-1.21700300	-4.04648100	-1.11977000
C	-0.27731200	4.27012900	-0.33273300
C	1.91631200	3.03616300	1.48063900
H	1.12817000	2.95333100	2.24638200
H	2.42817800	4.00050100	1.64212600
H	2.65410900	2.23664300	1.66396400
C	0.13620900	5.64162300	0.23734300
H	0.50471900	5.55951800	1.27264300
H	-0.73532200	6.32409500	0.25521100
H	0.91857400	6.13217100	-0.35639600
C	-4.50608800	2.97179100	-2.19067900
H	-5.31685100	3.65995400	-1.91259900
H	-4.53332200	2.85958600	-3.28521600
H	-3.54447600	3.43396100	-1.92506300
C	-1.20669800	1.10861400	3.05868100
H	-0.63001200	1.44938900	2.19048800
H	-0.53423800	0.53631700	3.71452000
H	-1.54235600	1.99373300	3.61778800
C	-1.43867300	3.74597000	0.53333300
H	-1.79819700	2.76400700	0.19651000
H	-2.29726900	4.44330000	0.49178600
H	-1.15338700	3.65406700	1.59262500

C	-6.01459100	0.94284000	-1.76183100
H	-6.03427700	-0.07269100	-1.33949400
H	-6.21718600	0.86245200	-2.84076800
H	-6.83224100	1.52534500	-1.30972500
C	-5.82744600	3.42836500	0.71154300
H	-5.38702900	4.33256200	0.26140500
H	-6.25824800	3.71398600	1.68017900
H	-6.65547900	3.10555400	0.06590300
C	3.88350400	2.59143400	-1.07589100
H	4.24405800	2.88004200	-0.07658700
H	4.71373400	2.75491100	-1.78789500
H	3.68113800	1.51111400	-1.05433500
C	-4.42291800	2.48351100	3.44668000
H	-4.99397900	1.77606200	4.06902200
H	-4.99186500	3.42133800	3.40333900
H	-3.47988300	2.69932400	3.96808000
C	-0.78928100	4.44856300	-1.77348800
H	-0.03511500	4.91113000	-2.42797700
H	-1.68045000	5.10486700	-1.79288500
H	-1.08007000	3.48673200	-2.23119900
C	1.51758200	-0.47450600	-1.94358100
C	1.03802200	-0.92102200	-3.19531500
C	2.78088100	-0.95790400	-1.54199300
H	0.04976700	-0.60064400	-3.54612600
C	1.77670200	-1.79118100	-4.00486600
C	3.51734900	-1.84667000	-2.33188900
H	3.20854800	-0.64984200	-0.58509300
C	3.01844600	-2.26477000	-3.57014700
H	1.37221800	-2.11652500	-4.96769200
H	4.48252900	-2.21451100	-1.97555300
H	3.59198400	-2.95900900	-4.19059600
N	3.77665600	-1.08961200	2.02994700
N	5.56094200	-0.24899400	1.20350800
C	4.47824000	0.07894300	1.96278300
C	2.47613600	-1.10761700	2.71271300
H	2.50387900	-0.19020900	3.31820800
C	4.39473100	-2.12807800	1.31825600
C	6.60798800	0.75598200	0.95613800
H	6.11444800	1.69275200	1.25135100
C	5.54867300	-1.59335800	0.80756300
C	2.28148800	-2.29401800	3.66130100
H	2.06050400	-3.22950500	3.12869500
H	1.42736900	-2.09020900	4.32601200
H	3.17382100	-2.44687300	4.28723000
C	7.00650800	0.87603600	-0.51867800
H	7.70413000	0.08599800	-0.83209900
H	7.51225700	1.84034400	-0.68124400
H	6.12214400	0.84264800	-1.17018300
C	1.33115700	-0.97447000	1.70761600
H	1.43927200	-0.00794100	1.18359800
H	0.36026700	-0.98467000	2.21707400
H	1.33362200	-1.78278900	0.96721400
C	7.81223000	0.54447300	1.87859500
H	7.48890300	0.52266200	2.93013800
H	8.53746400	1.36419800	1.75244200
H	8.33320200	-0.40074700	1.65702400
C	6.59790200	-2.26204400	-0.02114200
H	6.61820900	-1.88391100	-1.05565900
H	6.41011900	-3.34335500	-0.06776100
H	7.60694700	-2.12187500	0.39729900

C	3.84152700	-3.50401200	1.13118300
H	3.85369300	-4.09791300	2.05931000
H	4.43196400	-4.04783100	0.38165200
H	2.80558200	-3.47950600	0.76033000

TS-deN-b$_0$

B3LYP(D3)/def2-SVP

E = -3186.519663 a.u.

Si	-3.02092800	-2.50120400	0.06467300
Al	-1.67913600	-0.38530500	-0.06940200
Al	0.17336800	0.59957600	-1.52242000
Si	1.76780400	2.41078800	-0.93263400
N	-2.54634900	1.38462500	2.19059200
F	-1.39357200	1.10819700	-2.21514100
N	-3.45844700	2.17645900	0.39334100
C	-2.74486500	1.12595900	0.87182600
C	-2.91351000	-3.13468400	1.87221100
H	-1.88551100	-3.44174500	2.11925600
H	-3.56827200	-4.00940700	2.02311200
H	-3.21661100	-2.36632200	2.60018900
C	-4.92265000	-2.21315100	-0.30106200
C	-5.49305000	-1.36524200	0.85556400
H	-5.49553100	-1.91913200	1.80798400
H	-6.54031900	-1.07811500	0.64414500
H	-4.92003100	-0.43728000	1.01334600
C	-1.76500100	0.46133900	3.04119500
H	-1.62863700	-0.43374600	2.41087900
C	2.33814900	2.69852100	-3.75263000
H	1.78488900	1.76351900	-3.94025600
H	3.07084900	2.81261500	-4.57402300
H	1.62504400	3.53286100	-3.83472000
C	-5.10527400	-1.44460600	-1.62350300
H	-4.53609800	-0.50324800	-1.63312000
H	-6.16910600	-1.18705600	-1.78333500
H	-4.77516000	-2.03019000	-2.49324400
C	-0.67350000	-3.83194700	-0.81479400
H	-0.23361200	-2.87461900	-1.13122400
H	-0.17003300	-4.62039500	-1.40335100
H	-0.41027900	-3.99967200	0.24204400
C	-2.47721000	-3.63761800	-2.53528500
H	-3.53878700	-3.78696600	-2.78806100
H	-1.89029500	-4.33791600	-3.15707000
H	-2.18628700	-2.62156500	-2.84654900
C	-5.72144500	-3.52756900	-0.37668900
H	-5.43489400	-4.13348700	-1.24941500
H	-6.80312700	-3.31446000	-0.47115200
H	-5.58936700	-4.14954900	0.52381100
C	-3.10696900	2.61196300	2.54375500
C	-3.96573300	2.22272700	-1.00139100
H	-3.51345500	1.34388700	-1.47593600
C	-3.68508100	3.11312300	1.40426800
C	-2.54291500	-0.00165000	4.27373200
H	-2.59532500	0.77539500	5.04901000
H	-2.03424300	-0.87276900	4.71308200
H	-3.56601000	-0.30274300	4.00303000
C	-2.68508000	-5.29832600	-0.65675000
H	-2.46047900	-5.53940100	0.39493400
H	-2.17223400	-6.05546000	-1.27888900
H	-3.76592500	-5.43315100	-0.80687100
C	3.06014500	2.66902900	-2.38979800

C	3.89662900	3.95159100	-2.22995400
H	3.28786700	4.86310700	-2.33091600
H	4.67891500	3.99883900	-3.01143700
H	4.40950600	3.99453900	-1.25459400
C	-2.19854600	-3.88635700	-1.04144400
C	0.84239900	4.07610900	-0.44557800
C	2.76786400	1.91161600	0.61611200
H	2.13412000	1.89003700	1.51651200
H	3.59067100	2.61741400	0.81864300
H	3.20484500	0.90678300	0.51713600
C	1.75423200	5.07607800	0.29121500
H	2.17269900	4.64391600	1.21454000
H	1.17833400	5.97535700	0.58451400
H	2.59614800	5.41800700	-0.32653800
C	-3.45638600	3.43128800	-1.78423700
H	-3.91678100	4.37442300	-1.45772100
H	-3.70377200	3.28858300	-2.84650700
H	-2.36645700	3.51005900	-1.70978900
C	-0.37481200	1.01315300	3.35628900
H	0.18425600	1.19976200	2.42953300
H	0.19204800	0.28438400	3.95207900
H	-0.42521900	1.94991100	3.92910800
C	-0.31083200	3.70799500	0.50747600
H	-1.01623900	3.00557000	0.04501800
H	-0.88238400	4.61135600	0.79694300
H	0.05414000	3.24684000	1.43840200
C	-5.48898300	2.07409100	-1.04433700
H	-5.82385300	1.21936500	-0.44172900
H	-5.80577500	1.90088600	-2.08370200
H	-6.00505200	2.97747600	-0.68728900

C	-4.35871300	4.43306600	1.21696600
H	-3.71514800	5.13389000	0.66120200
H	-4.58027100	4.88535800	2.19238300
H	-5.30749100	4.34665400	0.67066700
C	4.02583000	1.46552500	-2.39071900
H	4.62473200	1.41353300	-1.46942400
H	4.73472400	1.54819600	-3.23625700
H	3.49514800	0.50989500	-2.50810600
C	-3.06733600	3.21267000	3.91112200
H	-3.73722300	2.69018000	4.61288500
H	-3.38532000	4.26237200	3.86890300
H	-2.05553000	3.19525500	4.34020500
C	0.24711900	4.74852500	-1.69609200
H	1.02821000	5.16364000	-2.35094800
H	-0.41706600	5.58758200	-1.41176500
H	-0.34724500	4.04332600	-2.29975800
C	0.85581000	-0.86424200	-2.69099400
C	0.13026600	-1.25642500	-3.83585200
C	2.00581900	-1.62248500	-2.38299800
H	-0.77468500	-0.70345300	-4.11109000
C	0.52566300	-2.34042000	-4.62913600
C	2.41177600	-2.70765800	-3.16626300
H	2.60508300	-1.36638400	-1.50238100
C	1.66920700	-3.07285900	-4.29485300
H	-0.06270600	-2.61890200	-5.50882200
H	3.30958400	-3.27292900	-2.89757600
H	1.98080900	-3.92201100	-4.90954500
N	3.24827900	-1.74192400	2.37732600
N	5.04494200	-0.75181800	1.77167500
C	4.13132000	-0.78464700	2.78426200

C	2.13808500	-2.12289100	3.25995600
H	2.11257700	-1.29345600	3.98229900
C	3.60722300	-2.31918400	1.15081600
C	6.25882900	0.06816700	1.92049100
H	6.02793700	0.67463100	2.80822500
C	4.75175600	-1.67536200	0.75621700
C	2.44874200	-3.40845100	4.03370500
H	2.52370700	-4.27824100	3.36216200
H	1.65216600	-3.61770200	4.76602400
H	3.40198000	-3.30461700	4.57312700
C	6.51423000	1.02361300	0.75275700
H	6.81149600	0.50167800	-0.16766300
H	7.33170800	1.71213300	1.01836600
H	5.62007300	1.62320300	0.53829800
C	0.78411300	-2.18307000	2.54722700
H	0.65294300	-1.32625600	1.86863800
H	-0.02567400	-2.17114100	3.29325700
H	0.66148100	-3.09968100	1.95632500
C	7.47632600	-0.80233800	2.25153900
H	7.26126600	-1.44365300	3.11962400
H	8.34365500	-0.16748500	2.49320800
H	7.76141200	-1.44929000	1.40706600
C	5.51621700	-1.84144100	-0.51815800
H	5.36346200	-0.98932500	-1.20067700
H	5.17587900	-2.74227800	-1.04738400
H	6.59897300	-1.94445900	-0.35097600
C	2.86918700	-3.42425600	0.46669300
H	2.68140200	-4.27191300	1.14422300
H	3.45550200	-3.80411400	-0.38026400
H	1.90091200	-3.10121100	0.05934100

P-deN-b$_0$

B3LYP(D3)/def2-SVP

E = -3186.522422 a.u.

Si	2.93905000	-2.50586200	-0.42076100
Al	1.78665200	-0.33025400	0.10889300
Al	0.13080000	0.65898900	1.90606500
Si	-1.72465200	2.08625200	1.07105000
N	2.61991800	1.52587400	-2.20703800
F	1.30047100	1.63069600	2.76010700
N	3.43293500	2.26466800	-0.34129500
C	2.76976000	1.21580000	-0.89302000
C	2.86565000	-3.01937500	-2.26224900
H	1.82979700	-3.23771700	-2.56661900
H	3.46429400	-3.92800900	-2.44308500
H	3.25154700	-2.23245200	-2.92657900
C	4.83173900	-2.25770300	0.02636800
C	5.44850600	-1.33112500	-1.04309000
H	5.47752700	-1.81148800	-2.03435300
H	6.49083800	-1.07432500	-0.77613900
H	4.89238300	-0.38553200	-1.14902300
C	1.91460900	0.62163700	-3.14041900
H	1.73899500	-0.28771500	-2.54320700
C	-2.54122100	2.30352700	3.83581300
H	-1.88980300	1.44831500	4.07911900
H	-3.34255200	2.33081700	4.59873200
H	-1.94418000	3.22091800	3.95427300
C	4.96604300	-1.58779000	1.40816600
H	4.40100800	-0.64329400	1.47297600
H	6.02260800	-1.34380300	1.62593700

H	4.60320500	-2.23255200	2.22067800
C	0.56404200	-3.89137800	0.25611200
H	0.11394600	-2.92514300	0.52899900
H	0.02131000	-4.66216500	0.83266200
H	0.36700300	-4.08345300	-0.81081600
C	2.24125600	-3.74285500	2.10059900
H	3.28701500	-3.87845900	2.41809800
H	1.63492600	-4.48159500	2.65467000
H	1.90212200	-2.75004000	2.43771600
C	5.62510100	-3.57713500	0.03858400
H	5.31090400	-4.23741400	0.86063500
H	6.70400900	-3.37569400	0.17879900
H	5.51723300	-4.13760100	-0.90451900
C	3.17619100	2.77506900	-2.48600700
C	3.84706100	2.27677200	1.08552600
H	3.28165600	1.44685400	1.53500400
C	3.69576800	3.23847100	-1.30248600
C	2.79453000	0.20539000	-4.32086900
H	2.90499100	1.01053600	-5.06060200
H	2.33136100	-0.65180600	-4.83161000
H	3.79409400	-0.09955800	-3.97721300
C	2.59223300	-5.33571600	0.18337400
H	2.46059400	-5.53639200	-0.89244700
H	2.03162300	-6.11701800	0.72963700
H	3.65611700	-5.47505400	0.42174900
C	-3.14725700	2.17378200	2.42370600
C	-4.13927700	3.32696000	2.19130900
H	-3.67216700	4.31208200	2.34398500
H	-4.98434300	3.25675200	2.90290400
H	-4.56766700	3.31152400	1.17536500
C	2.06982300	-3.94078900	0.58226600
C	-1.01347500	3.85728600	0.59166800
C	-2.53441000	1.41816700	-0.52404300
H	-1.83494500	1.45014100	-1.37380600
H	-3.42158300	1.99729300	-0.82626600
H	-2.85402400	0.37190000	-0.41115800
C	-2.02546700	4.74378000	-0.15791900
H	-2.39690800	4.25668100	-1.07447100
H	-1.54935900	5.69574500	-0.46443700
H	-2.89801100	5.00310300	0.45692500
C	3.40711400	3.53103400	1.84235900
H	4.07147800	4.38742800	1.65974100
H	3.42286000	3.30448600	2.91694100
H	2.37549500	3.80517300	1.59483700
C	0.55349100	1.17619600	-3.56316700
H	-0.07165300	1.39893600	-2.68857300
H	0.02328500	0.43431800	-4.17683600
H	0.65080200	2.09382600	-4.16006900
C	0.19049400	3.63529300	-0.34464500
H	0.96092000	3.00695200	0.12068300
H	0.66419200	4.60106300	-0.60881800
H	-0.10281000	3.15364000	-1.29084100
C	5.34652100	2.00021600	1.21822000
H	5.64471700	1.11032500	0.64871700
H	5.59507300	1.83014000	2.27654800
H	5.94768100	2.85263700	0.86681700
C	4.37103400	4.54369100	-1.03563200
H	3.71852500	5.22478300	-0.46715400
H	4.62838100	5.03629300	-1.98225700
H	5.30134500	4.41915500	-0.46419300

C	-3.92757200	0.84460400	2.35609800
H	-4.43791500	0.71799900	1.38990900
H	-4.70239000	0.81475400	3.14582600
H	-3.27392800	-0.02703700	2.51143700
C	3.19638700	3.43185300	-3.82798900
H	3.89806100	2.93954900	-4.52039700
H	3.51086500	4.47854800	-3.72714400
H	2.20542100	3.43411200	-4.30287800
C	-0.51805600	4.58366200	1.85659500
H	-1.35218500	4.87621300	2.51339600
H	0.02205300	5.51301900	1.58890900
H	0.16574100	3.95185700	2.44653000
C	-0.34649200	-0.91669300	3.03510700
C	0.32194500	-1.18131500	4.24913700
C	-1.30236500	-1.86939800	2.62452700
H	1.07219100	-0.47072800	4.61344000
C	0.05876000	-2.33119500	5.00415600
C	-1.57449000	-3.02418200	3.36509400
H	-1.85584200	-1.71034500	1.69218600
C	-0.88884300	-3.26052000	4.56194100
H	0.59655600	-2.50618600	5.94119100
H	-2.32023100	-3.74191900	3.00890900
H	-1.09379500	-4.16148700	5.14709900
N	-3.43526800	-1.51119300	-2.38879000
N	-5.26963400	-0.57561800	-1.81291800
C	-4.30330900	-0.53127400	-2.77417900
C	-2.24535900	-1.78554900	-3.20245300
H	-2.25276900	-0.95456800	-3.92342800
C	-3.84969900	-2.16787900	-1.22062000
C	-6.43530100	0.31656400	-1.91423800
H	-6.16020600	0.96957300	-2.75509700
C	-5.02426300	-1.56514900	-0.85027900
C	-2.37444300	-3.09338500	-3.99037700
H	-2.37347300	-3.97412000	-3.32972400
H	-1.53015900	-3.20156900	-4.69034500
H	-3.30957200	-3.09690700	-4.57014400
C	-6.63240500	1.19909000	-0.67880300
H	-6.96642400	0.62894200	0.19980200
H	-7.40014600	1.96010500	-0.88891800
H	-5.69961400	1.71807900	-0.41664700
C	-0.94445900	-1.71177600	-2.39878100
H	-0.90073000	-0.78271900	-1.81095400
H	-0.08465600	-1.74093800	-3.08514000
H	-0.83344900	-2.55575000	-1.70991600
C	-7.70310400	-0.44654900	-2.31254400
H	-7.51897500	-1.04685100	-3.21637700
H	-8.51921700	0.26195500	-2.52612300
H	-8.04985000	-1.12115100	-1.51448300
C	-5.87698100	-1.85361300	0.34403100
H	-5.78349800	-1.07532400	1.11812500
H	-5.57291300	-2.80365700	0.80503200
H	-6.94278800	-1.93858300	0.08538300
C	-3.12596500	-3.28877300	-0.54542600
H	-2.85569400	-4.09307700	-1.24653900
H	-3.75975100	-3.73228900	0.23477900
H	-2.19798600	-2.96077700	-0.05405800

NHC

B3LYP(D3)/def2-SVP

E = -540.331355 a.u.

N	1.06959600	-0.40531600	0.11585400
N	-1.06960400	-0.40529800	0.11592700
C	-0.00001300	-1.24368200	0.23273100
C	2.43141900	-0.95957800	0.14176500
H	2.25271500	-2.01179000	0.40638400
C	0.68540500	0.92826800	-0.08182400
C	-2.43141300	-0.95957800	0.14179300
H	-2.25271700	-2.01176300	0.40654700
C	-0.68541500	0.92828200	-0.08178800
C	3.31109700	-0.34253300	1.23333800
H	3.61633800	0.68743700	0.99662600
H	4.23026800	-0.93843700	1.34788700
H	2.78371700	-0.33632200	2.19953300
C	-3.08817900	-0.93246300	-1.24230500
H	-3.30455900	0.09299000	-1.58024500
H	-4.04237800	-1.48259800	-1.21886900
H	-2.43113700	-1.40919600	-1.98526100
C	3.08833800	-0.93233800	-1.24226000
H	2.43123000	-1.40869700	-1.98539500
H	4.04235000	-1.48280300	-1.21881800
H	3.30514000	0.09310600	-1.57995900
C	-3.31126100	-0.34246400	1.23319700
H	-2.78392000	-0.33587800	2.19941100
H	-4.23029200	-0.93855700	1.34785700
H	-3.61675400	0.68736900	0.99620600
C	-1.61457000	2.09335000	-0.20830200
H	-2.42588100	1.90457800	-0.92715000
H	-1.06700000	2.98009700	-0.55752900
H	-2.08534300	2.35839700	0.75354000
C	1.61458800	2.09331100	-0.20833600
H	2.08497300	2.35868400	0.75361100
H	1.06716200	2.97993800	-0.55810300
H	2.42617700	1.90429500	-0.92679500

INT-deN-a

B3LYP(D3)/def2-SVP

E = -2646.178797 a.u.

Si	3.77712000	-0.54292800	0.66422000
Al	1.40566200	-1.29349100	0.36085400
Al	-0.91755200	-0.16146400	0.66947000
Si	-2.95991700	-1.61326100	0.71722000
N	-1.09677400	2.49666800	-1.04475100
C	4.10848700	-0.28973600	2.53318700
H	3.60692700	0.61745100	2.90662800
H	5.18650900	-0.18021200	2.73832000
H	3.73762800	-1.13805200	3.12958000
C	4.97638100	-1.95864100	0.05590900
C	4.76997900	-3.16075000	1.00308200
H	5.06135400	-2.93052100	2.04047300
H	5.38891000	-4.01580800	0.67276800
H	3.72143400	-3.50206800	1.00446800
C	1.16120800	-0.85238500	-2.85909500
H	1.56327300	-0.37490500	-3.76510300
H	1.35912300	-1.93160500	-2.91475200
H	1.73013000	-0.44976000	-2.00645700
C	-4.29050700	-0.81409600	-1.72937500
H	-3.34335400	-0.33085600	-2.01759500
H	-5.10634300	-0.27221200	-2.24530000
H	-4.28144600	-1.84178800	-2.12514800
C	-1.94978100	1.42049700	3.06316600

H	-2.94942100	1.22238700	2.66674700	C	-5.83610100	-1.43021500	0.15483000
C	-0.33748400	-0.60160900	-2.68640200	H	-5.90640200	-2.47114100	-0.18880400
H	-0.67893800	-1.18824300	-1.82513100	H	-6.66896700	-0.87425200	-0.31722200
C	-0.81667300	0.93440100	2.37139200	H	-6.01686900	-1.42497200	1.24158900
C	4.61887900	-2.41638100	-1.37362600	C	-0.94002200	1.14464000	-1.01178300
H	3.58430500	-2.78927100	-1.42676400	C	0.43109400	1.22824300	2.96297000
H	5.28420600	-3.24384300	-1.68450200	H	1.34970800	0.86729200	2.49249900
H	4.73612300	-1.61311900	-2.11673000	C	4.11706500	1.20865900	-0.16638700
C	2.86512000	2.08652400	0.04060900	C	-1.15040600	-1.07632000	-3.89227100
H	1.97540600	1.67758800	-0.46810900	H	-0.76700600	-0.68434300	-4.84388500
H	3.03058900	3.10359100	-0.36271500	H	-2.20970200	-0.80267700	-3.79482700
H	2.60771800	2.19913100	1.10559500	H	-1.08927600	-2.17338700	-3.93970400
C	4.39312100	1.10499600	-1.67731200	C	-2.60863500	-3.46129100	0.14882900
H	5.32728500	0.56501900	-1.89277900	C	-0.91197800	4.44113100	-2.70956100
H	4.49271300	2.11537600	-2.11732000	H	-1.95196900	4.77939100	-2.84389700
H	3.58230000	0.59461800	-2.21950100	H	-0.38961200	4.59836700	-3.66286800
C	6.45492500	-1.53146700	0.10632900	H	-0.44251100	5.09607400	-1.96308900
H	6.68053200	-0.72905100	-0.61350400	C	-3.51846500	-1.76487700	2.54435700
H	7.11112800	-2.38584400	-0.14587800	H	-2.66012800	-1.93288300	3.21314700
H	6.75331300	-1.17770100	1.10709300	H	-4.22495300	-2.60089600	2.68089700
C	0.55304400	1.96998800	4.14454100	H	-4.01827200	-0.84663000	2.88850700
H	1.54400200	2.17184500	4.56249800	C	-0.52970900	1.92319500	-3.10913400
C	5.31644900	1.91090500	0.50245400	C	-0.19602100	1.91274600	-4.56577200
H	5.14331600	2.09583700	1.57387300	H	0.67229400	1.27700200	-4.78787800
H	5.49707200	2.89422700	0.02793000	H	0.04699700	2.93027500	-4.89972400
H	6.24567900	1.32799900	0.41067900	H	-1.03757600	1.55659200	-5.18123400
C	-0.59107900	2.44885900	4.78817600	C	-0.83524500	3.00090100	-2.31809200
H	-0.50586400	3.02892500	5.71129000	C	-3.89998000	-4.29312900	0.03701000
N	-0.61500300	0.79722900	-2.29164900	H	-4.51626800	-4.24201800	0.94916700
C	-4.48708000	-0.77813100	-0.20500400	H	-3.65470000	-5.35872300	-0.13195400

H	-4.52501500	-3.96978900	-0.81018500
C	-0.31484100	4.04833800	0.74473400
H	0.52306000	3.36742900	0.94210900
H	-0.62000100	4.48598100	1.70702800
H	0.03432000	4.86269700	0.09145400
C	-1.84846400	2.16673300	4.24251700
H	-2.75395700	2.52404400	4.74225400
C	-1.49354800	3.28171500	0.14723400
H	-1.77197900	2.51814700	0.87710500
C	-1.70272900	-4.10318400	1.22191800
H	-0.76568500	-3.53891500	1.35761400
H	-1.41632300	-5.12652000	0.91555300
H	-2.20315000	-4.18095200	2.19963100
C	-2.73460900	4.14509600	-0.09004900
H	-2.52075500	5.06488200	-0.65180400
H	-3.13953500	4.44559700	0.88825600
H	-3.51720400	3.58010400	-0.61813500
C	-4.54953400	0.69958100	0.23256800
H	-4.72487900	0.80594600	1.31513900
H	-5.37979400	1.22136700	-0.28066300
H	-3.62237100	1.23999800	-0.01187800
C	-1.86056100	-3.53090400	-1.19610100
H	-2.39615500	-3.00863400	-2.00374000
H	-1.74153000	-4.58407300	-1.51321800
H	-0.84482200	-3.11756800	-1.11041800
F	1.42308000	-2.87137300	-0.30801400

TS-deN-a

B3LYP(D3)/def2-SVP

E = -2646.156824 a.u.

Si	3.96734700	0.01975000	0.09457700
Al	1.72780400	-0.99787400	-0.03429400
Al	-0.61177200	-0.36937400	-0.21133400
Si	-2.53583900	-1.98399600	-0.28931000
N	-2.19751900	2.12222200	0.88588600
C	4.56160500	0.19482200	1.90278000
H	4.01339500	0.99602500	2.42335900
H	5.63865500	0.42733000	1.96013100
H	4.38285400	-0.73531100	2.46386600
C	5.18592800	-1.22867000	-0.79332300
C	5.31895200	-2.45770800	0.13343700
H	5.80974300	-2.20719400	1.08758800
H	5.93556300	-3.23496000	-0.35622200
H	4.33741900	-2.90802800	0.35409700
C	0.37849100	3.43179900	-2.62802300
H	-0.17886900	4.19667600	-3.18633700
H	1.21147900	3.09958800	-3.26400100
H	0.81441900	3.88939700	-1.72933500
C	-3.74860800	-1.54122800	-2.91615000
H	-2.78762300	-1.10036800	-3.22627800
H	-4.54088600	-1.06543200	-3.52463400
H	-3.72232000	-2.60435600	-3.19376900
C	0.24636100	-1.84354500	2.60617200
H	0.01567200	-2.78119500	2.09902900
C	-0.47614200	2.21411400	-2.27139900
H	0.20781500	1.40984600	-1.94883900
C	0.66477000	-0.72946100	1.84256000
C	4.59945000	-1.71278500	-2.13611300
H	3.64579800	-2.24477200	-1.98927700
H	5.29545200	-2.42287200	-2.62170200

H	4.42862900	-0.88894200	-2.84658100
C	2.84278200	2.62676700	0.00881300
H	1.85907800	2.17053800	-0.19107600
H	2.81571900	3.66288500	-0.37974700
H	2.96352100	2.69924800	1.10206400
C	3.76473900	1.80740600	-2.16818100
H	4.60375100	1.33428000	-2.70107400
H	3.66487600	2.83493200	-2.56796000
H	2.84787100	1.25354100	-2.43363100
C	6.58388900	-0.63224900	-1.03531900
H	6.56517400	0.17549700	-1.78425300
H	7.27244600	-1.40986700	-1.41718600
H	7.03098100	-0.22608900	-0.11268200
C	0.90297400	0.50281900	3.96007900
H	1.17994900	1.42021000	4.48732000
C	5.30929300	2.57748600	-0.34094300
H	5.50134500	2.64089500	0.74224200
H	5.26599100	3.61463000	-0.72619400
H	6.18093600	2.09457300	-0.80488300
C	0.47520600	-0.61699200	4.68199300
H	0.41310100	-0.57680600	5.77314500
N	-1.32598200	2.43725700	-1.07319600
C	-4.02871800	-1.33462100	-1.41562000
C	-5.34579100	-2.05194500	-1.05005900
H	-5.28771900	-3.14139500	-1.17994300
H	-6.16771400	-1.68876000	-1.69612100
H	-5.64354600	-1.85721800	-0.00766400
C	-1.35447000	1.56589100	-0.02571400
C	0.97393800	0.44551400	2.56555100
H	1.30337200	1.33692000	2.02295500
C	3.99370000	1.83554900	-0.64661100
C	-1.28362300	1.68163400	-3.45418400
H	-2.04744600	2.39694500	-3.79549500
H	-1.78592800	0.73915700	-3.19762800
H	-0.60472700	1.48145900	-4.29700800
C	-1.87757800	-3.73097400	-0.90849300
C	-3.74090000	4.16005700	1.10545200
H	-4.72224900	3.66220300	1.15517600
H	-3.87582400	5.10875800	0.57029700
H	-3.43938900	4.40431400	2.13332400
C	-3.29722000	-2.28289200	1.44207900
H	-2.51232600	-2.41863700	2.20114400
H	-3.93960500	-3.17926000	1.45014700
H	-3.91684000	-1.43007600	1.75734600
C	-2.18446500	3.50984400	-0.84459600
C	-2.43539900	4.63255200	-1.79887800
H	-1.60395100	5.35457100	-1.81954600
H	-3.34266700	5.17729200	-1.50661400
H	-2.58666600	4.27002200	-2.82511600
C	-2.72958300	3.31472700	0.40084400
C	-3.04411300	-4.69296600	-1.21147200
H	-3.71501200	-4.82276200	-0.34615400
H	-2.64838800	-5.69384200	-1.46571400
H	-3.65462000	-4.36516900	-2.06572800
C	-1.68565800	2.45147400	3.28985800
H	-0.65911400	2.69056200	2.98513200
H	-1.63510300	1.91609700	4.24833900
H	-2.23391400	3.39120500	3.45307100
C	0.13901100	-1.79117200	4.00017200
H	-0.19222700	-2.67263600	4.55667700

C	-2.37339100	1.56364200	2.24888100
H	-1.83696200	0.60519700	2.21166400
C	-1.01704600	-4.36906400	0.20303800
H	-0.08844200	-3.80606200	0.37403900
H	-0.70783100	-5.38475700	-0.10618400
H	-1.56354500	-4.47456900	1.15432800
C	-3.83095900	1.27554500	2.61435200
H	-4.39247900	2.18784800	2.85879700
H	-3.84815400	0.62837200	3.50354100
H	-4.35353100	0.74836600	1.80637200
C	-4.24860700	0.17340000	-1.18193300
H	-4.41306600	0.41263800	-0.12115300
H	-5.14179900	0.52088900	-1.73477800
H	-3.39970100	0.78010700	-1.52687200
C	-0.98415400	-3.59801000	-2.15950000
H	-1.50415900	-3.13660700	-3.01201800
H	-0.64594700	-4.59882800	-2.48721900
H	-0.07961400	-3.00936100	-1.94390000
F	1.86687800	-2.71045900	-0.18681800

P-deN-a

B3LYP(D3)/def2-SVP

E = -2646.163119 a.u.

Si	3.89743100	0.01333300	-0.18914600
Al	1.76250500	-1.03881400	0.49155800
Al	-0.63502200	-0.33691700	-0.28423300
Si	-2.58552700	-1.92872300	-0.47722900
N	-2.04319300	2.01414100	1.05464300
C	5.11512300	0.12086600	1.28784200
H	4.85320000	0.95638100	1.95754000
H	6.15345300	0.28082000	0.95129200
H	5.09028800	-0.80034800	1.89041300
C	4.73900200	-1.16899900	-1.50529500
C	5.12734200	-2.46195000	-0.75371500
H	5.88149400	-2.27835900	0.02847300
H	5.56235000	-3.19450500	-1.45996000
H	4.24908000	-2.93715200	-0.28771300
C	0.08584600	3.58985600	-2.62714000
H	-0.41466500	4.54061900	-2.85562000
H	0.67674600	3.30655900	-3.50942000
H	0.78239000	3.73918600	-1.79166700
C	-3.67234100	-1.22788700	-3.05722600
H	-2.73021500	-0.67578500	-3.21410000
H	-4.45219700	-0.73039400	-3.66440900
H	-3.52767000	-2.23532400	-3.47560900
C	0.34959300	-1.82279600	3.02439000
H	-0.18620200	-2.52543100	2.37765200
C	-0.91267500	2.47421200	-2.31620400
H	-0.33980400	1.53149300	-2.27774300
C	1.34249900	-0.98359900	2.46740900
C	3.74735400	-1.55773300	-2.62162200
H	2.86139000	-2.06486200	-2.20863600
H	4.22717600	-2.26004100	-3.32988200
H	3.40680400	-0.69082300	-3.20848200
C	2.70941900	2.57319800	0.12963800
H	1.70252600	2.12467100	0.07685100
H	2.61335700	3.64577200	-0.13158600
H	3.03308600	2.52740000	1.18320200
C	3.15092600	1.91721400	-2.24709200
H	3.85036600	1.47926100	-2.97453200

H	2.97500800	2.96336200	-2.55966100
H	2.19523000	1.37266200	-2.33677300
C	6.00500400	-0.55935900	-2.13319600
H	5.77840900	0.31699800	-2.76111600
H	6.50858000	-1.30014700	-2.78340900
H	6.73831900	-0.24519000	-1.37194400
C	1.72215500	-0.11441700	4.74006700
H	2.27384500	0.55314700	5.40912900
C	5.04105600	2.63378800	-0.77166700
H	5.45466800	2.68487600	0.24758000
H	4.90003200	3.67607600	-1.11872200
H	5.80573800	2.17562500	-1.41666100
C	0.73132900	-0.95650900	5.25647700
H	0.50330000	-0.95089500	6.32618500
N	-1.48355900	2.59146900	-0.95487700
C	-4.07833100	-1.27331400	-1.57250400
C	-5.34243800	-2.14377000	-1.41614400
H	-5.19424200	-3.17509200	-1.76193200
H	-6.17359900	-1.71681000	-2.00903000
H	-5.68162100	-2.18765200	-0.36870100
C	-1.44049700	1.56247700	-0.07151500
C	2.01260200	-0.12960500	3.37024300
H	2.79735900	0.53774000	3.00044000
C	3.70538800	1.86621500	-0.81400200
C	-1.99672100	2.33406500	-3.38696100
H	-2.57926700	3.26082700	-3.49806400
H	-2.69143900	1.51729800	-3.15375100
H	-1.52612100	2.11806800	-4.35811700
C	-1.84211600	-3.63052800	-1.09714700
C	-3.18756500	4.13340300	1.92614600

H	-4.21271700	3.76923800	2.09828600
H	-3.25386700	5.18250500	1.61011000
H	-2.66050300	4.11288200	2.89082900
C	-3.33412800	-2.24660700	1.25759100
H	-2.55589200	-2.36373300	2.02709900
H	-3.93807700	-3.16964100	1.25127500
H	-3.99321400	-1.42169600	1.56727500
C	-2.13332600	3.69282600	-0.39120300
C	-2.37061200	4.99944700	-1.07675600
H	-1.45042700	5.60032100	-1.15340700
H	-3.10646400	5.58886700	-0.51429800
H	-2.76695500	4.86579400	-2.09313400
C	-2.47678800	3.32837200	0.88757100
C	-2.93194600	-4.60916600	-1.57369700
H	-3.69952600	-4.78897800	-0.80267500
H	-2.47797700	-5.58856500	-1.81314700
H	-3.44155900	-4.26085300	-2.48532900
C	-1.00188200	1.66359800	3.27730500
H	-0.01616500	1.70346600	2.79531500
H	-0.92672200	0.94767700	4.10726300
H	-1.24032000	2.65554600	3.69103200
C	0.04139000	-1.81309200	4.39044700
H	-0.72884600	-2.48461400	4.78259100
C	-2.06746100	1.19500400	2.28994400
H	-1.78400200	0.18526000	1.94903400
C	-1.08792300	-4.28312300	0.08171000
H	-0.25554900	-3.65923300	0.43790300
H	-0.63464800	-5.23548100	-0.24945000
H	-1.75490200	-4.51706400	0.92684000
C	-3.46074000	1.07531400	2.90785900

H	-3.74514600	1.97309900	3.47371100
H	-3.46178300	0.22872700	3.60954800
H	-4.22379900	0.88350000	2.14000300
C	-4.44606200	0.15147200	-1.10948600
H	-4.74748400	0.17516300	-0.04947100
H	-5.30028400	0.53829200	-1.69640500
H	-3.61647300	0.86172000	-1.22821300
C	-0.82954700	-3.39845700	-2.23897500
H	-1.26792100	-2.85755700	-3.09358300
H	-0.45950100	-4.36743500	-2.62184500
H	0.05576000	-2.84794900	-1.88240000
F	1.76373100	-2.68567700	-0.04212900

INT-deN-b

B3LYP(D3)/def2-SVP

E = -2646.173261 a.u.

Si	3.45358400	-0.42432400	-0.76721700
Al	1.35025900	0.88114700	-0.35941200
Al	-1.04239000	0.03984500	-0.95749400
Si	-3.07516900	1.47586700	-0.89633400
N	-1.29565100	-2.98828600	-0.23752900
C	3.31465900	-1.02735000	-2.58026500
H	2.53830400	-1.80382500	-2.67114300
H	4.26463200	-1.45375400	-2.94388300
H	3.03110200	-0.20951800	-3.26118500
C	5.03396200	0.72545100	-0.66851900
C	4.90033200	1.77265300	-1.79528700
H	4.91683100	1.31270100	-2.79632200
H	5.74130500	2.48952000	-1.75052400
H	3.97434600	2.36319300	-1.70261700
C	0.46647800	-0.30335500	2.86094900
H	0.86120700	-1.09301000	3.51618200
H	0.49108000	0.64546500	3.41598400
H	1.15701500	-0.21632900	2.00792500
C	-4.74724800	0.06485300	0.99853900
H	-3.83296900	-0.46009600	1.31879300
H	-5.60202600	-0.60582600	1.21067500
H	-4.86624500	0.95471000	1.63650100
C	-0.96444600	-0.58765000	2.40065700
H	-1.27046100	0.22896600	1.73471800
C	5.10231700	1.47501200	0.67736900
H	4.20702100	2.09075200	0.85077200
H	5.97452700	2.15551400	0.68996700
H	5.21415100	0.79194400	1.53300100
C	2.22417000	-2.74059400	0.36677300
H	1.47884900	-2.12255500	0.88466100
H	2.28466800	-3.70133900	0.91270300
H	1.82271900	-2.96050400	-0.63296900
C	4.06691200	-1.76094000	1.74485000
H	5.10417300	-1.39476500	1.77815500
H	4.02576600	-2.67634100	2.36558800
H	3.43401400	-1.00275000	2.23564700
C	6.34634000	-0.05254500	-0.87874100
H	6.55727700	-0.74073700	-0.04538900
H	7.20032800	0.64808200	-0.94093200
H	6.34089200	-0.64191700	-1.81050100
C	4.58965700	-3.06690800	-0.33296800
H	4.26824400	-3.36757400	-1.34260400
H	4.64536300	-3.98798200	0.27834600
H	5.61042800	-2.66753600	-0.41324000

N	-1.05673300	-1.79180000	1.54872600
C	-4.68555100	0.41961400	-0.49646900
C	-5.98523300	1.13654900	-0.91005600
H	-6.14959700	2.06918000	-0.35270200
H	-6.85998300	0.48480000	-0.72095200
H	-5.99141200	1.38362800	-1.98356900
C	-1.23668200	-1.70419600	0.20162100
C	3.60765300	-2.06475100	0.30727100
C	-1.95311900	-0.60297400	3.56947600
H	-1.61949400	-1.24885400	4.39329300
H	-2.95026800	-0.93013100	3.24395700
H	-2.04619100	0.41868600	3.96679000
C	-2.88621300	3.07830100	0.21885500
C	-1.06778700	-5.37012100	0.69552100
H	-2.07483400	-5.80859500	0.61056800
H	-0.59250200	-5.80604600	1.58474800
H	-0.48782300	-5.69252600	-0.17993600
C	-3.32102900	2.09684600	-2.68819800
H	-2.39716800	2.55094100	-3.07882800
H	-4.12578700	2.84800100	-2.76158800
H	-3.57328300	1.25602000	-3.35352600
C	-0.95844700	-3.12317600	1.95175000
C	-0.74253200	-3.58752400	3.35502300
H	0.05257400	-3.02163200	3.86055000
H	-0.44448900	-4.64446200	3.35617900
H	-1.65498300	-3.50276300	3.96685700
C	-1.10484700	-3.88141500	0.81807200
C	-4.21830500	3.81289100	0.45293800
H	-4.73255400	4.04973800	-0.49271800
H	-4.04018000	4.77144200	0.97651200

H	-4.91320900	3.22917800	1.07697300
C	-0.29649600	-3.76177600	-2.39116000
H	0.43308500	-2.94103600	-2.38720700
H	-0.54041900	-3.98325900	-3.44113100
H	0.16704800	-4.65814400	-1.95160600
C	-1.56697900	-3.33244100	-1.65847100
H	-1.87227100	-2.38330700	-2.10797200
C	-1.92507700	4.03357500	-0.51900800
H	-0.97232100	3.54572000	-0.77513800
H	-1.67338400	4.89923200	0.12133100
H	-2.36729100	4.42718200	-1.44748500
C	-2.73269800	-4.31261000	-1.80998700
H	-2.46074400	-5.35142500	-1.57736800
H	-3.07277600	-4.29088100	-2.85636300
H	-3.58273900	-4.01723900	-1.17636700
C	-4.59387500	-0.89578100	-1.29819100
H	-4.49996300	-0.72113700	-2.38219200
H	-5.50138200	-1.50962200	-1.14104300
H	-3.73159300	-1.50295700	-0.98464100
C	-2.25674700	2.73814700	1.58379600
H	-2.85197000	2.00445300	2.15093300
H	-2.17136600	3.64636000	2.20941900
H	-1.23370400	2.34683100	1.46362800
C	1.62679500	2.76832100	0.23065000
C	1.66294100	3.17501900	1.58126400
C	1.72972300	3.79318600	-0.73554000
H	1.59745500	2.42910900	2.38072600
C	1.78711000	4.51904500	1.95011900
C	1.86090600	5.13987400	-0.38105000
H	1.69707400	3.54714600	-1.80424000

C	1.88653900	5.50888300	0.96746500
H	1.80811700	4.79567500	3.00845100
H	1.93735900	5.90465400	-1.15935900
H	1.98357600	6.56041600	1.25055300
F	-0.96396100	-0.60615700	-2.56300500

TS-deN-b

B3LYP(D3)/def2-SVP

E = -2646.162948 a.u.

Si	3.11271300	1.24064600	0.59172500
Al	0.88585400	0.10830400	0.39040700
Al	-1.45865700	0.92431400	-0.13019500
Si	-3.56047100	-0.19438500	0.58425900
N	1.43795900	-2.76559000	1.06088700
F	-0.67310000	0.22302900	-1.59549200
N	1.14321700	-2.69198800	-1.08332100
C	1.24178800	-1.90930300	0.02233400
C	4.10636400	0.45151500	2.02990500
H	3.60888800	0.62290200	2.99865700
H	5.11803100	0.88611900	2.09686100
H	4.22109400	-0.63446400	1.89646300
C	4.18578500	0.99459400	-1.02439600
C	4.72325500	-0.45129200	-1.00127700
H	5.48681600	-0.59046100	-0.21979000
H	5.19707600	-0.70560300	-1.96801000
H	3.92498400	-1.18900600	-0.81512800
C	1.51231100	-2.27170000	2.45283500
H	1.68287500	-1.18685000	2.34222500
C	-5.54046100	1.13229400	-1.06911000
H	-4.68729300	1.49294200	-1.66832900
H	-6.34574500	1.88564800	-1.15803600
H	-5.91351700	0.20595200	-1.53356100
C	3.30030800	1.18766600	-2.27173400
H	2.45121800	0.48571900	-2.28904900
H	3.88296100	1.01560700	-3.19641700
H	2.87886000	2.20262400	-2.32967900
C	1.75938100	3.13496600	2.20898100
H	0.78296800	2.77713100	1.84836400
H	1.60943800	4.17074900	2.56544200
H	2.04653100	2.52803500	3.08510800
C	2.32199300	3.93187700	-0.09586000
H	3.08222700	4.02971400	-0.88701600
H	2.05694700	4.95434500	0.22821900
H	1.41440800	3.50155200	-0.54518100
C	5.38708300	1.95377100	-1.10958500
H	5.07243700	3.00246500	-1.21767000
H	6.01149200	1.70923200	-1.98988800
H	6.03633600	1.88592300	-0.22120600
C	1.43158300	-4.08872100	0.62140200
C	0.99853500	-2.13366500	-2.45261900
H	0.81062600	-1.06826700	-2.28358600
C	1.25163500	-4.04097600	-0.73828300
C	2.71598300	-2.81685900	3.22390000
H	2.55348700	-3.84400400	3.57785400
H	2.88913000	-2.18649200	4.10860000
H	3.62683500	-2.79348400	2.60831800
C	4.11759900	3.74836700	1.65775800
H	4.48800100	3.22473500	2.55403600
H	3.91589300	4.79471600	1.95426100
H	4.93486500	3.77244800	0.92222800

C	-5.15946400	0.92463000	0.40780800
C	-6.36733800	0.34235500	1.16829000
H	-6.67973700	-0.63844400	0.78062000
H	-7.23712500	1.02077400	1.07895400
H	-6.15884500	0.22470500	2.24379200
C	2.83227300	3.10508000	1.09891600
C	-3.67930100	-1.90811800	-0.36883200
C	-3.46948700	-0.63259400	2.44789800
H	-2.50474300	-1.09078300	2.71111700
H	-4.26841600	-1.33209500	2.74623300
H	-3.57484300	0.27485600	3.06435800
C	-4.96794200	-2.68769600	-0.05404900
H	-5.11677200	-2.83344400	1.02848500
H	-4.93615400	-3.69217400	-0.51892800
H	-5.86044100	-2.17907200	-0.44941500
C	-0.23349600	-2.67253700	-3.18069100
H	-0.07347400	-3.67525900	-3.60202500
H	-0.47368400	-1.99624600	-4.01405600
H	-1.10375500	-2.69629000	-2.51555400
C	0.18080900	-2.46681900	3.17843100
H	-0.63847700	-2.01602100	2.60159900
H	0.21581800	-1.98203300	4.16568700
H	-0.04434400	-3.53302300	3.33099800
C	-2.47076400	-2.76004600	0.07669500
H	-1.51371600	-2.24658700	-0.10632500
H	-2.44319200	-3.71586900	-0.48188700
H	-2.51519600	-3.01321500	1.14826500
C	2.28336100	-2.28807300	-3.26864300
H	3.15057500	-1.88328500	-2.73318400
H	2.17740100	-1.72992500	-4.21127500
H	2.49042600	-3.33765900	-3.52622000
C	1.13685300	-5.18416400	-1.69366000
H	0.09956100	-5.32225400	-2.03718500
H	1.45476400	-6.11520000	-1.20653600
H	1.76585500	-5.04688900	-2.58385000
C	-4.82667800	2.30300100	1.01812000
H	-4.50108100	2.22831800	2.06957000
H	-5.72209700	2.95274100	1.00394800
H	-4.03575300	2.82332600	0.45842000
C	1.60087600	-5.28644100	1.49868400
H	2.63348200	-5.38740900	1.86913700
H	1.35839500	-6.19849400	0.93813700
H	0.93450900	-5.25381800	2.37295700
C	-3.58733000	-1.68726800	-1.89185500
H	-4.45544700	-1.13750300	-2.28287400
H	-3.55709900	-2.65900200	-2.42212000
H	-2.68408300	-1.12392500	-2.17295700
C	-1.35501800	2.88547900	-0.46937900
C	-1.01370300	3.33203000	-1.76489800
C	-1.51305000	3.87961200	0.51938700
H	-0.86029500	2.59597800	-2.56137500
C	-0.84781000	4.69037000	-2.06045700
C	-1.34122700	5.23915800	0.23957000
H	-1.77436500	3.59162100	1.54431300
C	-1.00796500	5.64986100	-1.05602300
H	-0.58485700	5.00195000	-3.07608100
H	-1.46498200	5.98203000	1.03337500
H	-0.87140400	6.71166000	-1.27964600

P-deN-b

B3LYP(D3)/def2-SVP

E = -2646.168804 a.u.

Si	-3.05620700	1.11591500	-0.80169900
Al	-0.86649100	0.07896200	-0.15702500
Al	1.48800200	0.93051400	0.71694600
Si	3.34159000	-0.00400200	-0.66545500
N	-1.39613600	-2.89619500	-0.84438400
F	1.45663300	0.23811100	2.31665300
N	-0.94350300	-2.66077400	1.25998900
C	-1.18171000	-1.96275200	0.12025500
C	-4.03837200	0.27677400	-2.21354800
H	-3.50482300	0.36053700	-3.17480700
H	-5.02267500	0.75763300	-2.34052400
H	-4.21568300	-0.78992700	-2.01602100
C	-4.15797300	0.96682300	0.81131300
C	-4.65188300	-0.49202600	0.90819300
H	-5.37622200	-0.73432900	0.11445000
H	-5.15886000	-0.66574000	1.87589000
H	-3.82478300	-1.21809800	0.83229300
C	-1.66392600	-2.51201100	-2.24743500
H	-1.87280000	-1.43292500	-2.18401500
C	5.77228700	0.96845000	0.58391500
H	5.14483300	1.18563500	1.46473900
H	6.60889100	1.69255300	0.58894100
H	6.21154300	-0.03181700	0.72217300
C	-3.30821200	1.28608800	2.05856200
H	-2.44187600	0.60740100	2.15738200
H	-3.90560100	1.16652900	2.98181200
H	-2.91043400	2.31087100	2.04994600
C	-1.55792100	2.90321200	-2.39445600
H	-0.61767400	2.57850800	-1.91999200
H	-1.37052500	3.91051700	-2.80978400
H	-1.76450000	2.23306900	-3.24730200
C	-2.32741400	3.85231300	-0.20748900
H	-3.16130500	4.00615900	0.49489000
H	-2.02534500	4.84891800	-0.57455400
H	-1.47027600	3.45766400	0.35848400
C	-5.38411200	1.89711800	0.78324200
H	-5.09771200	2.95925600	0.79897000
H	-6.02451400	1.71898300	1.66794200
H	-6.00873100	1.73012800	-0.10978800
C	-1.27357800	-4.18270700	-0.31980300
C	-0.69573300	-1.98212800	2.55732900
H	-0.47861300	-0.94045700	2.27735000
C	-1.00010800	-4.03052800	1.01797900
C	-2.92521600	-3.17210900	-2.80952000
H	-2.75633600	-4.21847700	-3.09870800
H	-3.24350800	-2.62712300	-3.71018900
H	-3.74932200	-3.13492200	-2.08187400
C	-3.95735900	3.54843500	-2.09611100
H	-4.23249600	2.98567500	-3.00262700
H	-3.74067600	4.58592100	-2.41181600
H	-4.84302900	3.58599000	-1.44357200
C	4.97769100	1.07578200	-0.73032900
C	5.89056200	0.68510900	-1.90961000
H	6.24758700	-0.35308800	-1.84448600
H	6.78472300	1.33714800	-1.93238100
H	5.38155100	0.80192200	-2.87988500
C	-2.72729600	2.94672200	-1.38694900
C	3.67655800	-1.85534400	-0.08351000

C	2.74966700	-0.11882000	-2.48944500
H	1.70895200	-0.47655400	-2.54732200
H	3.37457400	-0.79118300	-3.10138000
H	2.77066000	0.87721400	-2.96174100
C	4.85500300	-2.51995500	-0.81655900
H	4.74290700	-2.47986100	-1.91291600
H	4.93405300	-3.58787900	-0.53412500
H	5.81614800	-2.04876400	-0.56108200
C	0.56069100	-2.48355600	3.27043000
H	0.37725200	-3.39106600	3.86339500
H	0.91223100	-1.68875000	3.94146400
H	1.37099200	-2.67282500	2.55534500
C	-0.43512700	-2.70581500	-3.13714100

TS1a

B3LYP(D3)/def2-SVP

E = -3186.387057 a.u.

Si	-2.38724600	-2.75393300	0.13565200
Al	-1.50336600	-0.50166900	-0.64206300
Al	1.00785700	0.33914100	-0.03992800
Si	1.83853200	2.73951200	0.42131000
N	-3.10427100	1.43660600	1.34679400
N	-3.59850700	1.75317500	-0.73423400
N	2.41235400	-1.08516700	2.30544800
C	-2.91232300	0.91518000	0.10017600
C	-2.70443200	-3.14436000	2.00103100
H	-1.76397600	-3.37021300	2.52612400
H	-3.35379500	-4.02937800	2.11269800
H	-3.18895700	-2.31329300	2.53175300
C	-4.18226800	-2.97203600	-0.67182300
C	-5.13961400	-2.03323800	0.09422300
H	-5.25527000	-2.33245300	1.14883200
H	-6.14900900	-2.05208500	-0.36049400
H	-4.78934300	-0.98844600	0.08125500
C	3.94317000	-2.91558000	-1.54118900
H	4.71184000	-3.60353900	-1.16432400
H	4.11258700	-2.79153300	-2.61920200
H	2.95123800	-3.36595000	-1.40673400
C	-2.67118800	0.72135100	2.56012400
H	-2.12133400	-0.14535100	2.16295900
C	4.04676600	2.57345200	-1.46511700
H	3.80051100	1.51110800	-1.60128300
H	5.12382100	2.69628100	-1.68831600
H	3.49087500	3.12867900	-2.23542800
C	4.01709000	-1.54022300	-0.87556000
H	3.29496900	-0.88768000	-1.38228000
C	-4.19004600	-2.56356600	-2.15683100
H	-3.79712000	-1.54644300	-2.30684100
H	-5.22015500	-2.59130200	-2.56301200
H	-3.57465900	-3.23279600	-2.77515400
C	0.27273000	-3.78195800	0.03592700
H	0.59604800	-2.83676400	-0.42128700
H	1.00554600	-4.55934300	-0.25714100
H	0.34712300	-3.66910800	1.13047700
C	-1.15753200	-4.36743800	-1.93829500
H	-2.10158500	-4.81413800	-2.28793300
H	-0.34123000	-5.04444100	-2.25577700
H	-1.01350200	-3.41319500	-2.46585600
C	-4.72838100	-4.40744500	-0.56121000
H	-4.14732500	-5.11624800	-1.17058500
H	-5.77385100	-4.45194700	-0.92389800
H	-4.73029800	-4.77918300	0.47617600
C	-3.87521600	2.60078000	1.29450200
C	-3.66097100	1.51874900	-2.19156500
H	-2.95529600	0.67304200	-2.33083700
C	-4.18975900	2.79554800	-0.02530100
C	-3.86822900	0.18506400	3.35049600
H	-4.44726700	0.98628000	3.83296100
H	-3.51405400	-0.49256300	4.14215300
H	-4.53994800	-0.38156700	2.69002700

C	-1.47052800	-5.54518600	0.25146200
H	-1.40750500	-5.49809600	1.35035500
H	-0.74469000	-6.31248700	-0.08158100
H	-2.47337300	-5.91493300	-0.00705000
N	3.54225700	-1.57284300	0.52466500
C	3.72668600	3.07283400	-0.04670700
C	4.12435900	4.55735000	0.04614200
H	3.62020500	5.16889400	-0.71779600
H	5.21324200	4.67074300	-0.11689200
H	3.89762300	4.99307100	1.03261300
C	2.52426800	-0.78441700	0.97658400
C	-1.14845000	-4.18941000	-0.40891500
C	5.39667400	-0.89042800	-1.01619500
H	6.20268900	-1.53290700	-0.63103300
H	5.43944700	0.07343200	-0.49471900
H	5.59539500	-0.70760400	-2.08319500
C	0.60746300	4.15069600	-0.20997400
C	3.49451200	-2.61813900	4.05801900
H	3.93031600	-1.88437300	4.75483300
H	4.16048400	-3.49040700	4.03462800
H	2.53721300	-2.95187500	4.48360100
C	1.83041800	2.99750700	2.32925200
H	0.81312500	3.03736400	2.74584000
H	2.32503800	3.94935100	2.58482500
H	2.37687300	2.20013800	2.84914400
C	4.05762100	-2.36378400	1.55545200
C	5.16826500	-3.35561900	1.42017700
H	4.83909300	-4.27689300	0.91335900
H	5.54108600	-3.63847300	2.41349400
H	6.01918500	-2.95213700	0.85484800
C	3.33618500	-2.06142600	2.67967000
C	0.84926600	5.50417200	0.49026200
H	0.77474800	5.42550800	1.58608100
H	0.08229000	6.23403100	0.16736800
H	1.82844300	5.94050200	0.25137400
C	0.21158600	-1.54285600	3.32051200
H	-0.21370200	-1.79631700	2.34105100
H	-0.59168000	-1.12948900	3.94586600
H	0.56611200	-2.46643600	3.80088600
C	-3.09162800	2.68141500	-3.00394700
H	-3.74567000	3.56519300	-3.01530800
H	-2.95915100	2.35384900	-4.04660300
H	-2.10473300	2.97581900	-2.61742900
C	-1.70210900	1.54232400	3.40868700
H	-0.84054700	1.85853900	2.80682200
H	-1.33311100	0.93100200	4.24505700
H	-2.16915400	2.43701600	3.84210600
C	1.33750900	-0.52492300	3.14779600
H	0.94026700	0.31320300	2.55581300
C	-0.82497100	3.69921900	0.13135900
H	-1.07915200	2.74299100	-0.34769600
H	-1.56132100	4.44982200	-0.21304900
H	-0.97831000	3.57843400	1.21504400
C	-5.05039100	1.06714000	-2.63950800
H	-5.41174500	0.23796900	-2.01508600
H	-5.00044700	0.70854200	-3.67944900
H	-5.79317900	1.87989200	-2.60306600
C	1.84132300	0.05823500	4.47106800
H	1.98875900	-0.71417700	5.23808600
H	1.09448600	0.76751200	4.85724500
H	2.78610300	0.60431600	4.33638100
C	-4.94665100	3.93051700	-0.63461500
H	-4.27035900	4.66943300	-1.09572200
H	-5.53094600	4.45343600	0.13471400
H	-5.64671400	3.59272100	-1.41042500
C	4.59141000	2.28546600	0.96174400
H	4.54803700	2.72256000	1.97137600
H	5.65288100	2.29850400	0.64985000
H	4.27930900	1.23246400	1.04411000
C	-4.17764800	3.49964600	2.44926300
H	-4.52422500	2.95404800	3.33761600
H	-4.96621600	4.21284300	2.17344300
H	-3.29314200	4.08965300	2.74382800
C	0.68520400	4.36306400	-1.73133400

H	1.69566500	4.63842500	-2.07245300
H	0.00151700	5.17529100	-2.04320700
H	0.37456400	3.46115500	-2.27976300
C	1.21547900	-0.05186900	-2.72821100
C	1.72666200	1.10070400	-3.37658000
C	2.63727400	1.01553800	-4.42819000
C	3.03930100	-0.23578800	-4.91645000
C	2.51276700	-1.40332000	-4.33610000
C	1.62917200	-1.28357300	-3.27645600
H	1.40160800	2.08081300	-3.03106500
H	3.03327700	1.93013300	-4.87737300
H	3.73927600	-0.31022700	-5.75177600
H	2.77901300	-2.39900800	-4.69728600
F	1.14421500	-2.41546800	-2.72031300
H	0.18301700	-0.04385400	-2.22228400

INT1a

B3LYP(D3)/def2-SVP

E = -3186.497397 a.u.

Si	-2.60380500	2.56886900	-0.33567700
Al	-1.37200000	0.46395100	0.33609800
Al	1.16825800	-0.37018900	0.20872600
Si	2.16757000	-2.46236700	-0.90608500
N	-2.99737700	-1.65277000	-1.25470700
N	-2.93885200	-2.17208100	0.84477100
N	2.21576700	1.81335000	-1.72939000
C	-2.58918000	-1.19651100	-0.03913700
C	-2.93311300	3.01242100	-2.18368200
H	-2.04513200	3.45965500	-2.65479500
H	-3.74672300	3.75198000	-2.26532900
H	-3.22428800	2.14046600	-2.79008700
C	-4.42484600	2.46125800	0.43010200
C	-5.22150900	1.46143100	-0.43484500
H	-5.33257700	1.80339000	-1.47652500
H	-6.24137600	1.32547900	-0.02679300
H	-4.74260400	0.46909000	-0.45125800
C	3.57164500	2.84983900	2.56997600
H	4.31171600	3.65990000	2.50279400
H	3.68286700	2.38119100	3.55947000
H	2.56082000	3.27237900	2.51743200
C	-2.75609300	-0.87550200	-2.48745400
H	-2.48713600	0.12188000	-2.11297900
C	4.41510800	-2.33004800	0.94407000
H	3.99381800	-1.38386900	1.31436200
H	5.50797200	-2.29931900	1.11721900
H	4.01168400	-3.13480200	1.57759700
C	3.77431500	1.77412800	1.49850200
H	3.22687700	0.88163500	1.81744100
C	-4.41758600	1.92829400	1.87523900
H	-3.94636400	0.93782400	1.94134600
H	-5.45368000	1.83914400	2.25604800
H	-3.86809900	2.58627200	2.56385400
C	-0.11928600	3.96112100	-0.10616200
H	0.34430200	3.04486800	0.28605300
H	0.48577400	4.81519200	0.25558700
H	-0.02442200	3.94636500	-1.20373900
C	-1.61475000	4.15297100	1.88823200
H	-2.62129000	4.37900800	2.27368100
H	-0.94006400	4.94657300	2.26418100
H	-1.28339700	3.20110700	2.32862300
C	-5.16142100	3.81327800	0.41201800
H	-4.70425300	4.53784700	1.10295600
H	-6.21344300	3.68266700	0.73167100
H	-5.18256300	4.27159800	-0.59001800
C	-3.56952000	-2.92083100	-1.14623300
C	-2.73184300	-2.02525200	2.30676000
H	-2.21440700	-1.05836300	2.38918000
C	-3.53583600	-3.24671600	0.18537200
C	-4.00614200	-0.70561700	-3.35267100
H	-4.25719000	-1.61221900	-3.92022700
H	-3.82398200	0.09750700	-4.08289800
H	-4.87228500	-0.41394600	-2.74196400

C	-2.10740500	5.45880400	-0.19870700
H	-2.05961800	5.50587600	-1.29863100
H	-1.48940700	6.29226700	0.18820300
H	-3.14640100	5.66281300	0.09677600
N	3.16407200	2.10942200	0.19077800
C	4.10973900	-2.55882900	-0.54728000
C	4.74882500	-3.89178300	-0.97690600
H	4.39079500	-4.73682100	-0.36903900
H	5.84811900	-3.84857300	-0.85285700
H	4.55207200	-4.13026900	-2.03458600
C	2.35637900	1.25006400	-0.49479800
C	-1.58673000	4.11488200	0.34844800
C	5.24390500	1.37503700	1.33250600
H	5.87503800	2.22294000	1.02502700
H	5.35496800	0.57237200	0.59187600
H	5.62797000	1.00349000	2.29500600
C	1.23272400	-4.16176800	-0.49989400
C	2.89250800	3.95480900	-2.98303300
H	3.58536200	3.61562600	-3.76979800
H	3.21271100	4.95766100	-2.67128300
H	1.89755900	4.05897300	-3.43620700
C	2.10316400	-2.43521600	-2.82971800
H	1.07727600	-2.54532200	-3.21204900
H	2.69590500	-3.26936400	-3.23906100
H	2.52011000	-1.51331500	-3.25116100
C	3.48028600	3.22353300	-0.58547500
C	4.26789400	4.40239600	-0.11429500
H	3.71923000	4.97306900	0.65141000
H	4.47160400	5.07890400	-0.95448400
H	5.23691200	4.11430400	0.31749100
C	2.87751000	3.03867600	-1.80145100
C	1.58181400	-5.28625400	-1.49732500
H	1.32724900	-5.01851500	-2.53416700
H	1.00503900	-6.19835300	-1.25033300
H	2.64556800	-5.56146500	-1.47441300
C	0.15970100	1.79889700	-3.14685200
H	-0.47757400	1.79551700	-2.25550800
H	-0.35233900	1.23462100	-3.93937900
H	0.26962200	2.83499200	-3.49623500
C	-1.80362900	-3.08896900	2.89203700
H	-2.26773600	-4.08485200	2.94751600
H	-1.52327400	-2.78975900	3.91314800
H	-0.87445000	-3.16260400	2.31187500
C	-1.55242900	-1.41930300	-3.25524600
H	-0.66027800	-1.39587300	-2.61600500
H	-1.36205700	-0.80015200	-4.14438200
H	-1.70973900	-2.45374900	-3.59564000
C	1.49718400	1.13997200	-2.83179800
H	1.27978000	0.14132300	-2.43158200
C	-0.27815100	-3.88821300	-0.61950000
H	-0.60716200	-3.08366500	0.05156100
H	-0.86230200	-4.79415800	-0.36813800
H	-0.56536600	-3.59568800	-1.64079000
C	-4.06100900	-1.89389200	3.05391400
H	-4.69728800	-1.12739500	2.58800600
H	-3.86077400	-1.58189500	4.09049500
H	-4.62482800	-2.83835400	3.09609300
C	2.38583400	0.95858500	-4.06547000
H	2.51108000	1.89219100	-4.63075100
H	1.91851000	0.22676000	-4.74058500
H	3.38030100	0.57989200	-3.78634000
C	-3.95646600	-4.52489800	0.83418500
H	-3.08409100	-5.13232400	1.12554900
H	-4.56070000	-5.12203100	0.13856400
H	-4.56068700	-4.35825200	1.73610500
C	4.77523400	-1.42679100	-1.36012200
H	4.73149000	-1.61629400	-2.44420900
H	5.84474300	-1.33466200	-1.09106900
H	4.30207300	-0.44913800	-1.17487300
C	-4.08643600	-3.73434400	-2.28785100
H	-5.03488000	-3.34083600	-2.68729200
H	-4.26722800	-4.76637600	-1.95983400
H	-3.36786000	-3.77811000	-3.11938100
C	1.52016300	-4.67634300	0.92045800

H	2.58696500	-4.89826900	1.07929000
H	0.96087000	-5.61207700	1.11232000
H	1.20907000	-3.95631100	1.68964300
C	1.38591200	-0.42096200	2.24916500
C	1.67935500	-1.56035400	3.02723500
C	1.57535000	-1.58508700	4.42472100
C	1.17284100	-0.43850400	5.11483000
C	0.88568300	0.72620300	4.39487900
C	1.00425300	0.69060900	3.01021000
H	2.01004400	-2.46924900	2.52602600
H	1.81419700	-2.50088000	4.97257500
H	1.08706300	-0.44269600	6.20465400
H	0.57050600	1.65022900	4.88393200
H	-1.55776400	0.59223700	1.93261100
F	0.74449500	1.85826300	2.35127600

TS-ben

B3LYP(D3)/def2-SVP

E = -1659.609861 a.u.

Al	-0.25218800	0.71844000	-0.51694600
Si	-2.14120400	-0.46513600	0.74698300
N	2.48246400	0.42841600	0.90585600
C	1.47237700	-0.21811300	0.26124100
C	2.26287900	1.76796000	1.48425600
H	1.32555600	2.10325900	0.99918300
C	0.04677500	0.79069000	-3.18142200
H	-0.11556200	-0.17889100	-3.66389700
C	3.62018300	-0.37791800	0.98534500
C	-0.98039600	1.79687200	-3.31798900
H	-1.91763400	1.56195200	-3.82986300
C	1.39934400	1.27482800	-3.05360500
H	2.24486500	0.65955600	-3.37549300
C	3.33775800	2.78137600	1.08933200
H	4.27769600	2.64450300	1.64250500
H	2.97073100	3.79498500	1.31106100
H	3.54428400	2.72507800	0.01087800
C	2.02116800	1.69131100	2.99272000
H	1.23328200	0.95974600	3.22334000
H	1.69779300	2.67331800	3.37136500
H	2.93346300	1.40348800	3.53897500
C	-2.95503600	0.90020700	1.90297200
C	-1.92423600	1.20136900	3.01153600
H	-1.71231100	0.32078000	3.63921400
H	-2.29507700	2.00075500	3.68075000
H	-0.96593900	1.55292300	2.59141100
C	4.90543800	0.01643900	1.63773200
H	4.74434300	0.42872200	2.64493400
H	5.45245300	0.77439400	1.05395000
H	5.56015300	-0.85882200	1.74303600
C	-4.26567500	0.43161900	2.55959600
H	-5.06957400	0.30320800	1.81818100
H	-4.62100800	1.17898300	3.29502900
H	-4.14855700	-0.52381700	3.09679400
C	-3.22097200	2.21199400	1.13730800
H	-2.30683900	2.60770000	0.66303800
H	-3.59855900	2.98996400	1.82821500
H	-3.97182800	2.08947200	0.34346500
C	-3.38310800	-1.22679800	-0.57225700
C	-2.54894200	-2.07957900	-1.55328400
H	-2.01629800	-2.89969100	-1.04202100
H	-3.20317300	-2.54789000	-2.31303100
H	-1.80535300	-1.47284700	-2.09761100
C	-4.44490300	-2.13969300	0.06991400
H	-5.09502200	-1.59569100	0.77075500
H	-5.09826900	-2.58009400	-0.70788300
H	-3.98860300	-2.97684800	0.62271000
C	-4.08292100	-0.11683800	-1.37738800
H	-3.36315500	0.60129300	-1.80525200
H	-4.65819800	-0.55008500	-2.21777400
H	-4.79548900	0.45003400	-0.75842200
N	1.98332000	-1.43463800	-0.07522100
C	1.16473000	-2.41890600	-0.80891200

H	0.16129500	-1.97017000	-0.80564900
C	3.30726400	-1.55503200	0.35252700
C	1.60606500	-2.56259300	-2.26654800
H	2.57772500	-3.07204700	-2.35555200
H	0.86362300	-3.15880800	-2.81894500
H	1.68124300	-1.57714900	-2.74537700
C	1.04974200	-3.75746200	-0.07691300
H	0.81542500	-3.60343600	0.98628500
H	0.23117300	-4.34179900	-0.52420400
H	1.96598400	-4.35968800	-0.15731000
C	4.16452300	-2.76398100	0.15864400
H	3.85862500	-3.59882600	0.80996700
H	4.13528400	-3.12687100	-0.87895400
H	5.21083500	-2.52702900	0.39293500
C	0.42297900	3.15282100	-1.91182300
H	0.55568000	4.06282700	-1.32020700
C	-0.79128300	2.98813700	-2.66468800
H	-1.57283200	3.75175600	-2.62338300
C	1.58721300	2.47011100	-2.40277100
H	2.58798100	2.85370000	-2.18416000
C	-1.69320900	-1.93048100	1.90664200
H	-1.44391700	-2.82492200	1.31334500
H	-0.81492600	-1.69457400	2.52839000
H	-2.52311200	-2.20669500	2.58036600

5

B3LYP(D3)/def2-SVP

E = -1659.658774 a.u.

Al	-0.28513200	-0.06845100	1.05306600
Si	-1.93463000	0.01059600	-0.81130600
N	2.31722200	-1.00438500	-0.40034900
C	1.54905400	0.02420900	0.02881700
C	1.95735700	-2.40846900	-0.10044800
H	0.95701100	-2.32758900	0.34721500
C	-0.25072900	1.15821200	2.71358200
H	-0.24446800	2.25562200	2.72351700
C	3.45094600	-0.53269400	-1.06589600
C	-1.52616700	0.53015500	3.23870900
H	-2.35347000	1.12844500	3.63170200
C	0.94121300	0.50734100	3.37981000
H	1.70734600	1.08751000	3.90490100
C	2.87306900	-3.00449900	0.96950100
H	3.90224500	-3.14794500	0.60669800
H	2.48611300	-3.98811100	1.27690500
H	2.89492200	-2.35196800	1.85392300
C	1.83315900	-3.26612000	-1.36018600
H	1.21690000	-2.76157900	-2.11870700
H	1.33643100	-4.21333600	-1.10182700
H	2.80897900	-3.51409100	-1.80120600
C	-2.89166100	-1.69203500	-0.99789800
C	-1.85597300	-2.74966700	-1.43306900
H	-1.42269900	-2.53137800	-2.42239000
H	-2.32633700	-3.74910100	-1.49479900
H	-1.02679300	-2.82917700	-0.71034400
C	4.49848700	-1.39060900	-1.69799300
H	4.14476400	-1.84843900	-2.63614400
H	4.82455900	-2.20371700	-1.03457900
H	5.38329500	-0.78688400	-1.94084700
C	-4.00691100	-1.63489500	-2.05724600
H	-4.83141400	-0.97407800	-1.74846100
H	-4.44130200	-2.64047800	-2.21618800
H	-3.64001600	-1.28036500	-3.03463800
C	-3.49393100	-2.14576600	0.34581500
H	-2.72193500	-2.25401800	1.12261100
H	-3.99307400	-3.12694600	0.23082800
H	-4.24454400	-1.43953400	0.72981500
C	-3.11413500	1.57224800	-0.68877900
C	-2.24336200	2.77902100	-0.27659400
H	-1.42395500	2.96269000	-0.99200500
H	-2.85299400	3.70176600	-0.24248100
H	-1.79917000	2.64260800	0.72218600
C	-3.78974200	1.90842000	-2.03225600
H	-4.43867700	1.09669800	-2.39173700

H	-4.42211900	2.81099500	-1.92876400
H	-3.05179500	2.11890600	-2.82337200
C	-4.19169200	1.36208200	0.39047900
H	-3.74959600	1.06388500	1.35532200
H	-4.76214800	2.29577600	0.55627100
H	-4.91867700	0.58786300	0.09906300
N	2.18154600	1.14986900	-0.37954600
C	1.65165000	2.49106300	-0.04348100
H	0.64034500	2.28451100	0.33219600
C	3.36589900	0.83767100	-1.04930800
C	2.43875800	3.12177700	1.10546700
H	3.47072900	3.36948100	0.81209400
H	1.94565800	4.05294200	1.42388300
H	2.46939000	2.43641700	1.96429900
C	1.50509100	3.40176100	-1.26315800
H	1.02550600	2.86897700	-2.09736100
H	0.86376000	4.25422600	-0.99399700

H	2.46535500	3.80898000	-1.60912800
C	4.30633400	1.83444000	-1.64476200
H	3.90513300	2.28053800	-2.56911000
H	4.53089300	2.65596800	-0.94960100
H	5.25748900	1.34819600	-1.90007400
C	-0.26714200	-1.42445900	2.60934300
H	-0.27808700	-2.51936500	2.53264500
C	-1.53405100	-0.82253100	3.18638100
H	-2.36960000	-1.43864900	3.53012500
C	0.93291800	-0.84419900	3.32640000
H	1.68828500	-1.47465700	3.80692000
C	-0.97397900	0.24085100	-2.46164800
H	-0.58236400	1.26834000	-2.53823300
H	-0.10934500	-0.43994100	-2.52184200
H	-1.60712300	0.06329700	-3.34709500

5. References

(S1) L. P. Press, A. J. Kosanovich, B. J. McCulloch, O. V. Ozerov, *J. Am. Chem. Soc.* **2016**, *138*, 9487-9497.

(S2) Y. Zhao, V. Snieckus, *Org. Lett.* **2014**, *16*, 3200-3203.

(S3) APEX suite of crystallographic software, APEX 4 version 2021.10-0; Bruker AXS Inc.: Madison, Wisconsin, USA, 2021.

(S4) SAINT, Version 7.56a and SADABS Version 2008/1; Bruker AXS Inc.: Madison, Wisconsin, USA, 2008.

(S5) Sheldrick, G. M. SHELXL-2014, University of Göttingen, Göttingen, Germany, 2014.

(S6) Hübschle, C. B.; Sheldrick, G. M.; Dittrich, B. *J. Appl. Cryst*. **2011**, *44*, 1281-1284.

(S7) Sheldrick, G. M. SHELXL-97, University of Göttingen, Göttingen, Germany, 1998.

(S8) Wilson, A. J. C. International Tables for Crystallography, Vol. C, Tables 6.1.1.4 (pp. 500-502), 4.2.6.8 (pp. 219-222), and 4.2.4.2 (pp. 193-199); Kluwer Academic Publishers: Dordrecht, The Netherlands, 1992.

(S9) Macrae, C. F.; Bruno, I. J.; Chisholm, J. A.; Edgington, P. R.; McCabe, P.; Pidcock, E.; Rodriguez-Monge, L.; Taylor, R.; van de Streek, J.; Wood, P. A. *J. Appl. Cryst*. **2008**, *41*, 466-470.

(S10) Frisch, M. J.; Trucks, G. W.; Schlegel, H. B.; Scuseria, G. E.; Robb, M. A.; Cheeseman, J. R.; Scalmani, G.; Barone, V.; Mennucci, B.; Petersson, G. A.; Nakatsuji, H.; Caricato, M.; Li, X.; Hratchian, H. P.; Izmaylov, A. F.; Bloino, J.; Zheng, G.; Sonnenberg, J. L.; Hada, M.; Ehara, M.; Toyota, K.; Fukuda, R.; Hasegawa, J.; Ishida, M.; Nakajima, T.; Honda, Y.; Kitao, O.; Nakai, H.; Vreven, T.; Montgomery, J. A., Jr.; Peralta, J. E.; Ogliaro, F.; Bearpark, M.; Heyd, J. J.; Brothers, E.; Kudin, K. N.; Staroverov, V. N.; Kobayashi, R.; Normand, J.; Raghavachari, K.; Rendell, A.; Burant, J. C.; Iyengar, S. S.; Tomasi, J.; Cossi, M.; Rega, N.; Millam, J. M.; Klene, M.; Knox, J. E.; Cross, J. B.; Bakken, V.; Adamo, C.; Jaramillo, J.; Gomperts, R.; Stratmann, R. E.; Yazyev, O.; Austin, A. J.; Cammi, R.; Pomelli, C.; Ochterski, J. W.; Martin, R. L.; Morokuma, K.; Zakrzewski, V. G.; Voth, G. A.; Salvador, P.; Dannenberg, J. J.; Dapprich, S.; Daniels, A. D.; Farkas, O.; Foresman, J. B.; Ortiz, J. V.; Cioslowski, J.; Fox, D. J., Gaussian 16, Revision A.03; Gaussian, Inc., Wallingford CT, **2016**.

(S11) (a) Becke, A. D. Density-functional thermochemistry. III. The role of exact exchange. *J. Chem. Phys.* **1993**, *98*, 5648-5652. (b) Stephens, P. J.; Devlin, F. J.; Chabalowski, C. F.; Frisch, M. J. Ab Initio Calculation of Vibrational Absorption and Circular Dichroism Spectra Using Density Functional Force Fields. *J. Phys. Chem*. **1994**, *98*, 11623-11627. (c) Grimme, S.; Antony, J.; Ehrlich, S.; Krieg, H. A consistent and accurate ab initio parameterization of density functional dispersion correction (DFT-D) for the 94 elements H-Pu. *J. Chem. Phys*. **2010**, *132*, 154104. (d) Grimme, S.; Ehrlich, S.; Goerigk, L. Effect of the damping function in dispersion corrected density functional theory. *J. Comput. Chem.* **2011**, *32*, 1456-1465.

(S12) Weigend F., Ahlrichs. R., Balanced basis sets of split valence, triple zeta valence and quadruple zeta valence quality for H to Rn: Design and assessment of accuracy. *Phys. Chem. Chem. Phys.* **2005**, *7*, 3297-305.

(S13) (a) Perdew, J. P. Density-functional approximation for the correlation energy of the inhomogeneous electron gas. *Phys. Rev. B* **1986**, *33*, 8822-8824; (b) Becke, A. D. Density-functional exchange-energy approximation with correct asymptotic behavior. *Phys. Rev. A* **1988**, *38*, 3098-3100.

(S14) (a) Fukui, K. Formulation of the reaction coordinate. *J. Phys. Chem*. **1970**, *74*, 4161-4163. Fukui, K. (b) The path of chemical reactions-the IRC approach. *Acc. Chem. Res.* **1981**, *14*, 363-368.
